# Safety and immunogenicity of an inactivated SARS-CoV-2 vaccine (FAKHRAVAC®) in healthy adults aged 18–55 years: Randomized, double-blind, placebo-controlled, phase I clinical trial

**DOI:** 10.1016/j.jvacx.2023.100401

**Published:** 2023-10-27

**Authors:** Akram Ansarifar, Ramin Hamidi Farahani, Ahmad Karimi Rahjerdi, Mohammadreza Ahi, Ali Sheidaei, Kimiya Gohari, Zahra Rahimi, Fatemeh Gholami, Pouria Basiri, Milad Moradi, Arash Jahangiri, Kosar Naderi, Soheil Ghasemi, Pezhman Khatami, Mohsen Honari, Samane Khodaverdloo, Mohammad Shooshtari, Hajar Mehr Azin, Sohrab Moradi, Batool Shafaghi, Hossein Allahyari, Arina Monazah, Ali Khodaei Poor, Hooman Bakhshande, Zahra Taghva, Mohammad Karimi Nia, Masoud Solaymani Dodaran, Mohsen Foroughizadeh

**Affiliations:** a Clinical Trial Center of Iran University of Medical Sciences, Tehran, Iran; b AJA University of Medical Sciences, Tehran, Iran; c Stem Cell Technology Research Center (STRC), Tehran, Iran; d Milad Daro Noor Pharmaceutical (MDNP) Company, Tehran, Iran; e Malek Ashtar University of Technology, Tehran, Iran

**Keywords:** Clinical trial, Safety, Phase I, Inactivated vaccine, Immunogenicity

## Abstract

**Background:**

The FAKHRAVAC®, an inactivated SARS-CoV-2 vaccine, was assessed for safety and immunogenicity.

**Methods and findings:**

In this double-blind, placebo-controlled, phase I trial, we randomly assigned 135 healthy adults between 18 and 55 to receive vaccine strengths of 5 or 10 μg/dose or placebo (adjuvant only) in 0–14 or 0–21 schedules. This trial was conducted in a single center in a community setting. The safety outcomes in this study were reactogenicity, local and systemic adverse reactions, abnormal laboratory findings, and Medically Attended Adverse Events (MAAE). Immunogenicity outcomes include serum neutralizing antibody activity and specific IgG antibody levels.

The most frequent local adverse reaction was tenderness (28.9%), and the most frequent systemic adverse reaction was headache (9.6%). All adverse reactions were mild, occurred at a similar incidence in all six groups, and were resolved within a few days. In the 10-µg/dose vaccine group, the geometric mean ratio for neutralizing antibody titers at two weeks after the second injection compared to the placebo group was 9.03 (95% CI: 3.89–20.95) in the 0–14 schedule and 11.77 (95% CI: 2.77–49.94) in the 0–21 schedule. The corresponding figures for the 5-µg/dose group were 2.74 (1.2–6.28) and 5.2 (1.63–16.55). The highest seroconversion rate (four-fold increase) was related to the 10-µg/dose group (71% and 67% in the 0–14 and 0–21 schedules, respectively).

**Conclusions:**

FAKHRAVAC® is safe and induces a strong humoral immune response to the SARS-CoV-2 virus at 10-µg/dose vaccine strength in adults aged 18–55. This vaccine strength was used for further assessment in the phase II trial.

Trial registration

This study is registered with https://www.irct.ir; IRCT20210206050259N1.

## Introduction

Inactivated vaccines are among the oldest vaccine types developed against viruses. Their production and scale-up are relatively simple and low cost, and therefore, traditionally, they have been the first line of attempt by vaccine developers. In the SARS-Cov-2 pandemic, inactivated vaccines were among the first that received WHO emergency approval and were used on a wide scale [1]. Inactivated vaccines, in comparison to other platforms, have advantages and disadvantages. Advantages include easy maintenance and transport, no virus replication, and fewer expected adverse reactions [2], [3], [4], [5], [6]. The disadvantages of the inactivated vaccine include a non-robust, short-lasting immune response requiring adjuvants and boosters and a longer vaccine production time [7], [8], [9]. The usual adjuvant used in inactivated vaccines is aluminum hydroxide, although, on one occasion, CpG oligodeoxynucleotides were also added to the combination (VLA-2001vaccine) [10], [11].

FAKHRAVAC® is an inactivated SARS-Cov-2 vaccine. We used the G614 strain isolated from oropharynx swabs of confirmed SARS-CoV-2 patients admitted to Iranian hospitals for vaccine production. We replicated the virus using Vero cells (Cat. # 88020401), a WHO-approved cell line and inactivated it with formaldehyde. Aluminum hydroxide was used as the adjuvant at 1.5 mg/ml concentration [12].

FAKHRAVAC humoral immunogenicity and safety were assessed in four mammalian animals (BALB/C mice, guinea pig, rabbit, and monkey) in the preclinical phase [12]. The results showed its potential for further clinical evaluation [12]. We performed a phase I clinical trial to assess FAKHRAVAC’s safety and immunogenicity in humans and to compare the two strengths of 5 μg/dose (0.5 × 10^6^ TCID_50_) and 10 μg/dose (2.5 × 10^6^ TCID_50_) administered in two schedules of 0–14 and 0–21.

## Methods

### Study design

We did a randomized, double-blind, placebo-controlled phase I clinical trial in healthy adults 18–55 years old. The first 15 people were enrolled in the study as sentinel subjects, and they were aware of the study groups they were assigned to (open-label). The allocation of the remaining 120 participants into 6 study groups was randomized and blind. There were two strengths of 5 and 10 µg/dose inactivated SARS-CoV-2 vaccine FAKHRAVAC® and placebo groups (receiving adjuvant-only preparation) administered in two schedules of 0–14 and 0–21. This trial was conducted in a single center in a community setting.

This study was conducted in collaboration with the Iran University of Medical Science (IUMS) clinical trial center as the academic contract research organization (CRO). The study protocol was approved by the National Research Ethics Committee (approval number IR. NREC.1399.006, 28th February 2021). Patients or the public were not involved in the design, or conduct, or reporting, or dissemination plans of our research.

### Participants

After approval by the Food and Drug organization, Iranian participants aged 18–55 years were invited to the study via a website. Participants who digitally signed (accepted) the informed consent form could complete the initial screening questionnaire online. Eligible candidates in the online screening were invited to attend the trial center, signed a paper informed consent, and further evaluated for eligibility. Inclusion criteria were: psychologically competent to volunteer for trial, age 18 to 55 years, body mass index between 18 and 35 kg/meter2, having complete health based on clinical and laboratory criteria, no current or previous SARS-CoV-2 disease, no pregnancy, negative IgG and IgM antibody titers against SARS-CoV-2 N antigen, negative RT-PCR test for SARS-CoV-2, IgG ELISA negative blood test against HIV, heart rate between 60 and 100, systolic blood pressure (between 90 and 140 mm Hg), diastolic blood pressure (between 60 and 90 mm Hg), use a safe method of contraception in men and women up to 3 months after the last injection of the vaccine. The main exclusion criteria were: positive RT-PCR test and presence of detectable antibody against SARS-CoV-2 nucleocapsid antigen; pregnancy; breastfeeding; history of syncope during injection or phlebotomy; history of immunodeficiency disorders (suspected or definite); long-term use of immunosuppressive drugs; history of allergic diseases such as angioedema or anaphylaxis; receiving chemotherapy or radiotherapy in the last five years; alcohol or drug abuse; any close contact with a person with confirmed SARS-CoV-2 in the two weeks leading up to the first vaccine injection. A complete list of eligibility criteria is available in the Study protocol (see S4 Protocol).

### Randomization and masking

This study utilized the variable size permuted block randomization method. The first 15 people entered the study gradually and in batches of 3 or 4 over nine days and were not blinded. The remaining 120 participants were randomly allocated into six groups, including a placebo group and two vaccine strengths of 5 and 10 µg/dose in two 0–14 and 0–21 schedules.

The randomization codes were generated by the rand() function and Excel software. Each participant was assigned a non-repeating four-digit number (randomization code) to conceal the randomization sequence. The study epidemiologist generated the random sequence.

The identical vaccine and placebo single-injection vials were labeled by an independent team using the randomization codes. These codes were also embedded in the study software and successively assigned to the eligible participants. The 5 and 10 μg/dose strengths and placebo vials were the same shapes, color, and size. Thus, all participants, investigators, laboratory staff, and statisticians were unaware of the group allocations.

### Procedures

After assessing vital signs and physical examination on day zero, each participant received the vaccine or the placebo based on the randomization sequence. Participants reported their vital signs and local (pain, tenderness, swelling, and redness) and systemic (nausea/vomiting, diarrhea, headache, fatigue, and myalgia) adverse reactions during the seven days after each injection. All of the participants were invited to visit the research center for face to face assessment of safety and collecting blood samples for immunogenicity by study officers on days 7, 14, 21, 28, 42, 72, 90, 180, in the 0–14 schedule, and days 7, 14, 21, 28, 35, 49, 90, 180 in the 0–21 schedule. Follow-up at the second, fourth, and fifth-month intervals was conducted through phone calls (see S4 Protocol).

### Outcomes

The primary outcome was safety profile, including reactogenicity, local and systemic adverse reactions, and abnormal laboratory findings one week after injection. The secondary outcomes were serum neutralizing antibody activity measured by conventional virus neutralization test (for details of cVNT methodology please see the supplement 4) and specific IgG antibody levels, Medically Attended Adverse Events (MAAE), Serious Adverse Event (SAE), and Suspected Unexpected Serious Adverse Reactions (SUSAR).

We asked the participants to report daily by filling out a self-administered questionnaire for seven days after injection and bringing it to the study center on their next visit. The research team made daily telephone contact with study participants during this period. All the items in the daily questionnaire were checked to ensure that they had been properly completed. Study participants could contact resident physicians at the center during the study period 24/7 if they needed any assistance or consultation and report any possible medical condition.

Serum neutralizing antibody activity was assessed using a conventional virus neutralization test (cVNT). Specific IgG antibody against S antigen was measured by enzyme-linked immunosorbent assay (ELISA). We measured peripheral blood cytokine levels for IL-2, IL-4, IL-5, IL-6, IL-10, IL-12, IL-17, TNF-α, and γ-INF two weeks after the second injection to assess the inflammatory response. CD3, CD4, CD8, CD4/CD8, CD56, CD19 and CD20 were evaluated using flow cytometry.

### Patient and public involvement

Patients and/or the public were not involved in this research's design, conduct, reporting, or dissemination plans.

### Statistical analysis

The sample size was 135 based on practical considerations and expert opinion. We assessed the safety outcomes in all participants who received at least one vaccine or placebo injection and the immunogenicity outcomes in all participants who received two vaccine or placebo injections. The primary approach for analysis was the intention to treat.

Baseline comparisons were performed to examine the homogeneity between the study groups. For immunogenicity assessment, we calculated Geometric Mean Titers (GMT), Geometric Mean Ratio (GMR), Geometric Mean Fold Increase (GMFI), and their corresponding 95 % CIs based on a standard normal distribution of the log-transformations of antibody titers. A four-fold increase in antibody titers was considered seroconversion [13]. The data were analyzed by R 3.4.2 and Stata 11 (Stata Corporation, Texas, USA). The study was conducted under oversight from an 8-members data monitoring committee, including representatives from local regulatory authorities and the national ethics committee, and registered with the Iranian Registry of Clinical Trial (Code: IRCT20210206050259N1).

## Results

**Fig. 1:**
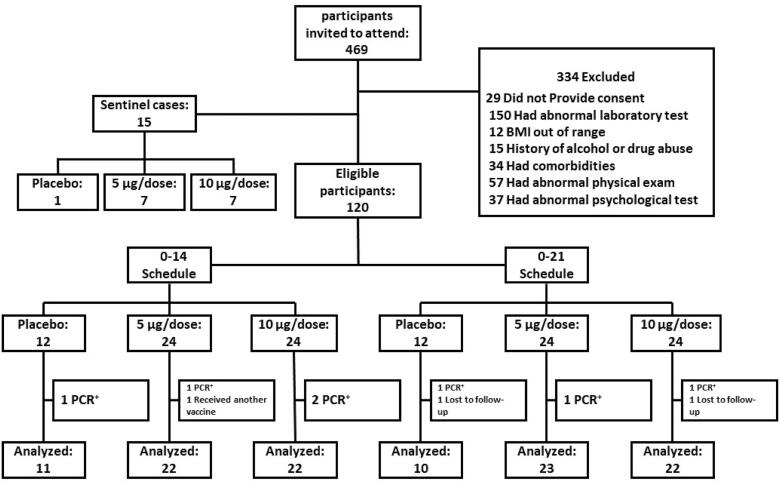
Participant flow diagram.

Between the 16th of March and the 13th of April 2021, 469 volunteers aged 18–55 years old were invited and screened for eligibility in phase I, of which 135 eligible participants were recruited into the trial. The first 15 participants were considered sentinel, and 120 remaining participants were randomly assigned to vaccine groups or placebo in two injection schedules of 0–14 and 0–21 (Fig. 1).

The mean age of all 135 participants was 35 years (SD 8.6; range 19–54), with 35 (25.9 %) individuals aged 18–30 years, 62 (45.9 %) aged 31–40 years, and 31 (23 %) aged 41–50 years, and 7 (5.2 %) aged 51 years or older across the groups. In this study, 69.6 % (94/135) of participants were male. Baseline characteristics of the participants were broadly similar across the groups (Table 1).

**Table 1:** Baseline characteristics.

		Placebo, 0–14	5 µg/dose, 0–14	10 µg/dose, 0–14	Placebo, 0–21	5 µg/dose, 0–21	10 µg/dose, 0–21	Total
No. of participants		**12**	**24**	**24**	**12**	**24**	**24**	**120**
Sex, n(%)								
	Male	8 (66.67 %)	12 (50 %)	17 (70.83 %)	10 (83.33 %)	17 (70.83 %)	15 (62.5 %)	79 (65.83 %)
	Female	4 (33.33 %)	12 (50 %)	7 (29.17 %)	2 (16.67 %)	7 (29.17 %)	9 (37.5 %)	41 (34.17 %)
Age, yr								
	Mean (SD)	34.5 (10.95 %)	35.29 (6.58 %)	34.88 (9.42 %)	34 (9.90 %)	32.75 (10.27 %)	37.96 (6.97 %)	35.03 (8.85 %)
Body-mass index								
	Mean (SD)	24.16 (4.38 %)	24.65 (3.23 %)	25.59 (4.12 %)	26.71 (3.64 %)	25.86 (3.70 %)	25.84 (4.99 %)	25.67 (4.01 %)
Smoking, n(%)								
	Current Smoker	2 (17 %)	1 (4.2 %)	2 (8.3 %)	6 (50 %)	3 (12 %)	5 (21 %)	19 (15.83 %)
	Ex-smoker	0 (0 %)	1 (4.2 %)	5 (21 %)	0 (0 %)	4 (17 %)	4 (17 %)	14 (11.67 %)
	Never-smoker	10 (83 %)	22 (92 %)	17 (71 %)	6 (50 %)	17 (71 %)	15 (62 %)	87 (72.5 %)
Education, n(%)								
	Elementary	1 (8.3 %)	0 (0 %)	0 (0 %)	1 (8.3 %)	0 (0 %)	1 (4.2 %)	3 (2.5 %)
	Diploma	3 (25 %)	6 (25 %)	7 (29 %)	3 (25 %)	10 (42 %)	7 (29 %)	36 (30 %)
	Bachelor	6 (50 %)	10 (42 %)	7 (29 %)	5 (42 %)	10 (42 %)	9 (38 %)	47 (39.17 %)
	Master	2 (17 %)	5 (21 %)	9 (38 %)	3 (25 %)	3 (12 %)	5 (21 %)	27 (22.5 %)
	Doctoral and above	0 (0 %)	3 (12 %)	1 (4.2 %)	0 (0 %)	1 (4.2 %)	2 (8.3 %)	7 (5.83 %)

The safety findings show a favorable profile for this vaccine. No suspected unexpected serious adverse reactions (SUSAR) were observed in this study. The three patients with serious adverse events included hospitalizations due to SARS-CoV-2, lumbar disc herniation, and diagnostic curettage. All three were discharged in good general condition. Also, the only hospitalized patient due to SARS-CoV-2 belonged to the placebo group.

**Fig. 2:**
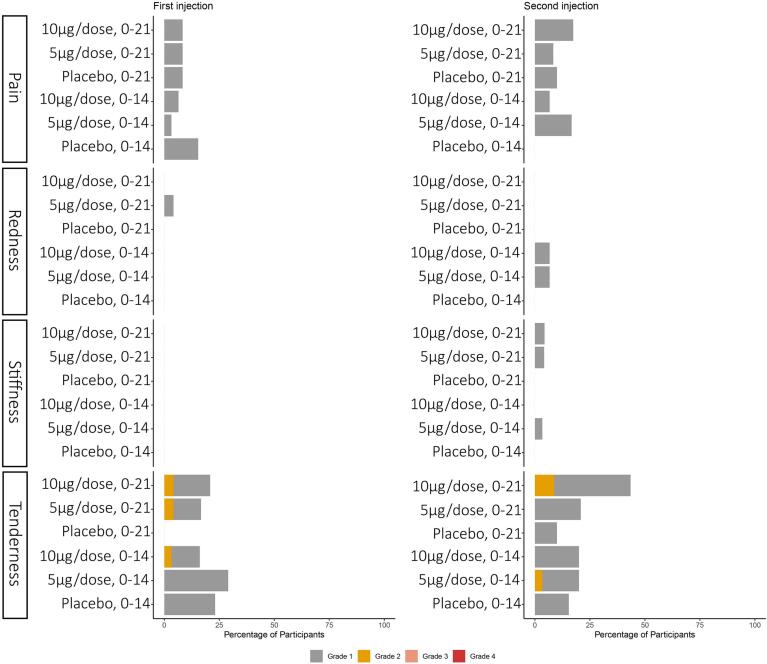
The proportions of participants with local adverse reactions within seven daysprotocol.

**Fig. 3:**
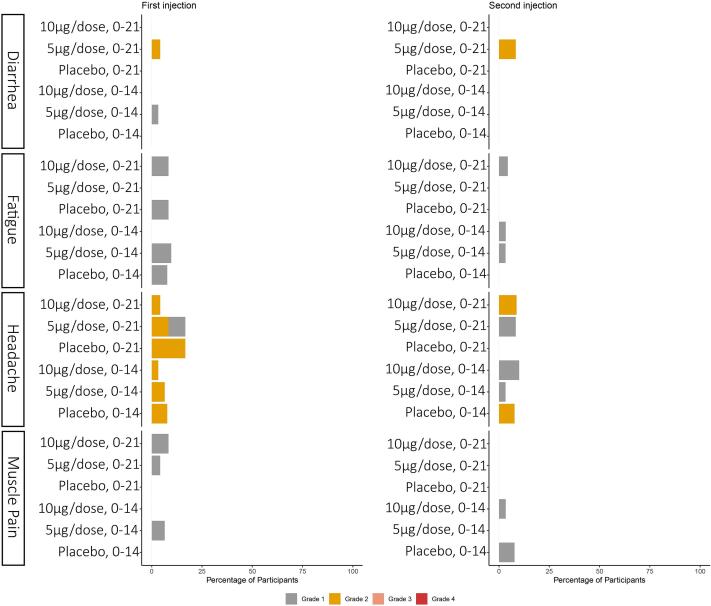
The proportions of participants with systemic adverse reactions within seven days.

In this study, 41.5 % (56/135) of participants reported at least one local adverse reaction seven days after the injection. The most common local adverse reaction was tenderness (28.9 %). Participants also reported pain (14.8 %), redness (3.7 %) and swelling/stiffness (2.2 %). In addition, 25.9 % (35/135) of participants reported at least one systemic adverse reaction within seven days after the injection. The most common reported systemic reaction was headache (9.6 %). Participants also reported Diarrhea (1.5 %), Fatigue(6.7 %), Myalgia(5.2 %), and None of the 135 participants reported Nausea/vomiting (Fig. 2, Fig. 3).

**Fig. 4:**
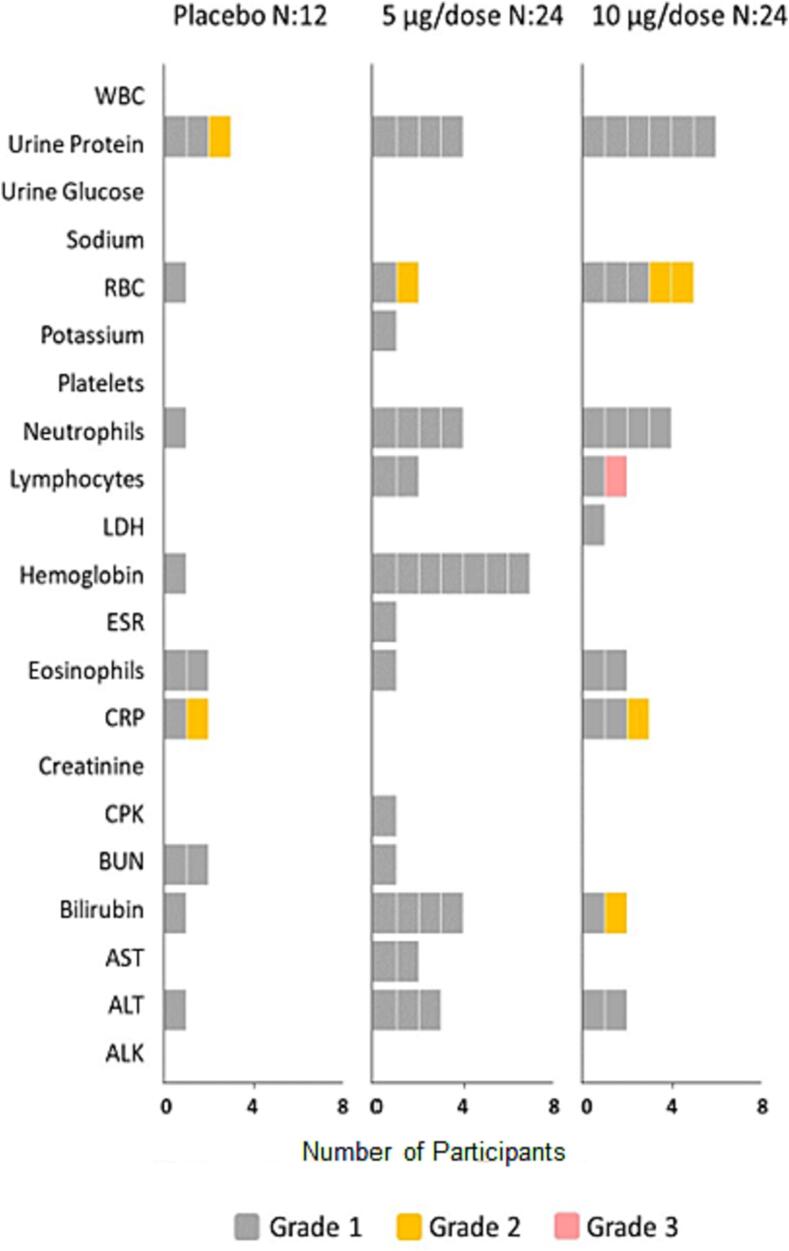
The number of participants with abnormal laboratory findings seven days after injection.

A total of 73 abnormal grade I and II laboratory results were observed in safety evaluation one week after the vaccination. One grade III lymphocytopenia was reported in a participant who received 10-µg/dose vaccine strength, and no grade IV was seen (Fig. 4). All laboratory abnormalities were followed until they returned to normal. Serum levels of inflammatory lymphokines and peripheral blood lymphocytic subsets 14 days after the second injection did not change from the baseline.

**Fig. 5:**
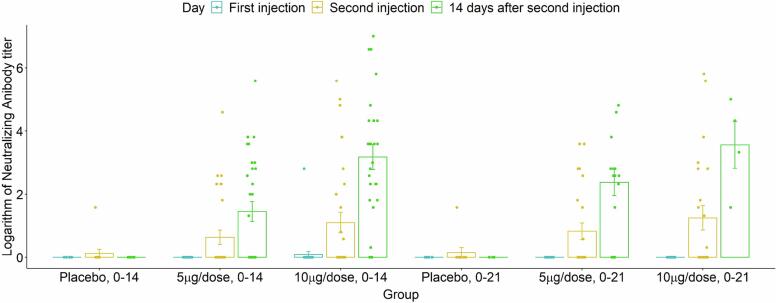
The geometric mean titers for serum neutralizing antibodies in study groups over time.

The serum neutralizing activity was undetectable (titer < 4) at baseline (before the first injection) in 134 of 135 participants. Two weeks after the second injection, participants in the 10 μg/dose group had a GMT of 9.03 (95 % CI: 5.17–15.8) in the 0–14 schedule and 11.77 (95 % CI: 2.29–60.52) in the 0–21 schedule. No increase in serum neutralizing activity was observed in the placebo groups. GMR and GMFI for 10 μg/dose group were 9.03 (95 % CI: 3.89–20.95) and 8.13 (95 % CI: 4.6–14.4) in the 0–14 schedule, and 11.77 (95 % CI: 2.77–49.94) and 8.43 (95 % CI: 0.78–91.56) in the 0–21 schedule, respectively. At 14 days after the second injection, the seroconversion (four-fold increase) rates of neutralizing antibodies were 71 % and 67 % in the 0–14 and 0–21 schedules, respectively (Table 2, Table 3, and Fig. 5).

**Table 2:** Serum neutralizing antibody indices in study groups over time.

Group		Day 0	2nd injection	2 wks after 2nd injection	4 wks after 2nd injection
GMT					
	Placebo, 0–14	1 (1–1, N: 10)	1.1 (0.9–1.34, N: 12)	1 (1–1, N: 11)	1.07 (0.9–1.27, N: 6)
	5 µg/dose, 0–14	1 (1–1, N: 24)	1.56 (1.13–2.15, N: 29)	2.74 (1.76–4.28, N: 29)	3.34 (2.06–5.41, N: 25)
	10 µg/dose, 0–14	1.07 (0.93–1.23, N: 29)	2.15 (1.35–3.41, N: 30)	9.03 (5.17–15.8, N: 26)	6.21 (3.32–11.64, N: 14)
	Placebo, 0–21	1 (1–1, N: 9)	1.12 (0.87–1.43, N: 10)	1 (1–1, N: 4)	1.2 (0.77–1.85, N: 7)
	5 µg/dose, 0–21	1 (1–1, N: 19)	1.78 (1.22–2.59, N: 24)	5.2 (2.8–9.65, N: 14)	3.42 (1.54–7.57, N: 12)
	10 µg/dose, 0–21	1 (1–1, N: 21)	2.39 (1.37–4.16, N: 23)	11.77 (2.29–60.52, N: 4)	4.71 (2.39–9.27, N: 16)
GMR					
	Placebo, 0–14	1	1	1	1
	5 µg/dose, 0–14	1 (0.83–1.2, N:10) 0.501^a^	1.42 (0.72–2.79, N:12) 0.148	2.74 (1.2–6.28, N:11) 0.008	3.12 (1.17–8.35, N:6) 0.012
	10 µg/dose, 0–14	1.07 (0.89–1.28, N:24)	1.96 (1–3.83, N:29)	9.03 (3.89–20.95, N:29)	5.81 (2.02–16.69, N:25)
		0.233	0.289	< 0.001	< 0.001
	Placebo, 0–21	1	1	1	1
	5 µg/dose, 0–21	1 (1–1, N:19)	1.59 (0.74–3.43, N:24)	5.2 (1.63–16.55, N:14)	2.86 (0.93–8.75, N:12)
		>0.999	0.118	0.003	0.508
	10 µg/dose, 0–21	1 (1–1, N:21)	2.14 (0.99–4.62, N:23)	11.77 (2.77–49.94, N:4)	3.94 (1.35–11.45, N:16)
		>0.999	0.405	< 0.001	0.006
GMFI					
	Placebo, 0–14	1	1.12 (0.87–1.43, N: 10)	1 (1–1, N: 9)	1.11 (0.8–1.53, N: 4)
			0.186	>0.999	0.264
	5 µg/dose, 0–14	1	1.51 (1.04–2.18, N: 23)	2.51 (1.54–4.07, N: 24)	3.5 (2.07–5.91, N: 20)
			0.015	<0.001	<0.001
	10 µg/dose, 0–14	1	2.12 (1.35–3.32, N: 28)	8.13 (4.6–14.4, N: 24)	6.23 (3.14–12.34, N: 13)
			<0.001	<0.001	<0.001
	Placebo, 0–21	1	1.15 (0.83–1.59, N: 8)	1 (1–1, N: 2)	1.2 (0.77–1.85, N: 7)
			0.201	>0.999	0.207
	5 µg/dose, 0–21	1	1.71 (1.1–2.67, N: 19)	5.31 (2.56–11.02, N: 12)	3.42 (1.54–7.57, N: 12)
			0.009	<0.001	0.001
	10 µg/dose, 0–21	1	2.35 (1.3–4.25, N: 21)	8.43 (0.78–91.56, N: 3)	4.71 (2.39–9.27, N: 16)
			0.002	>0.999	<0.001

a The P-values in this table are achieved from the Wald statistics of the regression approach.

**Table 3:** The proportion of participants with a four-fold increase in neutralizing antibody titer.

Group	Day 0	2nd injection	2 wks after 2nd injection	4 wks after 2nd injection
Placebo, 0–14 %(n)	Ref	0 % (12)	0 % (11)	0 % (6)
5 µg/dose, 0–14 %(n)	Ref	17 % (29)	42 % (29)	45 % (25)
10 µg/dose, 0–14 %(n)	Ref	29 % (30)	71 % (26)	62 % (14)
Placebo, 0–21 %(n)	Ref	0 % (10)	0 % (4)	0 % (7)
5 µg/dose, 0–21 %(n)	Ref	21 % (24)	75 % (14)	60 % (12)
10 µg/dose, 0–21 %(n)	Ref	24 % (23)	67 % (3)	50 % (16)

## Discussion

The inactivated FAKHRAVAC was tolerated well in all study groups. No vaccine-related serious adverse event was observed following the vaccination and subsequent follow-up period in any vaccine groups and administration schedules. Those who received two injections of the 10-µg/dose vaccine strength developed neutralizing antibody titers about nine times more than those who received the placebo two weeks after the second injection, and this was remarkably higher than the response in the 5-µg/dose vaccine recipients. The observed local and systemic reactions were generally mild and transient.

Local and systemic adverse events were predominantly mild (grades I and II) and resolved within a few days. There was no clinically significant difference in the safety profile between the study groups, and the findings were similar to that of other SARS-CoV-2 vaccines [14], [15], [16], [17], [18], [19]. There was only one case of grade III Lymphocytopenia in our study that was followed until resolution. Abnormal high-grade laboratory results have also been reported in other inactivated SARS-CoV-2 vaccines [15], [20].

We used fast protein liquid chromatography (FPLC) for vaccine purification, which is highly capable of separating host cell protein and host cell DNA impurities [21], [22]. We believe this partly explains the relatively low rates of side effects in study participants.

The vaccine elicited a safe immune response. Peripheral blood cytokine levels for IL-2, IL-4, IL-5, IL-6, IL-10, IL-12, IL-17, TNF-α, and γ-INF two weeks after the second injection showed no noticeable change from the baseline that reflects the ability of the immune system to self-contain the inflammatory process expected to be induced by vaccine injection (see supplement 4, Figures 16–24). Furthermore, the flow cytometric examination of peripheral blood immune cell composition showed no considerable changes in the number and proportion of the cells. (see supplement 3, Figures 25–32). Other studies on inactivated vaccines have also reported similar observations in their flow-cytometric and cytokine assessments in the peripheral blood [20].

We used a conventional virus neutralization test to assess specific antibody responses to SARS-COV-2 antigens. The test was conducted in a biosafety level III laboratory using a wild-type strain of the SARS-COV-2 virus isolated from the nasopharynx of Iranian hospitalized patients. Specific antibodies were also assessed by a commercially available kit (DiaZist®) and another kit developed in-house. However, we decided to draw conclusions based on the cVNT test results because of the inconsistencies in the results and questionable quality. As neutralizing antibody assessment was done by the same strain of the virus used for vaccine production and the SARS-COV-2 genome continues to change as a result of repeated mutations, we expect to lose some of the neutralizing power of FAKHRAVAC by the emergence of new strains of the virus. Therefore, in future use of this or any other SARS-COV-2 vaccine, updated COVID-19 vaccines based on prevailing strains will be needed.

Our study had some limitations: First, because of ethical considerations due to inadequate antibody response, participants in the placebo and the 5-µg/dose groups were advised to enter the national SARS-CoV-2 vaccination program once it became available in late 2021. Therefore, some did not complete the six-month study follow-up period. Incomplete follow-up in the placebo group did not interfere with the dose selection decision, as it had been made following the interim analysis four weeks after the second injection. Second, we did not compare neutralizing antibody titers in our study with the convalescent serum samples as positive controls, which could have provided clues to the magnitude of the antibody responses. Third, the sample size in each group was relatively small as it is a common limitation among most phase I vaccine studies.

FAKHRAVAC® at 10-µg/dose strength was safe and had a well-induced humoral immune response to the SARS-CoV-2 virus in adults aged 18–55 years in 0–14 and 0–21 schedules. It was selected for further clinical evaluation in a phase II clinical trial.

**Why was this study done?**

• Severe acute respiratory syndrome coronavirus 2 (SARS-CoV-2) has caused more than 573 million cases of SARS-CoV-2 worldwide, resulting in close to 6.4 million deaths so far.
• We assessed the safety and immunogenicity of FAKHRAVAC inactivated SARS-Cov-2 in phase I clinical trial

**What did the researchers do and find?**

• Study participants received two strengths of 5 μg/dose (0.5 × 106 TCID50) and 10 μg/dose (2.5 × 106 TCID50) and placebo in two 0–14 and 0–21 administration schedules and underwent an intensive follow-up for six months.
• Mild adverse reactions, such as pain, tenderness, and headache, were observed, and no severe adverse reaction was reported in all groups.
• The 10 μg strengths of the vaccine delivered in the 0–14 schedule resulted in 9 fold increase in neutralizing antibody titer compared to the placebo group.
• Administration schedules had little effect on efficacy.

**What do these findings mean?**

• FAKHRAVAC inactivated SARS-Cov-2 vaccine is safe and induces a strong immune response against SARS-CoV-2 disease.

## CRediT authorship contribution statement

**Akram Ansarifar:** Conceptualization, Investigation, Validation, Writing – original draft. **Ramin Hamidi Farahani:** Methodology. **Ahmad Karimi Rahjerdi:** Project administration. **Mohammadreza Ahi:** Investigation, Validation, Writing – original draft. **Ali Sheidaei:** Data curation, Formal analysis, Investigation, Validation, Visualization, Writing – original draft. **Kimiya Gohari:** Data curation, Formal analysis, Investigation, Validation, Visualization, Writing – original draft. **Zahra Rahimi:** Investigation, Validation, Writing – original draft. **Fatemeh Gholami:** Investigation, Validation, Writing – original draft. **Pouria Basiri:** Investigation. **Milad Moradi:** Investigation. **Arash Jahangiri:** Investigation. **Kosar Naderi:** Project administration. **Soheil Ghasemi:** Investigation. **Pezhman Khatami:** Investigation. **Mohsen Honari:** Investigation. **Samane Khodaverdloo:** Investigation. **Mohammad Shooshtari:** Investigation. **Hajar Mehr Azin:** Investigation. **Sohrab Moradi:** Investigation. **Batool Shafaghi:** Investigation. **Hossein Allahyari:** Investigation. **Arina Monazah:** Investigation. **Ali Khodaei Poor:** Investigation. **Hooman Bakhshande:** Supervision. **Zahra Taghva:** Investigation. **Mohammad Karimi Nia:** Investigation. **Masoud Solaymani Dodaran:** Conceptualization, Investigation, Methodology, Project administration, Validation, Visualization, Writing – original draft. **Mohsen Foroughizadeh:** Conceptualization, Methodology.

## Declaration of Competing Interest

The authors declare the following financial interests/personal relationships which may be considered as potential competing interests: SGH, PKH, MH, SKH, MSH, HM, SM, BSH, HA, AM, and AKH are employees of Milad Daro Noor Pharmaceutical (MDNP). MSD is an Iran University of Medical Science (IUMS) employee. MSD, AA, MA, ASH, ZR, FGH, and KG are members of the clinical trial center of the Iran University of Medical Science that acted as academic CRO. All other authors declare no other competing interests.

## Data Availability

Data will be made available on request.
