# Supplementary material for: Safety and immunogenicity of an inactivated SARS-CoV-2 vaccine (FAKHRAVAC®) in healthy adults aged 18–55 years: Randomized, double-blind, placebo-controlled, phase I clinical trial
Source: Vaccine X. 2023 Oct 27;15:100401. doi: 10.1016/j.jvacx.2023.100401 (PMC10628354; doi:10.1016/j.jvacx.2023.100401)

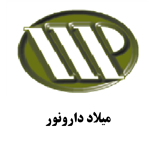

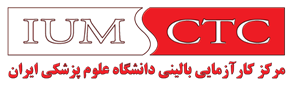


**Phase I trial of safety, immunogenicity and dose finding for two strengths of 0.5 × 10^6^ and 2.5 × 10^6^ (TCID50) inactivated SARS-CoV-2 vaccine FAKHRAVAC (MIVAC) injected in two schedules of two doses, 2 and 3 weeks apart in healthy adults aged 18-55 years: a randomized, double-blind, placebo-controlled clinical trial**

## Contents

[Contents 2](#_Toc106008176)

[List of tables 6](#_Toc106008177)

[List of figures 7](#_Toc106008178)

[List of abbreviations 8](#_Toc106008179)

[ADMINISTRATIVE INFORMATION 10](#_Toc106008180)

[Scientific title 10](#_Toc106008181)

[Public title 10](#_Toc106008182)

[Protocol registration 10](#_Toc106008183)

[Protocol version 10](#_Toc106008184)

[Funding source 10](#_Toc106008185)

[Roles and responsibilities 11](#_Toc106008186)

[Protocol contributors 11](#_Toc106008187)

[Funding 12](#_Toc106008188)

[Sponsor 12](#_Toc106008189)

[Responsibilities of sponsor 12](#_Toc106008190)

[Steering Committee 13](#_Toc106008191)

[Responsibilities of steering committee 13](#_Toc106008192)

[Chief investigator 14](#_Toc106008193)

[Responsibilities of chief investigator 14](#_Toc106008194)

[Co-investigators 15](#_Toc106008195)

[Responsibilities of Co-investigators 16](#_Toc106008196)

[Data and Safety Monitoring Board (DSMB) 16](#_Toc106008197)

[Responsibilities of DSMB 17](#_Toc106008198)

[Contract Research Organization (CRO) 18](#_Toc106008199)

[Responsibilities of CRO 18](#_Toc106008200)

[Monitoring and auditing 19](#_Toc106008201)

[Protocol synopsis 20](#_Toc106008202)

[Title 20](#_Toc106008203)

[Introduction 20](#_Toc106008204)

[Objectives 20](#_Toc106008205)

[Objectives in Phase I 21](#_Toc106008206)

[Interventions 21](#_Toc106008207)

[Outcomes 21](#_Toc106008208)

[Primary outcomes in Phase I 21](#_Toc106008209)

[Secondary outcomes in Phase I 22](#_Toc106008210)

[Methodology 22](#_Toc106008211)

[Trial design 22](#_Toc106008212)

[Inclusion criteria 22](#_Toc106008213)

[Exclusion criteria 23](#_Toc106008214)

[Withdrawal criteria 24](#_Toc106008215)

[Vaccination pause rules 24](#_Toc106008216)

[Recruitment strategy 25](#_Toc106008217)

[Sample size 25](#_Toc106008218)

[Statistical methods 25](#_Toc106008219)

[Ethical consideration 25](#_Toc106008220)

[Introduction and background 27](#_Toc106008221)

[Vaccination in COVID-19 27](#_Toc106008222)

[COVID-19 vaccine platforms 27](#_Toc106008223)

[Milad inactivated vaccine against COVID-19 (FAKHRAVAC) 28](#_Toc106008224)

[Vaccine specifications 29](#_Toc106008225)

[Preclinical studies 29](#_Toc106008226)

[Introduction 29](#_Toc106008227)

[Virus cultivation and inactivation 30](#_Toc106008228)

[Quality control 31](#_Toc106008229)

[Immunogenicity in laboratory animal models 33](#_Toc106008230)

[Antigen content determination 33](#_Toc106008231)

[Immunization protocol 34](#_Toc106008232)

[Vaccine safety 36](#_Toc106008233)

[Acute toxicity testing in animals 37](#_Toc106008234)

[Vaccine efficacy (viral challenge testing) 38](#_Toc106008235)

[Drug distribution and metabolic pathways in laboratory animals 38](#_Toc106008236)

[Stability studies 39](#_Toc106008237)

[Pyrogenicity testing 40](#_Toc106008238)

[Sterility testing 40](#_Toc106008239)

[Summary of pre-clinical studies 40](#_Toc106008240)

[Aim 41](#_Toc106008241)

[Phase I objectives 41](#_Toc106008242)

[Trial design 42](#_Toc106008243)

[Trial design in Phase I 42](#_Toc106008244)

[Methods: Participants, interventions and outcomes 43](#_Toc106008245)

[Study setting 43](#_Toc106008246)

[Inclusion and Exclusion (Eligibility) criteria in Phase I 43](#_Toc106008247)

[Inclusion criteria in Phase I 43](#_Toc106008248)

[Exclusion criteria in Phase I 45](#_Toc106008249)

[Withdrawal criteria in Phase I 46](#_Toc106008250)

[Interventions 47](#_Toc106008251)

[Group 1: Receiving 0.5× 10^6^ (TCID_50_) vaccine dose at 0 -14 47](#_Toc106008252)

[Group 2: Receiving 2.5 × 10^6^ (TCID_50_) vaccine dose at 0-14 48](#_Toc106008253)

[Group 3: Receiving placebo at 0-14 48](#_Toc106008254)

[Group 4: Receiving 0.5× 10^6^ (TCID_50_) vaccine dose at 0-21 48](#_Toc106008255)

[Group 5: Receiving 2.5 × 10^6^ (TCID_50_) vaccine dose at 0-21 48](#_Toc106008256)

[Group 6: Receiving placebo at 0-21 48](#_Toc106008257)

[Storage and preparation 48](#_Toc106008258)

[Administration route 48](#_Toc106008259)

[Vaccination pause rules 49](#_Toc106008260)

[Adherence monitoring strategies 49](#_Toc106008261)

[Outcomes 50](#_Toc106008262)

[Primary outcomes in Phase I 50](#_Toc106008263)

[Abnormal vital signs and anaphylactic reactions immediately after vaccination 50](#_Toc106008264)

[Local adverse events within the first week post-vaccination 50](#_Toc106008265)

[Systemic adverse event within the first week post-vaccination 50](#_Toc106008266)

[Abnormal laboratory findings 51](#_Toc106008267)

[Secondary outcomes in Phase I 53](#_Toc106008268)

[SAEs, SUSARs, MAAEs, up to 6 months after last vaccine dose 53](#_Toc106008269)

[Occurrence of COVID-19 disease two weeks after second vaccine dose 53](#_Toc106008270)

[Serum ELISA IgG level for SARS-CoV-2 N and S antigens 53](#_Toc106008271)

[Neutralizing antibody activity 54](#_Toc106008272)

[Cell-mediated immunity 54](#_Toc106008273)

[Study conduct 54](#_Toc106008274)

[Recruitment Strategy 55](#_Toc106008275)

[Screening 55](#_Toc106008276)

[Online screening 55](#_Toc106008277)

[On-site screening 56](#_Toc106008278)

[Obtaining inform consent 56](#_Toc106008279)

[Psychological assessment 56](#_Toc106008280)

[Clinical assessment 57](#_Toc106008281)

[Laboratory assessment 57](#_Toc106008282)

[Sentinel participants 57](#_Toc106008283)

[Participant visit plans in Phase I 58](#_Toc106008284)

[Participant visit plans in schedule 1 (0-14) 59](#_Toc106008285)

[Schedule 1, day -7, screening visit 61](#_Toc106008286)

[Schedule 1, day 0, visit 1 61](#_Toc106008287)

[Schedule 1, day 7, visit 2 62](#_Toc106008288)

[Schedule 1, day 14, visit 3 62](#_Toc106008289)

[Schedule 1, day 21, visit 4 62](#_Toc106008290)

[Schedule 1, day 28, visit 5 63](#_Toc106008291)

[Schedule 1, day 42, visit 6 63](#_Toc106008292)

[Schedule 1, day 72, visit 7 63](#_Toc106008293)

[Schedule 1, month 3, visit 8 64](#_Toc106008294)

[Schedule 1, month 6, visit 9 64](#_Toc106008295)

[Participant visit plans in schedule 2 (0-21) 65](#_Toc106008296)

[Schedule 2, day -7, screening visit 67](#_Toc106008297)

[Schedule 2, day 0, visit 1 67](#_Toc106008298)

[Schedule 2, day 7, visit 2 67](#_Toc106008299)

[Schedule 2, day 14, visit 3 68](#_Toc106008300)

[Schedule 2, day 21, visit 4 68](#_Toc106008301)

[Schedule 2, day 28, visit 5 69](#_Toc106008302)

[Schedule 2, day 35, visit 6 69](#_Toc106008303)

[Schedule 2, day 49, visit 7 69](#_Toc106008304)

[Schedule 2, month 3, visit 8 70](#_Toc106008305)

[Schedule 2, month 6, visit 9 70](#_Toc106008306)

[Sample size 70](#_Toc106008307)

[Methods: Assignment of interventions 71](#_Toc106008308)

[Random Allocation 71](#_Toc106008309)

[Concealment 71](#_Toc106008310)

[First vaccine dose 71](#_Toc106008311)

[Second vaccine dose 72](#_Toc106008312)

[Blinding 72](#_Toc106008313)

[Unblinding 72](#_Toc106008314)

[Methods: Data collection, management, and analysis 73](#_Toc106008315)

[Data collection methods 73](#_Toc106008316)

[CRF booklet for screening 73](#_Toc106008317)

[CRF booklet for assigned participants 73](#_Toc106008318)

[Diary Card 73](#_Toc106008319)

[Data Management 73](#_Toc106008320)

[Statistical methods 74](#_Toc106008321)

[Safety population 74](#_Toc106008322)

[Immunogenicity population 74](#_Toc106008323)

[Missing data 74](#_Toc106008324)

[Analysis approach 74](#_Toc106008325)

[Analysis plan 74](#_Toc106008326)

[Interim analysis 75](#_Toc106008327)

[Methods: Monitoring 76](#_Toc106008328)

[Harms 76](#_Toc106008329)

[Safety Reporting Guidelines 76](#_Toc106008330)

[Adverse Event (AE) 76](#_Toc106008331)

[Expected Adverse Event 76](#_Toc106008332)

[Serious Adverse Event/Reaction (SAE) 77](#_Toc106008333)

[Suspected Unexpected Serious Adverse Reaction (SUSAR) 78](#_Toc106008334)

[Medically Attended Adverse Event (MAAE) 79](#_Toc106008335)

[How to deal with post-IMP complications 79](#_Toc106008336)

[Ethics and Dissemination 80](#_Toc106008337)

[Research Ethics Approval 80](#_Toc106008338)

[Protocol amendments 80](#_Toc106008339)

[Informed consent 80](#_Toc106008340)

[Confidentiality 81](#_Toc106008341)

[Dissemination policy 81](#_Toc106008342)

[REFERENCES 82](#_Toc106008343)

[Appendix 1, toxicity scoring 83](#_Toc106008344)

[Appendix 2, COVID-19 case definition 91](#_Toc106008345)

[Suspected case 91](#_Toc106008346)

[Probable case 91](#_Toc106008347)

[Confirmed case 91](#_Toc106008348)

[Close contact 92](#_Toc106008349)

[Appendix 3, Randomization sequence list 93](#_Toc106008350)

## List of tables

[Table 1 Specifications of inactivated COVID-19 vaccine 29](#_Toc106008351)

[Table 2 Vaccine quality control tests (during the process and for the final product) 32](#_Toc106008352)

[Table 3 Immunization protocol for BALB/C mice and guinea pigs 33](#_Toc106008353)

[Table 4 Immunization protocol for rabbits and monkeys 35](#_Toc106008354)

[Table 5 Details of receiving the first vaccine dose in Phase I 42](#_Toc106008355)

[Table 6 Responsibilities of participants to reduce their risk of exposure to SARS-CoV-2 within the first month post-vaccination 44](#_Toc106008356)

[Table 7 A complete list of biochemical, hematological and urinalysis tests at different stages of the study 51](#_Toc106008357)

[Table 8 Schedule of expected activities in Phase I - Schedule 1 60](#_Toc106008358)

[Table 9 Schedule of expected activities in Phase I-Schedule 2 66](#_Toc106008359)

[Table 10 Reporting guidelines of expected adverse events 77](#_Toc106008360)

[Table 11 Reporting guidelines of serious adverse events 77](#_Toc106008361)

[Table 12 Reporting guidelines of suspected unexpected adverse reactions 78](#_Toc106008362)

[Table 13 Scoring the severity of local adverse reactions 83](#_Toc106008363)

[Table 14 Scoring the severity of adverse reactions based on vital signs 84](#_Toc106008364)

[Table 15 Scoring the severity of systemic adverse reactions 85](#_Toc106008365)

[Table 16 Scoring the severity of adverse reactions based on laboratory conditions (Serum) 86](#_Toc106008366)

[Table 17 Scoring the severity of adverse reactions based on laboratory conditions (Hematology) 88](#_Toc106008367)

[Table 18 Scoring the severity of adverse reactions based on laboratory conditions (Urine) 90](#_Toc106008368)

## List of figures

[Figure 1 Different design mechanisms in the development and manufacture of COVID-19 vaccine candidates 28](#_Toc106008369)

[Figure 2 The kinetics of drugs in the human body 39](#_Toc106008370)

[Figure 3 Schematic view of E-recruitment 55](#_Toc106008371)

[Figure 4 Participant visit plans in schedule 1, Phase I 59](#_Toc106008372)

[Figure 5 Participant attendance flow diagram in schedule 2, Phase I 65](#_Toc106008373)

## List of abbreviations

| AE | Adverse Event |
| --- | --- |
| ARDS | Acute Respiratory Distress Syndrome |
| COPD | Chronic Obstructive Pulmonary Disease |
| COVID-19 | New Novel Coronavirus |
| CRF | Case Report Form |
| CRO | Contract Research Organization |
| CTA | Clinical Trial Authorization |
| DSMB | Data and Safety Monitoring Board |
| EADR | Expected Adverse Drug Reaction |
| GCP | Good Clinical Practice |
| GMP | Good Manufacturing Practices |
| HIV | Human Immunodeficiency Viruses |
| IgG | Immunoglobulin G |
| IgM | Immunoglobulin M |
| IM | IntraMuscular |
| IMP | Investigational Medicinal Product |
| IRCT | Iranian Registry of Clinical Trials |
| IUD | Intra Uterine Device |
| MAAE | Medically Attended Adverse Events |
| FAKHRAVAC | FAKHRA VACcine |
| N | Nucleocapside protein |
| ProMED | Program for Monitoring Emerging Diseases |
| RBD | Receptor Binding Domain |
| S | Spike glycoprotein |
| SAE | Serious Adverse Events |
| SOP | Standard Operating Procedures |
| SUSAR | Suspected Unexpected Serious Adverse Reaction |
| TCID_50_ | Fifty-percent Tissue Culture Infective Dose |
| WHO | World Health Organization |

# ADMINISTRATIVE INFORMATION moh

## Scientific title

Phase I trial of safety, immunogenicity and dose finding for two strengths of 0.5 × 10^6^ and 2.5 × 10^6^ (TCID_50_) inactivated SARS-CoV-2 vaccine FAKHRAVAC (MIVAC) injected in two schedules of two doses, 2 and 3 weeks apart in healthy adults aged 18-55 years: a randomized, double-blind, placebo-controlled clinical trial

## Public title

Safety, immunogenicity and dose finding for inactivated COVID-19 SARS-CoV-2 vaccine FAKHRAVAC (MIVAC)

## Protocol registration

Once approved by the National Ethical Committee on Research in Medical Sciences, the title will be registered in the Iranian Registry of Clinical Trials (IRCT) certified by the World Health Organization (WHO).

## Protocol version

The Draft Protocol Version 1 was finalized on 12.12.2020

The Draft Protocol Version 2 was finalized on 24.01.2021

The Draft Protocol Version 3 was finalized on 07.03.2021

# Protocol synopsis

## Title

Phase I trial of safety, immunogenicity and dose finding for two strengths of 0.5 × 10^6^ and 2.5 × 10^6^ (TCID_50_) inactivated SARS-CoV-2 vaccine FAKHRAVAC (MIVAC) injected in two schedules of two doses, 2 and 3 weeks apart in healthy adults aged 18-55 years: a randomized, double-blind, placebo-controlled clinical trial

## Introduction

On December 30, 2019, the website of the International Society for Infectious Diseases (ProMED) published a cluster of cases of "pneumonia of unknown cause" from Wuhan, China (1). On January 9, it was discovered that the cause of the new disease was a type of coronavirus. Following the spread of the disease worldwide, Coronavirus disease 2019 (COVID-19) was declared "a global pandemic" on 11 March 2020 by the WHO (2). After publication of the virus genetic sequence on January 10, 2020, research and collaboration on the safety and immunogenicity of various vaccines against the disease quickly followed throughout the world (3).

The vaccine developed by Milad Darou Noor Company is an inactivated vaccine by inoculation of SARS-CoV-2 IR-sb2-01 strain into Vero cell line, followed by culture, harvesting, purification, concentration, inactivation and finally adding aluminum hydroxide adjuvant. After vaccination, the body can produce an immune response and is expected to prevent COVID-19.

Allergy testing and acute toxicity testing in animals completed in the preclinical study. No abnormalities were observed in the monkeys, and current safety assessment results indicate that the vaccine is safe. The results of immunogenicity tests showed that the vaccine has acceptable immunogenicity in different animals. Neutralizing antibodies and specific antibodies are produced on the seventh day after the first vaccination. The next vaccination on day 14 or 21 after the first vaccination will stimulate production of neutralizing antibodies and specific antibodies. According to the current results of safety and immunogenicity studies, the existing inactivated vaccine is in accordance with the requirements of the new regulation of vaccine against COVID-19, and the quality characteristics of the vaccine are controllable, safe and effective.

### Objectives

Dose finding, safety and immunogenicity for inactivated SARS-CoV-2 vaccine (FAKHRAVAC) developed by Milad Darou Noor Company among healthy population

### Objectives in Phase I

1. Dose finding in phases II and III

2.Safety determination, including determining the reactogenicity during the first 3 hours, local and systemic adverse events and abnormal laboratory findings within the first week after each dose of IMP; and SAE, SUSAR and MAAE up to six months after the last IMP dose in placebo group and vaccine groups with two strengths of 0.5 × 10^6^ and 2.5 × 10^6^ (TCID_50_) per dose and two schedules with 2 and 3 week intervals.

3. Determination of immunogenicity, including measuring the serum level of IgG antibodies specific to N and S antigens using ELISA method, determining the level of neutralizing antibodies and determining cell-mediated immunity against SARS-CoV-2 up to six months after the last IMP dose in placebo and vaccine groups with two strengths of 0.5 × 10^6^ and 2.5 × 10^6^ (TCID_50_) per dose and two injection schedules with 2 and 3 week intervals.

## Interventions

Group 1: Receiving two strengths of 0.5× 10^6^ (TCID_50_) FAKHRAVAC vaccine dose intramuscularly in the deltoid muscle 14 days apart

Group 2: Receiving two strengths of 2.5× 10^6^ (TCID_50_) FAKHRAVAC vaccine dose intramuscularly in the deltoid muscle 14 days apart

Group 3: Receiving two strengths of FAKHRAVAC placebo intramuscularly in the deltoid muscle 14 days apart

Group 4: Receiving two strengths of 0.5× 10^6^ (TCID_50_) FAKHRAVAC vaccine dose intramuscularly in the deltoid muscle 21 days apart

Group 5: Receiving two strengths of 2.5× 10^6^ (TCID_50_) FAKHRAVAC vaccine dose intramuscularly in the deltoid muscle 21 days apart

Group 6: Receiving two strengths of FAKHRAVAC placebo intramuscularly in the deltoid muscle 21 days apart

## Outcomes

Four primary outcomes and five secondary outcomes will be determined in the Phase I.

### Primary outcomes in Phase I

1- Abnormal vital signs and anaphylaxis immediately post-IMP

2- Local adverse event within the first week post-IMP

3- Systemic adverse event within the first week post-IMP

4- Abnormal laboratory findings

### Secondary outcomes in Phase I

1. Determining SAE, SUSAR and MAAE up to six months after the last IMP dose

2. Measuring serum level of IgG antibodies specific to N and S antigens using ELISA in the first schedule (0 and 14 days) on days 0, 7, 14, 28, 42, 72 and months 3, 6 and in the second schedule (0 and 21 days) on days 0, 7, 14, 21, 35, 49 and months 3, 6

3. Determining incidence and severity of COVID-19 disease (according to the Ministry of Health) two weeks after receiving the second dose of IMP

4. Determining the level of neutralizing antibody activity in the first schedule on days 0, 14, 28, 42, and months 3, 6 and in the second schedule on days 0, 21, 35, 49 and months 3, 6

5. Determining the level of cell-mediated immune activity in the first schedule on days 0, 14, 28, 42, and months 3, 6 and in the second schedule on days 0, 21, 35, 49 and months 3, 6

## Methodology

### Trial design

This randomized, double-blind, placebo-controlled clinical trial will be conducted on healthy adults. In Phase I, the study will have six groups (placebo and two strengths of 0.5 × 10^6^ and 2.5 × 10^6^ (TCID_50_) inactivated SARS-CoV-2 vaccine FAKHRAVAC (MIVAC) injected in two schedules of two doses 2 and 3 weeks apart). At the baseline, 15 people without blinding (open label) called Sentinel participants will be enrolled in the study.

### Inclusion criteria

The most important items are listed below. Details are given on page 34.

1. Ages 18 to 55 years in Phase I.

2. Body mass index between 18 and 35 kg/m^2^

3. Being healthy based on clinical and laboratory conditions

4. No current or previous history of COVID-19 infection

5. No pregnancy

6. Using safe contraceptive methods

7. Signing informed consent

### Exclusion criteria

The most important items are listed below. See on page 36 for details.

1. Current involvement with any acute or chronic symptomatic disease requiring ongoing internal care or surgical procedure

2. High-risk occupations at risk of COVID-19 infection, including medical staff, occupations with close contact with the client

3. Employment as military service in the subdivisions of the Armed Forces

4. Breastfeeding

5. History of receiving any investigational vaccine within 30 days prior to screening

6. History of receiving blood or any blood product or immunoglobulin within 3 months prior to screening

7. History of long-term use of immunosuppressive drugs or systemic corticosteroids within 4 months prior to screening

8. History of allergic diseases such as angioedema or anaphylaxis

9. History of any allergy to the drug or vaccine

10. History of cancer or chemotherapy and radiotherapy in the last 5 years

11. History of serious psychiatric illnesses

12. History of blood disorders (dyscrasia, coagulation disorders, platelet deficiency or disorder, deficiency of blood factors)

13. History of chronic obstructive pulmonary disease such as asthma, ischemic heart disease definitively diagnosed by a specialist

14. In Phase I, a history of any hypertension being treated by a physician.

15. In Phase I, a history of any diabetes being treated by a physician.

16. Individual and family history of chronic neurological diseases (including seizures and epilepsy)

17. Any history of substance or alcohol abuse in the past 2 years

18. Any defect in the results of hematology or biochemical tests greater than grade 1 performed at the time of screening

19. History of confirmed COVID-19

20. History of acute or chronic hepatitis B and C

21. Taking prophylactic anti tubercular drugs

22. History of syncope during injection or blood drawing

23. Splenectomy for any reason

24. Any close contact with a person with confirmed COVID-19 for a maximum of two weeks before the day of receiving the first vaccine dose

### Withdrawal criteria

The most important items are listed below. Details are given on page 37.

1. Noncompliance to interventions and diagnostic processes

2.Any SAE leading to discontinuation of vaccination as recommended by DSMB/regulator

3. Observing clear biochemical or hematological changes in tests so that continued vaccination may endanger the individual's health at the discretion of the researcher

4. Pregnancy

5. Confirmed COVID-19 before receiving a second dose of IMP

6. Any disease requiring major pharmacological interventions such as intravenous antibiotics or broad-spectrum oral antibiotics, blood or blood products, immunosuppressant or systemic corticosteroids at the discretion of the principal investigator

### Vaccination pause rules

1. Any SAE attributable to the vaccine following IMP injection

2. Severe local or systemic toxicity or based on vital signs (grade 3) in more than 30% in each intervention group within one month after each IMP dose

3. Severe toxicity based on laboratory parameters (grade 3) in more than 30% in each intervention group within one month after each dose of IMP

### Recruitment strategy

Candidates participating in the study, based on the recruitment announced and the schedule set by the research team at two levels through the Internet, telephone and on-site, are examined to meet the inclusion and exclusion criteria.

If the volunteers are eligible, they will be invited to receive an IMP according to a schedule. In the phase I, in total, each person will have at least 9 on-site visits to the study unit.

### Sample size

In the Phase I, 135 participants will enroll in the study.

### Statistical methods

The main approach of analysis in this study will be intended to treat. If protocol deviates for any reason, the outcome information will be collected as much as possible until the end and considered in the analysis stage. Finally, the analysis results with the per-protocol approach will be extracted and compared with the results of the first approach.

After sorting, the collected data will be analyzed by STATA and R 4.3.2 statistical software. First, a descriptive analysis of the variables in the study will be done. Then, to ensure the correctness of random assignment, all variables collected before vaccination (baseline) will be compared in two groups.

The analysis of immunological findings will be done by logarithmic conversion of the measured titers as Geometric Mean Titer. The variable of "Serum level size using geometric mean" and the variable of "Serum positive rate" will be calculated and compared in groups.

### Ethical consideration

Informed written consent will be obtained from all study participants. At the baseline, the necessary explanations about the study conduct, the number of visits for the examination and collection of human samples will be provided to the volunteers. They will be explained that whether or not to participate in the study will not have an unpleasant consequence in terms of receiving health services, and they will be able to leave the study freely whenever they wish.

The informed written consent has been prepared in accordance with the standards of the National Ethics Committee, which will be provided to the participants and their caregivers. They will be given enough time by the research team to study the form and make decisions and answer any possible questions. The participants will be included in the study process if they agree and sign the form. All participants in this study are covered by insurance to compensate for possible vaccine-induced injuries. In addition, participants' transportation costs, side effects recording equipment including medical thermometer and sphygmomanometer, as well as prevention equipment including face mask and alcohol will be provided to participants. Each participant will be given a gift of 2 million Rials for each visit at the end of the study or at the time of leaving the study.

The principles of confidentiality regarding the collection, storage and dissemination of information obtained from volunteers will be observed in this study. To this end, collection forms will be encoded. Only members of the research team will have access to the participants' files and this information will be kept in a safe and secure setting.

All documents will be kept confidential by the sponsor for five years after the end of the study.

# Introduction and background

On December 30, 2019, the website of the International Society for Infectious Diseases (ProMED) published a cluster of cases of "pneumonia of unknown cause" from Wuhan, China. The cluster included four cases of pneumonia with fever (≥38°C) at the beginning of the disease, radiographic evidence consistent with pneumonia or acute respiratory distress syndrome (ARDS), normal or lowered (leukopenia) white blood cell count along with lowered lymphocyte count (lymphopenia). The status of these patients did not change significantly after 3 to 5 days of antibiotic therapy. Initial examinations did not confirm any connection between these individuals. The first patient came from the seafood market (1).

On January 9, it was discovered that the cause of the new disease was a type of coronavirus. The first case of death from a new disease was recorded in China on January 11, 2020. On January 13, the first case of a new disease was discovered in Thailand. Following the spread of the disease worldwide, Coronavirus disease 2019 (COVID-19) was declared "a global pandemic" on 11 March 2020 by the WHO (2). After publication of the virus genetic sequence on January 11, 2020, research and collaboration on the safety and immunogenicity of various vaccines against the disease quickly followed throughout the world (3). On April 11, the WHO published the first report on the start of vaccine production, announcing that 3 vaccines were in the clinical phase and 67 were in the preclinical status (2). Currently, almost everyone agrees that vaccination is the only factor that can prevent the spread of SARS-CoV-2 (4).

As of November 28, 2020, the number of infections worldwide was 62,037,907 and the number of deaths due to the disease was 1,449,895 (5). The first cases of COVID-19 in Iran were on February 19 (6) and by November 27, 2020, 922,397 definite cases with 47,095 deaths were registered in Iran (5).

## Vaccination in COVID-19

### COVID-19 vaccine platforms

A wide range of vaccine platforms can be easily classified into several categories: subunit (protein or polysaccharide), nucleic acid (DNA or RNA), inactivated, vector and live-attenuated (7). ‎ Across all platforms, the Spike antigen is of particular importance in the development of coronavirus vaccine candidates. In this form, the virus structure shows the three-dimensional structure of Spike antigen by determining the position of the receptor binding domain (RBD) of the human cell, the vaccine design pattern, and predicting the time period from the beginning of the manufacturing phase to the end of Phase III clinical trial (6 to 18 months or more).

So-called genetic vaccines such as mRNA, DNA, and nonreplicating viral vectors (like recombinant adenovirus-based vaccines) were the first candidates for which the results of the interim analysis of early-phase clinical trials were published because the platform for these vaccines already existed, and that these vaccines can be produced quickly. Due to the importance of speed of action in responding to pandemics, these types of platforms are better accepted. Genetic vaccination mimics some form of natural infection or live-attenuated vaccines such as those against measles, mumps, and rubella. These vaccines carry the coded directions of the nucleic acid sequence, and direct the body's cells to produce the vaccine protein. An important issue with this type of vaccine is the level of immunity produced by these vaccines (3).

Protein subunit vaccines are a different class of COVID-19 vaccines. These vaccines are made in the form of viral subunits, virus-like particles, or inactivated virus in the laboratory. These vaccines are usually given in combination with adjuvants to increase the response rate and maintain immunity. The hepatitis A vaccine and the inactivated poliovirus vaccine (IPV) or Salk vaccine are examples of inactivated whole-virus vaccines that are licensed (3).

Isolation and inactivation of the virus with formaldehyde is one of the oldest methods of producing viral vaccines. This method has been effective against a wide range of viruses so far. However, there are serious concerns about the use of formaldehyde. Therefore, determination of formaldehyde residue is one of the most important steps in quality control of the final product (7).

Currently, different teams around the world are working on different vaccine platforms, as shown in Figure 1.


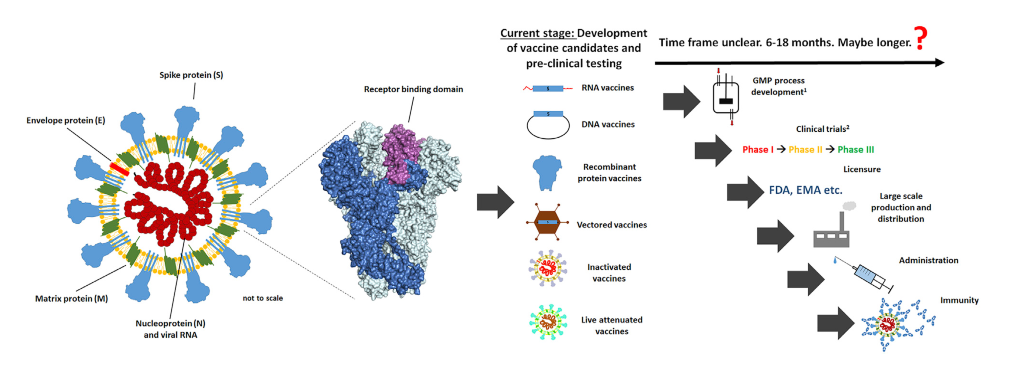


Figure 1 Different design mechanisms in the development and manufacture of COVID-19 vaccine candidates

### Milad inactivated vaccine against COVID-19 (FAKHRAVAC)

Our developed vaccine is an inactivated vaccine by inoculation of SARS-CoV-2 IR-sb2-01 strain into Vero cell line, followed by culture, harvesting, purification, concentration, inactivation and finally adding aluminum hydroxide adjuvant. After vaccination, the body can produce an immune response and is expected to prevent COVID-19.

### Vaccine specifications

Table 1 presents the complete specifications of the inactivated COVID-19 vaccine produced by Milad Darou Noor Company in terms of physical properties, immunological nature, chemical composition and type of packaging.

Table 1 Specifications of inactivated COVID-19 vaccine

| Brand | FAKHRAVAC |
| --- | --- |
| Packing type | USP Type 1- 2R Glass Vial |
| Injection dosage | Single dose |
| Vaccine volume | 0.5ml |
| Injection route | Intramuscular (IM) |
| Appearance | Milky color suspension |

## Preclinical studies

### Introduction

After the outbreak of the disease in the country, several strains of coronavirus (SARS-CoV-2) were successfully isolated from the sample of infected patients. Of all the strains, one strain with high replication potency, the highest induction of immune response and also the resulting antibody with higher potency in viral neutralization was selected as the vaccine seed virus, which was named as SARS-CoV-2 IR-sb2-01 strain. This strain was identified and analyzed based on cytopathology, immunofluorescence, virus replication, morphological examination, complete sequencing, etc. Meanwhile, in cell culture optimization studies, the results related to viral cytopathic characteristics, viral and antigenic titers, immunogenicity and culture stability showed that the strain of interest has good biological properties and can be used to produce vaccines.

Advanced vaccine preparation processes and quality control procedures, summarized below, have been submitted for patent. A specific titer of DMEM high glucose medium was used for virus culture. After 72 to 96 hours of culture, the virus is harvested and then inactivated with formaldehyde (1% by volume) for 24 hours. The inactivated viral solution is collected and subsequently separated by centrifugation and microfiltration with various particle sizes. The virus is then concentrated using a 50-KD ultrafiltration membrane. After inactivation of the formulation with buffer and aluminum hydroxide (alum), filling, packaging and labeling are performed. Inactivation was confirmed by cell culture without any cytopathic effects. The quality control was fulfilled and applied during the process. In addition, the stability of the final products at different temperatures was tested and confirmed. Vero cell protein and DNA residues, and cell culture additives were determined by standard methods. Pre-clinical studies were designed and performed subsequently.

Preclinical studies include all in vivo and in vitro evaluation tests of the vaccine, which are performed prior to the first human vaccine tests. These tests are a prerequisite for clinical studies and include evaluation of product characteristics, validation of immunogenicity and safety test in the animal model.

This evaluation includes a complete set of chemical, physical and biological tests performed on the final product. These tests also include non-process control tests, detection of unwanted factors, determination of compounds added to the process, analysis of process intermediates and lot release tests.

Preclinical studies should be designed to determine whether the potential toxic effects of the vaccine are reasonably harmless for the clinical phase. Important parameters in this design are:

1- Characteristics of the appropriate animal model:

• Sensitivity to pathogens or toxins

• Presentation of an immune response similar to the immune response expected in humans

• Practical administration method

• Analysis the study findings

2- Proper design of schedule and implementation method

3- End point

4- Negative control

### Virus cultivation and inactivation

1- The swab sample of patients with high viral load confirmed by real-time PCR test and preferably with progressive symptoms of fever, chills, myalgia and reduced arterial oxygen saturation is selected and then prepared for culture in the cell substrate.

2- Vero-E6 cells (Sigma C1008) are cultured as monolayers and then infected with a viral sample.

3- The product of virus replication is purified by preparing two serial dilutions and quality evaluations.

4- Pure and confirmed viral seeds are inoculated into the cell substrate, and infected cell supernatants are collected following complete cytopathic effect (CPE) observation. The viral titer is calculated using the Spearman-Karber method.

5- Inactivation of the virus is performed with formaldehyde and confirmed by two consecutive cultures over a period of 10 days.

6- Purification and concentration is done by centrifugation, filtration and ultrafiltration.

7- An appropriate titer for the inactivated virus is formulated with aluminum hydroxide (Al (OH)_3_) and other adjuvants.

8- To evaluate the immunogenicity of the vaccine, Balb/c mice and guinea pigs in different groups are evaluated for the number of injected doses and viral titers.

9- According to the results of the study in mice and guinea pigs, the injection schedule is planned in rabbits and monkeys.

10- Serum samples taken from immunized animals are inactivated at 56°C and accompanied by active virus suspension, followed by addition to the cell substrate after one hour. After 7 days, plaque formation and the virus neutralization process are checked.

11- Body temperature and weight of the animal, any clinical and behavioral manifestations, as well as serum levels of biochemical and inflammatory factors are monitored and recorded on specific days. In addition, histopathological examinations of vital organs of the animal are performed to evaluate the adverse effects of the vaccine.

12- Challenge assay is performed by spraying 10^8^ active viruses through nasal passages on the 14th day after the last injection of Rhesus macaques.

13- For 30 days after the challenge assay, the animal is examined daily and periodically for body temperature, weight, breathing state, food intake, amount of stool, physical condition and response to environmental stimuli. In addition, viral load is estimated by sampling the pharyngeal and anal swabs and real-time PCR testing. The lungs are also examined by CT scan.

### Quality control

In the pre-clinical studies of COVID-19 vaccine, the quality control tests listed in Table 2 were performed to confirm the properties, formulation and immunogenicity, which are described in detail.

Table 2 Vaccine quality control tests (during the process and for the final product)

| No. | Vaccine quality control tests during the process | |
| --- | --- | --- |
| 1 | Sterilization control of cell culture components | |
| 2 | Counting the number of live viruses | |
| 3 | Validation of virus inactivation | |
| 4 | Quantitative and qualitative assessment of the inactivated virus | |
| 5 | Measurement of cell culture residues | Measurement of culture medium protein level |
| 6 |  | Measurement of host cell protein (HCP) level |
| 7 |  | Measurement of host cell DNA level |
| Vaccine quality control tests for the final product | | |
| a. Vaccine formulation quality assessment tests | | |
| 8 | Measurement of aluminum hydroxide concentration | |
| 9 | Measurement of formaldehyde content | |
| 10 | Measurement of sodium chloride concentration | |
| 11 | Measurement of sucrose content (stabilizer) | |
| 12 | Measurement of final vaccine product pH | |
| 13 | Pyrogenicity test | |
| 14 | Sterility test | |
| 15 | Accelerated and long-term stability testing | |
| b. Pharmacodynamic tests in the research and development phase | | |
| 16 | Evaluation of Rhesus monkey susceptibility to SARS-CoV-2 infection | |
| 17 | Assessment of humoral immunity | Determination of antibody titers of immunized animals |
| 18 |  | Serum virus neutralization (SVN) assay |
| 19 | Assessment of cell-mediated immunity | |
| 20 | Vaccine potency test | |
| c. Pharmacokinetic tests in the research and development phase | | |
| 21 | Safety testing | |
| 22 | Toxicity testing | Acute toxicity |
| 23 |  | Chronic toxicity |

### Immunogenicity in laboratory animal models

Immunogenicity is the capacity of a vaccine to induce antibody-dependent immunity or cell-mediated immunity or to create immunological memory. In this project, the immunogenicity of FAKHRAVAC vaccine was tested in Balb/c mice, guinea pigs, rabbits and rhesus monkeys. The antigen content in the injectable formulation of the vaccine for effective immunogenicity depends on the molecular weight and the degree of antigenicity of the antigen. Determining the immunogenicity titer of the antigen is one of the most important steps in developed vaccine-related tests.

### Antigen content determination

Assessments were performed in two steps to determine the minimum antigen content in the final formulation of the FAKHRAVAC vaccine, which induces the highest dose of safe antibody in the animal's blood. In the first step, an initial estimate was made by examining three different doses of the vaccine at three different timing of injection in mice and guinea pigs; Based on the results, injections in rabbits and monkeys were also be scheduled. Three groups of 27 Balb/c mice and three groups of 18 guinea pigs, three different doses of vaccine with TCID_50_ values of (A = 2.5 × 10^6^), (B = 0.5 × 10^6^) and (C = 2.5 × 10^5^) received according to Table 3. The injection was given as IP in Balb/c mice and as IM in guinea pigs. In each group, three animals were injected with placebo. A total of 108 mice and 81 guinea pigs were included in this study.

Table 3 Immunization protocol for BALB/C mice and guinea pigs

| Tested animals | Injection volume (µl) | Group | Injection dose (TCID_50_) | Number of injections | Days of injection | Number of tested animals |
| --- | --- | --- | --- | --- | --- | --- |
| Balb/c mice | 500 | A | 2.5 × 10^6^ | 2 | 0, 14 | 9 |
|  |  |  |  | 2 | 0, 21 | 9 |
|  |  |  |  | 3 | 0, 14, 21 | 9 |
|  | 500 | B | 0.5 × 10^6^ | 2 | 0, 14 | 9 |
|  |  |  |  | 2 | 0, 21 | 9 |
|  |  |  |  | 3 | 0, 14, 21 | 9 |
|  | 500 | C | 2.5 × 10^6^ | 2 | 0, 14 | 9 |
|  |  |  |  | 2 | 0, 21 | 9 |
|  |  |  |  | 3 | 0, 14, 21 | 9 |
| Guinea pigs | 500 | A | 2.5 × 10^6^ | 2 | 0, 14 | 6 |
|  |  |  |  | 2 | 0, 21 | 6 |
|  |  |  |  | 3 | 0, 14, 21 | 6 |
|  | 500 | B | 0.5 × 10^6^ | 2 | 0, 14 | 6 |
|  |  |  |  | 2 | 0, 21 | 6 |
|  |  |  |  | 3 | 0, 14, 21 | 6 |
|  | 500 | C | 2.5 × 10^6^ | 2 | 0, 14 | 6 |
|  |  |  |  | 2 | 0, 21 | 6 |
|  |  |  |  | 3 | 0, 14, 21 | 6 |

Note: There are three placebo-receiving animals in each study group.

### Immunization protocol

After determining the minimum antigen content in Balb/c mice and guinea pigs, immunogenicity in rabbits and monkeys with the specified dose was continued several times at the desired intervals. Based on the results, serum neutralizing antibody titer and stability of antibody titer were evaluated in immunized animal serum samples.

According to the results of the study on mice and guinea pigs, the immunization schedule in rabbits and monkeys was performed according to Table 4. The IM injections were performed in 54 rabbits and 15 monkeys. According to the results obtained in three species of rodents, the highest immunogenic dose of the vaccine (2.5×10^6^) was selected to evaluate the immunogenicity of monkeys.

Table 4 Immunization protocol for rabbits and monkeys

| Tested animals | Injection volume (µl) | Group | Injection dose (TCID_50_) | Number of injections | Days of injection | Number of tested animals |
| --- | --- | --- | --- | --- | --- | --- |
| Rabbits | 500 | A | 2.5 × 10^6^ | 2 | 0, 14 | 6 |
|  |  |  |  | 2 | 0, 21 | 6 |
|  |  |  |  | 3 | 0, 14, 21 | 6 |
|  | 500 | B | 0.5 × 10^6^ | 2 | 0, 14 | 6 |
|  |  |  |  | 2 | 0, 21 | 6 |
|  |  |  |  | 3 | 0, 14, 21 | 6 |
|  | 500 | Placebo | - | 2 | 0, 14 | 6 |
|  |  |  |  | 2 | 0, 21 | 6 |
|  |  |  |  | 3 | 0, 14, 21 | 6 |
| Monkeys | 500 | A | 2.5 × 10^6^ | 2 | 0, 14 | 4 |
|  |  |  |  | 2 | 0, 21 | 4 |
|  |  |  |  | 3 | 0, 14, 21 | 4 |
|  | 500 | Placebo | - | 2 | 0, 14 | 1 |
|  |  |  |  | 2 | 0, 21 | 1 |
|  |  |  |  | 3 | 0, 14, 21 | 1 |

The results of immunogenicity tests in all laboratory animals showed that the vaccine has admirable immunogenicity and can provoke high production of neutralizing and specific antibodies. The results revealed that vaccines can produce specific and neutralizing antibodies through various immunization methods, indicating a good immunogenicity for antigens purified by current processes. As a result, the inactivated vaccine has good immunogenicity in different species of animals. Neutralizing antibodies and specific antibodies can be produced on the seventh day after the first vaccination. Other doses of the vaccine also provoke the production of more neutralizing and specific antibodies on days 14 or 21 after the first vaccination. Various coronaviruses were isolated, and the results of the neutralization experiment showed that the serum of the vaccinated animal could effectively neutralize other isolated strains as well.

Result synopsis

1. Vaccine dose: All vaccinated groups exhibited the best immunogenicity results for 2.5 × 10^6^ viral titers. The results were favorable for a dose of 0.5 × 10^6^. The dose of 0.5 × 10^6^ showed inadequate immunogenicity.

2. Vaccination schedule: In the tested animals, three injections (days 0, 14, 21) showed the best immunogenicity results. The immunogenicity of two injections for the schedule of 0, 21 was reported to be somewhat better than that of 0, 14.

3. Challenge assay and neutralizing antibody test: Viral titer was not significantly different for the doses of 2.5 × 10^6^ and 0.5 × 10^6^. The results were similar for three injections and two injections and did not differ significantly.

Conclusion: Due to the shorter immunogenicity time, two injections are more suitable for clinical trials and both schedules of 0, 14 and 0.21 will be evaluated. The doses of 2.5 × 10^6^ and 0.5 × 10^6^ viral titers are also evaluated in clinical trials. The reason for using the dose of 0.5 × 10^6^ in clinical trials is the possibility of species differences between humans and animals and the need to find the minimum dose for immunogenicity to inoculate the minimum virus into the human body.

### Vaccine safety

This test, which is performed on an animal model, was conducted to prove the safety and tolerability of the vaccine candidate product before the start of clinical trials in humans. In designing this test, species, sex, age and number of animals in each study group, vaccine dose, vaccine administration route and characteristics of control groups should be selected correctly depending on the type of vaccine. This test determines how the vaccine is administered to the host body.

The safety test of FAKHRAVAC vaccine was performed on 20 Balb/c mice (10 males and 10 females). In this experiment, 0.5 ml of the final vaccine product was injected into 20 healthy Balb/c mice weighing 20 to 25 g subcutaneously via a 26G needle. Mortality or any possible apparent abnormal side effects in animals were evaluated within 48 hours.

Based on reference (USP 2003, page 2031, safety test):

• If all of the mice survived and more than one showed no signs of disease or abnormality, the tested sample would be safe.

• If all the mice died or more than one displayed signs of disease or abnormalities, the test should be repeated on 10 mice. If the above result is obtained again, the tested sample will not be Safe.

The results are as follows:

Injections caused no active systemic anaphylaxis. All mice survived and none showed signs of disease or abnormality.

Duration of monitoring the consequences after injection

Immunogenicity is the capacity of a vaccine to induce antibody-dependent immunity or cell-mediated immunity or to create immunological memory. In this project, the immunogenicity of FAKHRAVAC vaccine was tested in Balb/c mice, guinea pigs, rabbits and rhesus monkeys. The antigen content in the injectable formulation of the vaccine for effective immunogenicity depends on the molecular weight and the degree of antigenicity of the antigen.

In the tested animals, on days 7, 14, 21, 28, 35, 49 and 100 after the last injection, the immunostability as well as the consequences of vaccination are monitored and then the animals are sacrificed for histopathological examination.

### Acute toxicity testing in animals

Toxicity testing is performed to evaluate the toxicity of chemicals, biological substances and other substances used in the production of a pharmaceutical product at different intervals.

Test protocol for acute, subacute and chronic toxicities:

In this test, 16 male Balb/c mice weighing 80-120 g were kept in four groups of four (acute toxicity test group - subacute toxicity test group - chronic toxicity test group - control group) under controlled conditions at a temperature of 22±2°C and 12:12 light-dark (LD) cycle with free access to water and food. The experimental groups were injected with three doses of FAKHRAVAC vaccine intramuscularly (one dose more than human use) at similar intervals to humans (0-24-21) and the control group also received sterile normal saline in equal volume. All animals were bled before the first, second and third injections (1 ml with EDTA and 3 ml without EDTA for biochemical tests) for analysis of RBC, WBC, Hematocrit, ALT, AST, ALP, MCV, MCH, MCHC, RDW, GSH, BUN, Creatinine. In addition, mortality, behavioral changes, weight changes, and changes in water and food requirements were assessed for the entire duration of the experiment.

Specific protocols:

1- In the acute toxicity group, blood samples were taken at 24 and 48 hours after the last injection and then the animals were sacrificed by euthanasia for autopsy and pathological sampling. During macroscopic examination to check for lesions in internal organs (including bleeding or change in size and color, etc.), samples were taken from the spleen, liver, kidney, lymph nodes, heart, lungs and brain and sent to the laboratory in 10% formalin solution for slide preparation and pathological examination.

2. In the subacute toxicity group, blood samples were taken at 24 and 48 hours after the last injection and also on days 23 and 45 after the last injection, and then the animals were sacrificed by euthanasia for autopsy and pathological sampling. During macroscopic examination to check for lesions in internal organs (including bleeding or change in size and color, etc.), samples were taken from the spleen, liver, kidney, lymph nodes, heart, lungs and brain and sent to the laboratory in 10% formalin solution for slide preparation and pathological examination.

3. In the chronic toxicity group, blood samples were taken at 24 and 48 hours after the last injection and also on days 23 and 45 after the last injection, and after that monthly until day 180 after the last injection (four blood sampling stages).

Results: During the experiment, no animals died or were not dying and no obvious clinical abnormalities were found. Compared to animals of the negative control group of the same genus during the same period, no statistical differences were observed in body weight and food intake in the vaccine group (p<0.05). Anatomical examination also showed no abnormal changes in the main organs and tissues of each group. Thus, when inactivated vaccines (Vero cells) were injected intramuscularly at two doses at a time in mice, the mice exhibited no abnormal response. Examination of blood biochemical factors also revealed no significant changes before and after vaccination.

### Vaccine efficacy (viral challenge testing)

Fourteen days after the last dose of the vaccine in monkeys, the challenge was performed by spraying 2 ml of active virus (TCID_50_ = 1.5 × 10^8^) in the nasal passages of the animal. In addition, the results of the real-time test for anal and oral specimens as well as their antibody titers were periodically evaluated.

Results: Inactivated vaccine can provoke specific antibodies and neutralizing antibodies in laboratory animals. After receiving coronavirus (TCID_50_ = 1.5 × 10^8^) through nasal spray, virus replication was observed in the lungs of control animals. High-dose viral infections could develop moderate to severe viral pneumonia and acute alveolar injury in controls, whereas there was no lung injury in low-dose and high-dose post-challenge vaccine recipients.

This suggests a protective effect of the inactivated virus vaccine in preventing and reducing virus replication in animals.

### Drug distribution and metabolic pathways in laboratory animals

Figure 2 shows the important issues in the kinetics of drugs in the human body, including the absorption, distribution or storage of drugs, and ultimately drug excretion.

Figure 2 The kinetics of drugs in the human body

The formulated vaccine at a specific dose is converted to a soluble form after inoculation into the body, and is gradually absorbed into the bloodstream through muscle tissue after injection and subsequently activates humoral and cell-mediated immunity.

### Stability studies

Generally, the stability tests to determine the characteristics of the vaccine, including biological activity (such as immunogenicity, antigen content, safety, etc.) of the vaccine before and after formulation, are performed at 1- Recommended storage temperature (actual storage conditions and time) 2- Storage temperature higher than recommended (accelerated tests).

Among the factors influencing the products, in addition to time, only temperature is of special importance. Other factors such as moisture are not required due to the aqueous phase of many formulations as well as the properties of the packaging. In addition to temperature, the effect of light may be tested in the development of some new vaccines, but it is not a common factor needed to evaluate vaccine stability. Due to the type of packaging and transport of the vaccine in light-resistant containers, it does not seem necessary to test the photostability of the vaccine.

In this study, the stability of the final vaccine product was 30 days at the recommended temperature (4°C) and 7 days at 37°C (at least 3 batches were evaluated for each temperature). Before and after the stability study, the factors mentioned in the COVID-19 vaccine stability test form were reviewed and recorded. Factors expected to evaluate the stability of COVID-19 vaccine include physicochemical parameters (sodium chloride concentration, aluminum hydroxide concentration, sucrose concentration and pH), efficacy and sterility. It should be noted that if the efficacy and sterility factors in the batch match after stability tests, physicochemical tests alone can be evidence to confirm other samples of that batch. Finally, the results of the mentioned factors in the samples under stability studies were analyzed and compared with the results of the samples before the stability studies.

The results are as follows:

After concomitant use of the vaccines under stability study, the antigen content did not change, indicating that the antigen content of the inactivated vaccine remained constant for 30 days at 4°C or 7 days at 37°C. The results of physicochemical parameters confirmed the vaccine stability after accelerated tests.

### Pyrogenicity testing

Healthy and adult rabbits were used to measure pyrogenicity. The animals were kept in an environment with constant temperature and humidity as well as away from stressful conditions. The routine animal food and water were used in the animal house for rabbits.

• The original sample was tested using two groups of three rabbits. The solution was injected gently into the marginal vein of the ear of each rabbit in less than four minutes.

• During the 60 minutes before the injection of the sample, the body temperature of each rabbit was recorded twice with an interval of 30 minutes and the average of these temperatures was considered as the basal temperature of the rabbit.

• The difference between the basal temperature and the maximum temperature of each rabbit is considered as the response.

The results were as follows:

The difference between the basal temperature and the maximum temperature of each rabbit was not more than 0.6°C and the total temperature difference of six rabbits was not more than 1.4°C, so the vaccine sample is free of pyrogen.

### Sterility testing

Direct inoculation method was used to perform this test. In this experiment, a certain amount of the product is inoculated directly into the special culture medium, then the culture media are incubated for 14 days. No microorganisms grew on the culture medium, indicating that the vaccine product was completely sterile.

### Summary of pre-clinical studies

Preclinical research developed inactivated vaccine quality preparation and standardization. Allergy testing and acute toxicity testing in animals completed. No abnormalities were observed in the monkeys (the full report will be published later), and current safety assessment results indicate that the vaccine is safe. The results of immunogenicity tests showed that the vaccine has acceptable immunogenicity in different animals. Neutralizing antibodies and specific antibodies are produced on the seventh day after the first vaccination. The next vaccination on day 14 or 21 after the first vaccination can also stimulate overproduction of neutralizing antibodies and specific antibodies. According to the current results of safety and immunogenicity studies, the existing inactivated vaccine is in accordance with the requirements of the new regulation of vaccine against COVID-19, and the quality characteristics of the vaccine are controllable, safe and effective.

## Aim

Safety, immunogenicity and dose finding for inactivated SARS-CoV-2 vaccine FAKHRAVAC (MIVAC) in healthy population

### Phase I objectives

• Dose finding for use in phases II and III

• Determination of safety, including determining the frequency of reactivity during the first 3 hours, local and systemic adverse events and abnormal laboratory findings within the first week after each dose of IMP; and SAE, SUSAR and MAAE up to six months after the last IMP dose in placebo group and vaccine groups with two strengths of 0.5 × 10^6^ and 2.5 × 10^6^ (TCID_50_) per dose and two schedules with 2 and 3 week intervals.

• Determination of immunogenicity, including measuring the serum level of IgG antibodies specific to N and S antigens using ELISA method, determining the level of neutralizing antibodies and determining the level of activity and health of cell-mediated immunity against SARS-CoV-2 up to six months after the last IMP dose in placebo group and vaccine groups with two strengths of 0.5 × 10^6^ and 2.5 × 10^6^ (TCID_50_) per dose and two injection schedules with 2 and 3 week intervals.

## Trial design

### Trial design in Phase I

This randomized, double-blind, placebo-controlled clinical trial will be conducted on healthy adults. In Phase I, the study will be performed as a parallel design (placebo and two strengths of 0.5 × 10^6^ and 2.5 × 10^6^ (TCID_50_) inactivated SARS-CoV-2 vaccine FAKHRAVAC (MIVAC) injected in two schedules of two doses 2 and 3 weeks apart). Recruitment will be in two phases, sentinel phase and main phase. In the first step (sentinel phase), 15 people without blinding (open label) called sentinel participants will be enrolled. Vaccine doses will start from low-to-high for participants in such a way that the vaccine dose of 0.5 × 10^6^ is initially injected into only one person and has a 4-day interval with a dose of 2.5 × 10^6^. The dose of 2.5 × 10^6^ is initially given to only one person. After ensuring the absence of any adverse effects, each vaccine concentration is injected into six other people in two stages two days apart. Thus, a total of 14 people will receive the vaccine and one the placebo. The results of this phase will be reported to the DSMB by the end of Day 8. DSMB will review the initial results and the main phase of vaccinations will begin with the Committee Authorization. In this phase, individuals will enter the study in blocks of 10, each block containing a variable number of different concentrations of vaccine and placebo in two injection schedules with intervals of 2 and 3 weeks. In total, at the end of main phase, 4 groups of 24 people will receive two different concentrations of vaccine in two injection schedules with intervals of 2 and 3 weeks, and 2 groups of 12 people will receive placebo in two injection schedules with intervals of 2 and 3 weeks. Table 5 shows the details of receiving the first vaccine dose in Phase I.

Table 5 number of participants in each block receiving the first vaccine dose in Phase I

|  | | Sentinel blocks | | | | | | | | | Randomized blocks | | | | | | | | | | | |
| --- | --- | --- | --- | --- | --- | --- | --- | --- | --- | --- | --- | --- | --- | --- | --- | --- | --- | --- | --- | --- | --- | --- |
|  |  | Days | | | | | | | | | | | | | | | | | | | | |
|  |  | 0 | 1 | 2 | 3 | 4 | 5 | 6 | 7 | 8 | 9 | 10 | 11 | 12 | 13 | 14 | 15 | 16 | 17 | 18 | 19 | 20 |
| Schedule | IMP* groups |  | | | | | | | | | | | | | | | | | | | | |
| 0-14 | Placebo | 1 |  |  |  |  |  |  |  |  | 1 | 1 | 1 | 1 | 1 | 1 | 1 | 1 | 1 | 1 | 1 | 1 |
|  | Vac. 0.5 × 10^6^ | 1 |  | 3 |  | 3 |  |  |  |  | 4 | 4 | 4 | 4 | 2 | 2 | 2 | 2 |  |  |  |  |
|  | Vac. 2.5 × 10^6^ |  |  |  |  | 1 |  | 3 |  | 3 |  |  |  |  | 2 | 2 | 2 | 2 | 4 | 4 | 4 | 4 |
| 0-21 | Placebo |  |  |  |  |  |  |  |  |  | 1 | 1 | 1 | 1 | 1 | 1 | 1 | 1 | 1 | 1 | 1 | 1 |
|  | Vac. 0.5 × 10^6^ |  |  |  |  |  |  |  |  |  | 4 | 4 | 4 | 4 | 2 | 2 | 2 | 2 |  |  |  |  |
|  | Vac. 2.5 × 10^6^ |  |  |  |  |  |  |  |  |  |  |  |  |  | 2 | 2 | 2 | 2 | 4 | 4 | 4 | 4 |

* Investigational Medicinal Product

# Methods: Participants, interventions and outcomes

## Study setting

The study is performed at Fakhra clinical trial center.

Address: Fakhra clinical trial center, Persian Gulf Hall, Sased Sports Complex, Shahid Fakhrizadeh Street, Sayad Shirazi Highway, Tehran, Iran

## Inclusion and Exclusion (Eligibility) criteria in Phase I

Inclusion and exclusion criteria are set based on expert opinions, experiences of phases I and II of human vaccines, and information extracted from the literature.

### Inclusion criteria in Phase I

1. Iranian citizenship, and residing at a distance of 40-50 km from the study center

2. The ability of participants to study and understand informed consent, preferably educational level of high school and higher

3. Age between 18 and 55 years

4. Body mass index between 18 and 35 kg/m2

5. Being healthy based on clinical and laboratory examinations

6. Temperature less than or equal to 37.2°C sublingually measured by an electronic thermometer 7. Negative IgG and IgM antibody titers against COVID-19 N antigen

8. Negative RT-PCR test for COVID-19

9. Negative ELISA test for anti-HIV IgG

10. Heart rate between 60 and 100

11. Systolic blood pressure between 90 and 140 mm Hg, diastolic blood pressure between 60 and 90 mm Hg

12. Signing informed consent

13. Accepting commitments to reduce the risk of COVID-19 infection in daily life, as shown in Table 6

14. Not pregnant

15. Negative β-hCG pregnancy test on the screening and vaccination days

16. The use of at least one safe method of contraception (condoms, oral contraceptive pills, IUD, Norplant capsule) for women of reproductive age 18 to 49 years

17. Willingness to continue using at least one safe method of contraception (condoms, oral contraceptive pills, IUD, Norplant capsule) for women of reproductive age 18 to 49 years up to three months after the second vaccine dose

18. Participants in the clinical trial should refrain from donating blood or plasma from the time of the first vaccine dose until three months after the second vaccine dose

19. should not participate in another trial during the study period

20. Expressing readiness to remain in the study for the entire study period

21. Married men should use safe methods of contraception up to three months after the second vaccine dose

Table 6 Responsibilities of participants to reduce their risk of exposure to SARS-CoV-2 within the first month post-vaccination

| No. | Description of responsibilities within the first month post-vaccination |
| --- | --- |
| 1 | I do not leave my house as much as possible, except for essentials such as food and medicine and the lack of alternatives. |
| 2 | I use leave from my job or work from home whenever possible. |
| 3 | If I have to leave my house, I do not use public transport at all, and if possible I use a personal vehicle or a taxi. |
| 4 | I avoid attending family gatherings as much as possible, except those family members I am in daily contact with. |
| 5 | I avoid attending public meetings as much as possible. |
| 6 | If I have to leave my house, I will definitely use a face mask. |
| 7 | I wash my hands regularly with soap and water. |
| 8 | In cases where washing with soap and water is impossible and alcohol is available, I disinfect my hands with 70% isopropyl alcohol. |
| 9 | I will notify the research team if a close family member/colleague becomes infected with COVID-19. |

### Exclusion criteria in Phase I

1. Any acute or chronic symptomatic disease requiring ongoing medical or surgical care at the screening day

2. Healthcare workers

3. Breastfeeding

4. History of receiving any vaccine (whether investigational or non-investigational) within 30 days prior to the screening day

5. History of receiving other investigational drugs within 60 days prior to the screening day

6. History of receiving blood or any blood product or immunoglobulin within three months prior to the screening day

7. History of Immunodeficiency disorders (suspected or definite)

8. History of long-term use of immunosuppressive drugs (more than 14 consecutive days) within four months prior to the screening day

9. History of long-term use (more than 14 consecutive days) of systemic corticosteroids (equivalent to 10 mg or more daily prednisolone) or high-dose inhaled steroids (more than 800 µg/day of beclomethasone dipropionate or equivalent) within four months prior to the screening day (excluding topical steroids)

10. History of allergic diseases such as angioedema or anaphylaxis

11. History of any known allergy to drugs or vaccines including aluminum phosphate and albumin

12. History of known allergy to eggs

13. History of autoimmune diseases

14. Chemotherapy or radiotherapy in the last 5 years

15. History of cancer in the last 5 years

16. History of serious psychiatric illnesses

17. History of blood disorders (dyscrasia, coagulation disorders, platelet deficiency or disorder, deficiency of blood factors)

18. History of chronic obstructive pulmonary disease such as asthma diagnosed by a specialist

19. History of ischemic heart disease currently treated by a cardiologist or receiving any cardiac interventions

20. History of hypertension currently treated by a physician

21. History of diabetes currently treated by a physician

22. Congenital anomalies, growth retardation, genetic defects or severe malnutrition

23. Individual or family history of chronic neurological diseases (including seizures and epilepsy)

24. History of thyroid disease or Thyroidectomy

25. Any history of substance or alcohol abuse in the past 2 years

26. Abnormal hematological or biochemical test results at the time of screening

27. History of confirmed COVID-19

28. Acute febrile illness at the time of vaccination

29. History of allergy to acetaminophen

30. History of acute or chronic hepatitis B and C

31. History of pulmonary or extrapulmonary tuberculosis or receiving antituberculotic treatment

32. Receiving tuberculosis prophylaxis

33. History of faint in any encounter with needles or phlebectomy

34. Splenectomy for any reason or history of abnormal spleen function

35. Any close contact with a confirmed COVID-19 case up to two weeks before receiving the first vaccine dose

36. Previous history of diseases such as SARS and MERS

37. Not fit to participate in the trial (the decision is at the discretion of the chief investigator)

38. People who are currently serving their obligatory military service in the Armed Forces

### Withdrawal criteria in Phase I

Individuals can withdraw their consent at any time and for any reason. In this case, they are advised to remain in the study for safety monitoring. On the following occasions, the vaccination process is paused for the candidate:

1- Noncompliance with interventions and diagnostic processes

2- Participants do not attend to receive the second dose of the vaccine within seven days from the scheduled date, despite three telephone follow-up calls at a 24-hours interval

3- Unable to perform laboratory safety assessments: on two occasions with a seven days maximum distance from the specified date, despite three telephone follow-up calls at a 24-hours interval

4- Unable to attend clinical safety on-site visits on two consecutive or three non-consecutive booked sessions, despite three telephone follow-up calls at a 24-hours interval

5- Unable to perform laboratory immunogenicity assessments on two consecutive or three non-consecutive booked sessions, despite three telephone follow-up calls at a 24-hours interval

6- Serious adverse reaction to the previous vaccination

7- History of high fever for three days (oral temperature of ≥39°C) or severe allergic reactions to the previous vaccination

8- Occurrence of SAE or SUSAR leading to DSMB/regulator recommending a pause

9- Occurrence of abnormal biochemical or hematological tests so that the chief investigator advises against vaccination

10- Pregnancy

11- Infection with confirmed COVID-19 in the interval between receiving the first and second doses

12- Experiencing any disease requiring major pharmacological interventions such as intravenous antibiotics or broad-spectrum oral antibiotics, blood or blood products, immunosuppressants, or systemic corticosteroids at the discretion of the chief investigator

13- Use of non-specific immunoglobulins during the study

In all of the above cases, the withdrawal reasons will be recorded in the relevant form in the CRF. Despite not receiving the second dose of vaccine, information about safety and immunogenicity are collected and followed up until the end of the study for these people. The window period for delays in testing and evaluation will be one week in the first two months and a maximum of two weeks after that.

## Interventions

### Group 1: Receiving 0.5× 10^6^ (TCID_50_) vaccine strength at 0-14 schedule

Receiving two intramuscular doses of 0.5×10^6^ (TCID50) strength of the vaccine equivalent to 5 μg/dose at a 14-days interval

### Group 2: Receiving 2.5 × 10^6^ (TCID_50_) vaccine strength at 0-14 schedule

Receiving two intramuscular doses of 2.5×10^6^ (TCID50) strength of the vaccine equivalent to 10 μg/dose at a 14-days interval

### Group 3: Receiving placebo at 0-14 schedule

Receiving two intramuscular doses of a placebo at a 14-days interval

### Group 4: Receiving 0.5× 10^6^ (TCID_50_) vaccine strength at 0-21 schedule

Receiving two intramuscular doses of 0.5×10^6^ (TCID50) strength of the vaccine equivalent to 5 μg/dose at a 21-days interval

### Group 5: Receiving 2.5 × 10^6^ (TCID_50_) vaccine strength at 0-21 schedule

Receiving two intramuscular doses of 2.5×10^6^ (TCID50) strength of the vaccine equivalent to 10 μg/dose at a 21-days interval

### Group 6: Receiving placebo at 0-21 schedule

Receiving two intramuscular doses of a placebo at a 21-days interval

### Storage and preparation

The vaccine and placebo vials are stored at 4 to 8°C. They will be single-dose preparations.

The sealed vials can be stored in the refrigerator of the center for up to 18 months. The temperature inside the refrigerator will be monitored using a data logger and should be kept in the range of 4-8°C. Vaccine consignments should be tried as much as possible to be delivered to the clinical center in small quantities for a maximum of one month.

### Administration route

- The vaccine/placebo is injected into the deltoid muscle.

- Vaccinators should wash their hands with soap and water before injecting.

- The injection site should be disinfected with alcohol prep pads in a circular motion from the center to the outside.

- If the vaccination site is dirty, it should be washed with soap and water.

- The vaccine should be given after the alcohol is completely dry.

- Insulin syringe is used for injection. The vaccine is first drawn into the syringe with a long needle and then injected into the deltoid muscle after replacing the needle.

- The volume of injection is 0.5 cc.

- Both doses of IM injection are best done in one arm.

### Vaccination pause rules

As soon as any of the following are observed, the vaccination process is paused, and a DSMB session is held. Based on the judgment of the committee members, a decision will be made regarding the continuation of the vaccination process.

- Occurrence of any SAE or SUSAR because of the vaccine

- Occurrence of severe local or systemic adverse reaction or abnormal vital signs of grade 3 or higher in more than 30% of individuals in each group within one month after each injection

- Occurrence of abnormal laboratory findings of grade 3 or higher in more than 30% of individuals in each group within one month after each injection

Notwithstanding the above rules, the sponsor can request a DSMB meeting at any time if concerned about the continuation of the trial.

If any of the following conditions are present, the clinical trial should be terminated prematurely:

- If the project sponsor finds that the vaccine is hazardous or that the quality of the research is unacceptable, it may request a complete cessation of the trial.

- If the ethics committee demands the termination of the trial due to violations of ethical principles.

- If the regulatory authorities demand an end to the trial.

##

## Outcomes

## Primary outcomes

### Abnormal vital signs and anaphylactic reactions immediately after vaccination

Number and percentage of people who experience abnormal vital signs in the first three hours after each vaccine dose are recorded based on severity scores according to the table in the Appendix 1. The vital signs, including body temperature, respiratory rate, heart rate, systolic and diastolic blood pressure, are measured before and immediately after vaccination. Then, these measurements will be repeated every hour for at least 3 hours. The measurements will be repeated in the event of any change in the general condition of the subjects.

Anaphylaxis is an acute hypersensitivity disorder that occurs rapidly and within minutes to hours after injections, and usually includes more than one of the following symptoms: erythema, pruritus, urticaria and angioedema, bronchospasm, laryngeal edema, dizziness, hypotension, nausea, dyspnea, wheezing, arrhythmia, cyanosis, vomiting, diarrhea and abdominal pain.

Measurement method: The sublingual temperature is measured using a digital thermometer. Blood pressure will be measured using a digital sphygmomanometer in sitting position.

### Local adverse events within the first week post-vaccination

Number and percentage of patients with local adverse event within the first seven days after each vaccination are recorded based on the complication score, duration and peak intensity according to the table in Appendix 1. These events include pain, tenderness, erythema/redness and swelling/induration.

Measurement method: diary card will be given to patients at the time of vaccination and they are asked to bring them at the next visit. These patients will be contacted daily during these seven days and the research team will ensure that the cards are completed.

Measurement frequency: daily for the first seven days after each vaccination

### Systemic adverse event within the first week post-vaccination

Number and percentage of patients with systemic adverse event within the first seven days after each vaccination are recorded based on the complication score, duration and peak intensity according to the table in Appendix 1. These events include nausea/vomiting, diarrhea, headache, fatigue and myalgia.

Measurement method: diary card will be given to patients at the time of vaccination and they are asked to bring them at the next visit. These patients will be contacted daily during these seven days and the research team will ensure that the cards are completed.

Measurement frequency: daily for the first seven days after each vaccination

### Abnormal laboratory findings

Number and percentage of people who experience abnormal laboratory findings seven days after each vaccination are recorded based on complication score according to the table in Appendix 1. These tests include biochemistry, hematology, and urine parameters, listed in Table 7.

Table 7 A complete list of biochemical, hematological and urinalysis tests at different stages of the study

|  | Screening | 1 wk Post vaccination |
| --- | --- | --- |
| Hemoglobin gm/dL | √ | √ |
| WBC cell/mm3 | √ | √ |
| Lymphocytes cell/mm3 | √ | √ |
| Neutrophils cell/mm3 | √ | √ |
| Eosinophils - cell/mm3 | √ | √ |
| Platelets cell/mm3 | √ | √ |
| PT (prothrombin time) | √ | - |
| PTT (partial thromboplastin time) | √ | - |
| ESR | √ | √ |
| CRP | √ | √ |
| LDH | √ | √ |
| CPK | √ | √ |
| ANA | √ | - |
| TSH | √ | - |
| D-dimer | √ | - |
| Serum ferritin | √ | - |
| Ig electrophoresis | √ | - |
| RT-PCR for SARS-CoV-2 | √* | √ |
| IgM/IgG for SARS-CoV-2 | √ | - |
| HBsAg, HBcAb | √ | - |
| HIV | √ | - |
| HCV | √ | - |
| Sodium, mEq/L | √ | √ |
| Potassium, mEq/L | √ | √ |
| HbA1c | √ | - |
| BS | √ | - |
| BUN mg/dL | √ | √ |
| Creatinine – mg/dL | √ | √ |
| Calcium, mg/dL | √ | - |
| Magnesium, mg/dL | √ | - |
| Phosphorous, mg/dL | √ | - |
| Albumin, g/dL | √ | - |
| Total Protein, g/dL | √ | - |
| Alkaline phosphatase IU/L | √ | √ |
| ALT, AST IU/L | √ | √ |
| Bilirubin (total) | √ | √ |
| Uric Acid mg/dL | √ | - |
| β-hCG (on the day of vac) | √ | - |
| U/A, Urine protein | √ | √ |
| U/A, Urine glucose | √ | √ |
| U/A, RBC | √ | √ |
| Peripheral blood smear | √ | - |

* Twice in two days

## Secondary outcomes

### SAEs, SUSARs, MAAEs, up to six months after last vaccine dose

The number and percentage of SAEs, SUSARs and MAAEs will be counted and recorded up to six months after the last vaccine dose. Participants will be followed monthly.

### Occurrence of COVID-19 disease two weeks after second vaccine dose

The number, percentage and severity of cases of COVID-19 with positive PCR tests occurring 14 days after the second vaccine dose are counted and compared in groups.

### Serum ELISA IgG level for SARS-CoV-2 N and S antigens

The level of specific IgG antibodies for N and S antigens is measured by ELISA in two schedules as follows:

- In 0-14 schedule: on days 0, 7, 14, 28, 42, 72 and months 3, 6

- In 0-21 schedule: on days 0, 7, 14, 21, 35, 49 and months 3, 6

### Neutralizing antibody activity

The humoral immunity based on SARS-CoV-2 neutralizing antibody titer will be evaluated using conventional neutralizing antibody test in two schedules as follows. This test will be done for all the participant on day 0 and two weeks after the second dose. For the remaining time points it was measured in only 20% of participants.

- In 0-14 schedule: on days 0, 14, 28, 42, and months 3, 6

- In 0-21 schedule: on days 0, 21, 35, 49 and months 3, 6

The immunogenicity tests will be performed at the Stem Cell Technology Research Center, Tehran, Iran. Samples of serological tests will be kept at the test site for one year. Neutralizing antibody activity (conventional virus neutralization test) will be done in a BL3 laboratory.

### Cell-mediated immunity

Cell-mediated immunity and safety of the immune response will be measured in two schedules as follows. This test will be done for all the participant on day 0 and two weeks after the second dose. For the remaining time points it was measured in only 20% of participants.

- In 0-14 schedule: on days 0, 14, 28, 42, and months 3, 6

- In 0-21 schedule: on days 0, 21, 35, 49 and months 3, 6

Absolute measurement of lymphocyte cell subpopulations (B, T, NK) and their ratio, measurement of T cell subpopulations (CD3 + CD4 +, CD3 + CD8 +), measurement of TNF-a and interleukins 4, 5, 2, 17, 6, 12, 17A, 17F, 21, 8 and 10.

## Study conduct

The study is performed at Fakhra clinical trial center.

Address: Fakhra clinical trial center, Persian Gulf Hall, Sased Sports Complex, Shahid Fakhrizadeh Street, Sayad Shirazi Highway, Tehran, Iran

### Recruitment Strategy

After approval by the Food and Drug Administration, participants will be recruited to study through a website that will be set up for the study. The necessary information about the study will be uploaded on the website. The participants will fill out the initial screening questionnaire online. The information uploaded by the volunteers is used to identify potentially elligible individuls that will be invited to attend the clinical unit to sign the writen informed consent and undergo formal screening (Figure 3).

Figure 3 Schematic view of E-recruitment

Participant visits the website

Not eligible

Eligible

- Review of medical

- Examination

- experiments

Second recruitment to

receive the vaccine

Positive

Female

beta-hCG

Assign a randomization

Code

(in the absence of acute fever)

Not eligible

Eligible

Not eligible

Recruitment for initial checks

Online screening

Negative

Male

## Screening

Screening will be done in two stages, online and on-site.

### Online screening

Online screening aims to reduce the time cost imposed on candidates, reduce unnecessary trips to the clinical unit, and help plan on-site screening visits for potential study participants. Volunteers will be directed toward a website for registration. If they are willing to participate in the trial, they are asked to complete the relevant questionnaires and get acquainted with the conditions and objectives of the trial. At the end of this phase, the research team reviews inclusion and exclusion criteria based on information provided and requests eligible volunteers by telephone to visit the clinical unit for on-site screening as scheduled.

### On-site screening

Those who have successfully finished the online screening process will be invited to attend the research unit. They should sign a written informed consent and undergo psychological, clinical, and laboratory examinations.

The research team aims to recruit the eligible candidates within less than a week after the laboratory evaluation.

### Obtaining informed consent

Written informed consent will be given to the candidates. The research team will allow candidates to study and make informed decisions by explaining various clauses in the consent form. Participants are then asked to sign the document in two copies and put their fingerprints on the form.

### Psychological assessment

A psychiatrist interview all individuals to assess their mental health and capacity to provide informed consent. The issue of their family consent and support will also be discussed. Eligible participants will enter the next stage.

### Clinical assessment

Clinical assessment will be done by taking a comprehensive medical history and performing a thorough medical examination of all organs (neck, ears, eyes, nose, mouth, lymph nodes, cardiovascular system, lungs, abdomen, limbs, nervous system) and recording vital signs (blood pressure, temperature, respiration rate, and heart rate).

If the initial examination shows mild symptoms due to acute and transient diseases, the examination and tests are postponed to another session for a maximum of two visits.

### Laboratory assessment

List of laboratory tests has be shown in Table 7. The test results will be categorized according to FDO toxicity grading (see Appendix 1, toxicity scoring). Individuals with grade two or high abnormal laboratory findings will be excluded from the study.

Diagnostic and immunological tests will be performed at Farda laboratory and urine and blood espesimens will be taken in the clinical unit.

## Recruitment

For detailed description of recruitment see Trial design on page 33. In all of these people, the pre-injection vital signs are checked and recorded. In women, a negative β-hCG test on the same day is a pre-condition for vaccination. The vital signs will be measured and recorded for up to three hours every hour after vaccination. For sentinel participants accommodation will be provided for the first 24 hours to monitor them closly for posibale adverse events.

## Participant visit plans

In each visit, activities are defined, the details of which will be given below. At the end of each visit, they will be given the necessary and relevant training such as, how to record possible complications using a "diary card" and, how to use an electronic sphygmomanometer, thermometer and ruler. In addition, the contact number of the on-call physician will be provided to the participants.

## Participant visit plans in 0-14 schedule

The participant visit plans in the 0-14 schedule are summarized in Table 8 and Figure 4.

Figure 4 Participant visit plans in 0-14 schedule

Table 8 Expected activities in 0-14 Schedule

|  | Visit plans | | | | | | | | | |
| --- | --- | --- | --- | --- | --- | --- | --- | --- | --- | --- |
| Activities | Screening on day -7 | Day 0 | Day 7 | Day 14 | Day 21 | Day 28 | Day 42 | Day 72 | Month 3 | Month 6 |
| Obtaining informed consent | ☑ |  |  |  |  |  |  |  |  |  |
| Evaluation of inclusion and exclusion criteria | ☑ |  |  |  |  |  |  |  |  |  |
| Random allocation |  | ☑ |  |  |  |  |  |  |  |  |
| Pregnancy test for β-hCG in women | ☑ | ☑ |  | ☑ |  |  |  |  |  |  |
| IMP administration |  | ☑ |  | ☑ |  |  |  |  |  |  |
| Physical examination | ☑ | ☑ |  | ☑ | ☑ | ☑ | ☑ | ☑ | ☑ | ☑ |
| On-site evaluation of complications |  | ☑ | ☑ | ☑ | ☑ | ☑ | ☑ | ☑ | ☑ | ☑ |
| Telephone calls for evaluation of complications |  | ☑ daily | | ☑ daily | |  |  |  | ☑ monthly in the absence of on-site plan | |
| Evaluation of SAE, SUSAR, MAAE cases |  | ☑ Follow up on emergencies | | | | | | | | |
| Laboratory evaluation of safety |  | ☑ |  | ☑ | ☑ | ☑ |  |  |  |  |
| Measurement of serum levels of N/S antigen-specific IgG antibodies |  | ☑ | ☑ | ☑ |  | ☑ | ☑ | ☑ | ☑ | ☑ |
| Evaluation of neutralizing antibody activity |  | ☑ |  | ☑ |  | ☑ | ☑ |  | ☑ | ☑ |
| Evaluation of the safety of immune response |  | ☑ |  | ☑ |  | ☑ | ☑ |  | ☑ | ☑ |

An Interim analysis has been planed for two weeks after second dose

### 0-14 schedule, day -7, screening visit

Activities for this visit are listed below:

• Obtaining informed consent

• Interview by a psychiatrist to assess mental health and capacity to provide informed consent. The issue of their family consent and support will also be discussed.

• Filling out a questionnaire to assess inclusion and exclusion criteria

• Physical examination

• Blood sampling for screening tests

### 0-14 schedule, day 0, visit 1

Activities for this visit are listed below:

• Controlling inclusion and exclusion criteria

• Controlling β-hCG in women

• Receiving dedicated IMP

• Examination of vital signs pre-IMP injection, then hourly for up to three hours post-injection

• Examination of local and systemic symptoms

• Examinations for any possible complications

• Physical examination

• Blood sampling to measure serum levels of specific IgG antibodies

• Providing diary card

• Providing a package containing digital thermometer, digital sphygmomanometer and ruler (to record daily complications)

• Follow-up calls to assess complications for seven days after this visit

### 0-14 schedule, day 7, visit 2

Activities for this visit are listed below:

• Examination of vital signs

• Examination of local and systemic symptoms

• Examinations for any possible complications

• Physical examination

• Blood sampling to measure serum levels of specific IgG antibodies

• Blood and urine sampling for laboratory tests according to the second column of Table 7

### 0-14 schedule, day 14, visit 3

Activities for this visit are listed below:

• Physical examination

• Controlling β-hCG in women

• Blood and urine sampling for laboratory tests according to the third column of Table 7

• Blood sampling to measure serum levels of specific IgG antibodies

• Receiving dedicated IMP

• Examination of vital signs pre-IMP injection, then hourly for up to three hours post-injection

• Examination of local and systemic symptoms

• Examinations for any possible complications

• Providing diary card

• Follow-up calls to assess complications for seven days after this visit

### 0-14 schedule, day 21, visit 4

Activities for this visit are listed below:

• Examination of vital signs

• Examination of local and systemic symptoms

• Examinations for any possible complications

• Clinical examination

• Blood and urine sampling for laboratory tests according to the second column of Table 7

### 0-14 schedule, day 28, visit 5

Activities for this visit are listed below:

• Examination of vital signs

• Examination of systemic symptoms

• Examination for any possible complications

• Physical examination

• Blood sampling to measure serum levels of specific IgG antibodies

• Blood sampling to measure the activity of neutralizing antibodies and cell-mediated immunity

### 0-14 schedule, day 42, visit 6

Activities for this visit are listed below:

• Examination of vital signs

• Examination of systemic symptoms

• Examination for any possible complications

• Clinical examination

• Blood sampling to measure serum levels of specific IgG antibodies

• Blood sampling to measure the activity of neutralizing antibodies and cell-mediated immunity

### 0-14 schedule, day 72, visit 7

Activities for this visit are listed below:

• Examination of vital signs

• Examination of systemic symptoms

• Examination for any possible complications

• Physical examination

• Blood sampling to measure serum levels of specific IgG antibodies

### 0-14 schedule, month 3, visit 8

Activities for this visit are listed below:

• Examination of vital signs

• Examination for any possible complications

• Physical examination

• Blood sampling to measure serum levels of specific IgG antibodies

• Blood sampling to measure the activity of neutralizing antibodies and cell-mediated immunity

### 0-14 schedule, month 6, visit 9

Activities for this visit are listed below:

• Examination of vital signs

• Examination for any possible complications

• Physical examination

• Blood sampling to measure serum levels of specific IgG antibodies

• Blood sampling to measure the activity of neutralizing antibodies and cell-mediated immunity

##

## Participant visit plans in 0-21 schedule

The participant visit plans in the 0-21 schedule are summarized in Table 9 and Figure 5.

Figure 5 Participant attendance flow diagram in 0-21 schedule

Table 9 Expected activities in 0-21 schedule

|  | Visit plans | | | | | | | | | |
| --- | --- | --- | --- | --- | --- | --- | --- | --- | --- | --- |
| Activities | Screening on day -7 | Day 0 | Day 7 | Day 14 | Day 21 | Day 28 | Day 35 | Day 49 | Month 3 | Month 6 |
| Obtaining informed consent | ☑ |  |  |  |  |  |  |  |  |  |
| Evaluation of inclusion and exclusion criteria | ☑ |  |  |  |  |  |  |  |  |  |
| Random allocation |  | ☑ |  |  |  |  |  |  |  |  |
| Pregnancy test for β-hCG in women | ☑ | ☑ |  |  |  | ☑ |  |  |  |  |
| IMP administration |  | ☑ |  |  | ☑ |  |  |  |  |  |
| Physical examination | ☑ | ☑ |  | ☑ | ☑ | ☑ | ☑ | ☑ | ☑ | ☑ |
| On-site evaluation of complications |  | ☑ | ☑ | ☑ | ☑ | ☑ | ☑ | ☑ | ☑ | ☑ |
| Telephone calls for evaluation of complications |  | ☑ daily | |  | ☑ daily | |  |  | ☑ monthly in the absence of on-site plan | |
| Evaluation of SAE, SUSAR, MAAE cases |  | ☑ Follow up on emergencies | | | | | | | | |
| Laboratory evaluation of safety | ☑ |  | ☑ | ☑ | ☑ | ☑ |  |  |  |  |
| Measurement of serum levels of N/S antigen-specific IgG antibodies |  | ☑ | ☑ | ☑ | ☑ |  | ☑ | ☑ | ☑ | ☑ |
| Evaluation of neutralizing antibody activity |  | ☑ |  |  | ☑ |  | ☑ | ☑ | ☑ | ☑ |
| Evaluation of the safety of immune response |  | ☑ |  |  | ☑ |  | ☑ | ☑ | ☑ | ☑ |

An Interim analysis has been planed for two weeks after second dose

### 0-21 schedule, day -7, screening visit

Activities for this visit are listed below:

• Obtaining informed consent

• Psychiatric examination of mental health and capacity for deciding to participate in the study as well as the consent and support of their family

• Filling out a questionnaire about inclusion and exclusion criteria

• Physical examination

• Blood sampling for screening tests

### 0-21 schedule, day 0, visit 1

Activities for this visit are listed below:

• Controlling inclusion and exclusion criteria

• Controlling β-hCG in women

• Receiving dedicated IMP

• Examination of vital signs pre-IMP injection, then hourly for up to three hours post-injection

• Examination of local and systemic symptoms

• Examinations for any possible complications

• Physical examination

• Blood sampling to measure serum levels of specific IgG antibodies

• Providing diary card

• Providing a package containing digital thermometer, digital sphygmomanometer and ruler (to record daily complications)

• Follow-up calls to assess complications for seven days after this visit

### 0-21 schedule, day 7, visit 2

Activities for this visit are listed below:

• Examination of vital signs

• Examination of local and systemic symptoms

• Examinations for any possible complications

• Physical examination

• Blood sampling to measure serum levels of specific IgG antibodies

• Blood and urine sampling for laboratory tests according to the second column of Table 7

### 0-21 schedule, day 14, visit 3

Activities for this visit are listed below:

• Examination of vital signs

• Examination of local and systemic symptoms

• Examinations for any possible complications

• Physical examination

• Blood sampling to measure serum levels of specific IgG antibodies

• Laboratory assessment

### 0-21 schedule, day 21, visit 4

Activities for this visit are listed below:

• Physical examination

• Controlling β-hCG in women

• Blood and urine sampling for laboratory tests according to the third column of Table 7

• Blood sampling to measure serum levels of specific IgG antibodies

• Receiving dedicated IMP

• Examination of vital signs pre-IMP injection, then hourly for up to three hours post-injection

• Examination of local and systemic symptoms

• Examinations for any possible complications

• Providing diary card

• Follow-up calls to assess complications for seven days after this visit

### 0-21 schedule, day 28, visit 5

Activities for this visit are listed below:

• Examination of vital signs

• Examination of local and systemic symptoms

• Examinations for any possible complications

• Physical examination

• Blood and urine sampling for laboratory tests according to the second column of Table 7

### 0-21 schedule, day 35, visit 6

Activities for this visit are listed below:

• Examination of vital signs

• Examination of systemic symptoms

• Examination for any possible complications

• Clinical examination

• Blood sampling to measure serum levels of specific IgG antibodies

• Blood sampling to measure the activity of neutralizing antibodies and cell-mediated immunity

### 0-21 schedule, day 49, visit 7

Activities for this visit are listed below:

• Examination of vital signs

• Examination of systemic symptoms

• Examination for any possible complications

• Physical examination

• Blood sampling to measure serum levels of specific IgG antibodies

• Blood sampling to measure the activity of neutralizing antibodies and cell-mediated immunity

### 0-21 schedule, month 3, visit 8

Activities for this visit are listed below:

• Examination of vital signs

• Examination for any possible complications

• Physical examination

• Blood sampling to measure serum levels of specific IgG antibodies

• Blood sampling to measure the activity of neutralizing antibodies and cell-mediated immunity

### 0-21 schedule, month 6, visit 9

Activities for this visit are listed below:

• Examination of vital signs

• Examination for any possible complications

• Physical examination

• Blood sampling to measure serum levels of specific IgG antibodies

• Blood sampling to measure the activity of neutralizing antibodies and cell-mediated immunity

## Sample size

The sample size in this study is estimated based on practical considerations and expert opinion. In this study, there are five different groups of 24 receiving IMP (one of the groups recieved placebo, which is divided into two parts according to the 0-14 and 0-21 schedules). In addition, 15 Sentinel participants were included in the study. Therefore, the sample size in Phase I of the study will be 135 people in total.

Those who leave the study before the first IMP injection will be replaced by eligible individuals. People who leave the study after receiving one or two doses of IMP will be encouraged to collaborate with the safety follow up.

# Methods: Assignment of interventions

## Random Allocation

This study utilized the permuted block randomization method in which the order of assigning participants to intervention groups was random.

According to previous explanations in the protocol design section, researchers will not be blind to the type of vaccine received in the initial 15 patients. The participants in the study on the same day will be considered a block. Because we will start the study with two participants on day zero, our block size will be two on day zero, but the order of IMP administration will be determined randomly. Also, this rule will be maintained in the coming days of administration.

Excel and rand() function software will be used to prepare random arrangements inside each block. After determining the type of intervention for each participant, a non-repeating four-digit number is assigned to the individual. This number is considered the randomization code of the participant, and the person will be identified with this number until the end of the study. This number will be displayed on all pages of the participants' data collection forms.

The study epidemiologist will create and keep a chain of 135 codes for use in Phase I of the study. It will be used for unblinding if required at the principal investigator's discretion.

## Concealment

A dedicated four-digit numeric code will be assigned to each intervention type in the random sequnce via study software. These codes has also been printed on the IMP vials and each participant will receive the content of the vial with the same code assigned.

##

## Blinding

This study will use a placebo, which will be the adjuvant used in the vaccine without the vaccine being added. All except the study epidemiologist responsible for creating random codes, will be blind to the type of IMP assigned to the participant.

### Unblinding

Unblinding can be done if necessary under the following situations:

1- Occurrence of severe adverse events (SAEs)

2- Occurrence of abnormal biochemical or hematological test results with toxicity grade 3 or higher

Unblindig will be done after consulting the principal investigator. The reason for unblinding will be recorded in the CRF.

# Methods: Data collection, management, and analysis

## Data collection methods

Data will be collected both through electronic and paper Case Report Forms (CRFs). The content in the two will be cross checked for any discrepancy at the end of working day.

### CRF booklet for screening

This section of the booklet will include the forms required to screen candidates. Only eligibale individuals will enter the randomization phase. Having seprate screening booklet will help with reducing paper waste.

### CRF booklet for assigned participants

This section of the booklet will contain the forms required to collect the information throughout the study.

### Diary Card

This card has been designed to collect information on local and systemic side effects and vital signes in the seven-days after each placebo/vaccine dose. Participants should bering the card with them in their next visit to the study center.

## Data Management

The data will be recorded in paper CRFs as well as electronic CRFs placed in a special software developed for this study. The forms will be kept in locked cupboards and, will be made available only to authorized study personnel. The access to the content of the forms in the software will be managed through username and password.

## Statistical methods

### Safety population

All the participants receiving at least one dose of IMP will be considered as the safety population.

### Immunogenicity population

Efficacy in this study is interpreted as immunogenicity, and therefore all recipients of two doses of vaccine or placebo with at least one available serum sample for immunogenicity assessment at the appropriate time and without serious protocol deviation will be considered as immunogenicity population.

### Missing data

No imputation will be performed on missing data.

### Analysis approach

The main analysis approach in this study will be intention to treat (ITT). If the protocol deviation occurs for any reason, the outcome information will be collected as far as possible and considered in the analysis phase. The per-protocol approach will also be used and the results will be compared with the first approach.

### Analysis plan

First, the variables in the study will be analyzed descriptively. To ensure the accuracy of random allocation, all baseline variables will be compared in the Phase I within five groups.

The collected data, after sorting, will be analyzed by STATA statistical software. Statistical tests will be analyzed considering the type I error of α = 0.05. The data obtained from the safety and immunogenicity tests will first be analyzed descriptively and then be compared with placebo in two schedules and two doses of vaccine in each schedule. The ratios will be compared using the Chi-square test and, if necessary, the Fisher’s Exact test. Immunological findings will be analyzed by logarithmically converting the measured titers to Geometric Mean Titer. Continuous quantitative data with normal distribution in the two groups will be compared by the independent t-test. Ordinal and non-normal quantitative data will be compared by non-parametric Mann-Whitney U test.

### Interim analysis

Due to the national status of COVID-19 and the need for faster community access to an effective vaccine, the interim analysis will be performed 14 days after receiving the second vaccine dose in all Phase I participants. Based on the results of this analysis, a decision will be made regarding the authorization request for the start of the second phase from the Food and Drug Administration.

In this study, the basis for the transition to the second phase is the decisions made by the members of the DSMB and the Food and Drug Administration. The decision will be based on Phase I evidence of safety and immunogenicity on day 28 in the first schedule or day 35 in the second schedule (two weeks after the second vaccine dose). If none of the study pause conditions occur and the neutralizing antibody response occurs in more than 50% of participants, a request to start phase II will be sent to the FDA after DSMB approval.

The criteria for selecting the final antigen concentration for the transition to phase II will be the concentration and schedule, among two concentrations and two schedules, that have the lowest incidence rate and lowest severity of side effects in general, especially in serious side effects and have higher immunogenicity, especially in inducing cell-mediated immunity and neutralizing antibody titers.

# Methods: Monitoring

## Harms

Monitoring of adverse harms is of particular importance in this study; therefore, the FDA guidelines for timely reporting of these harms to the Ethics Committee and the FDA will be strictly followed as follows.

## Safety Reporting Guidelines

These guidelines have been prepared and published by the Food and Drug Administration (9) and the sections related to this study are summarized below.

### Adverse Event (AE)

Adverse Event (AE) refers to any adverse medical events that occur to participants at the same time as participating in this clinical trial. In this case, the occurrence of the event does not necessarily have a causal relationship with the treatment protocols used in this study.

All adverse events will be recorded and discussed in the DSMB for a causal relationship with the vaccine used in the study.

The following information will be collected for each adverse event in CRF.

- Title/Event Description

- Start date

- End date

- Event Intensity

- Treatments performed

- Result of the performed treatments

- Importance of the event

- Measures regarding the drug under study

### Expected Adverse Event

Expected Adverse Event refers to an event that has been observed before following the vaccine use and recorded in the vaccine documentation such as investigator brochure. All expected adverse events will be recorded and discussed in the DSMB for a causal relationship with the IMP used in the study.

If an expected adverse event cause participants to drop out of the study or have a higher prevalence than expected, it should be reported to the Ethics Committee and Sponsor (Milad Darou Noor Company).

Table 10 Reporting guidelines for expected adverse events with a prevalence higher than expected

| Actor | Time of reporting | Action |
| --- | --- | --- |
| Investigator | Once the higher than expected prevalence is ascertained, patient withdrawn because of the adverse event | Reporting to the ethics committee and the sponsor |
| Sponsor | Within maximum 15 days following reciept of notification from the investigator | Reporting to the regulatory authorities (Food and Drug Organization) |

### Serious Adverse Event/Reaction (SAE)

Serious Adverse Event/Reaction (SAE) refers to an event that occurs following vaccination and is life threatening or results in death, hospitalization, prolonged hospital stay, permanent or significant disability, or congenital anomaly. All the treatment modalities undertaken to manage SAEs should also be regarded as an SAE. (10).

Table 11 Reporting guidelines for serious adverse events

| Event | Time of reporting | Action |
| --- | --- | --- |
| Death or life threatening SAE | Immediate, within maximum 24 hours after the investigator becomes aware of the occurrence of SAE (via fax, e-mail, etc.) | Reporting by investigator to the ethics committee and the sponsor |
| SAE not leading to death; not life threatening | Immediate, within maximum 7 days after the investigator becomes aware of the occurrence of SAE | Reporting by investigator to the ethics committee and the sponsor |
| All SAEs | Supplementary report should be sent to regulatory authorities within 15 days after the sponsor becomes aware of the occurrence of SAE | Reporting to the regulatory authorities (Food and Drug Organization) by the sponsor |

### Suspected Unexpected Serious Adverse Reaction (SUSAR)

The SUSAR refers to a serious adverse event that is unexpected, and its characteristics, features, and severity are not addressed in the vaccine documentation and the investigator brochure.

Table 12 Reporting guidelines for suspected unexpected serious adverse reactions

| Event | Time of reporting | Action |
| --- | --- | --- |
| Death or life threatening SUSAR | As soon as possible, within maximum 7 days after the sponsor becomes aware of the occurrence of SUSAR  Supplementary report should be sent to regulatory authorities within 15 days after the sponsor becomes aware of the occurrence of SUSAR | Reporting by the sponsor to the ethics committee and the regulatory authorities (Food and Drug Organization) |
| SUSAR not leading to death; not life threatening | As soon as possible, within maximum 15 days after the sponsor becomes aware of the occurrence of SUSAR  Supplementary report should be sent to regulatory authorities as soon as possible once the report is ready | Reporting by the sponsor to the ethics committee and the regulatory authorities (Food and Drug Organization) |

If the SUSAR is leading to death or is life threatening, the sponsor will report the event to the Ethics Committee and the regulatory authorities (Food and Drug Organization) as soon as possible or within seven days of being notified using the relevant forms.

If the SUSAR does not lead to death or is not life threatening, the maximum reporting time by the sponsor to the Ethics Committee and the regulatory authorities (Food and Drug Organization) could be extended to 15 days.

### Medically Attended Adverse Event (MAAE)

The MAAE refers to events that force participants to seek medical attention, which are observed in all grade 4 adverse events in FDO toxicity grading (see Appendix 1, toxicity scoring).

If the study follow up team becomes aware of an MAAE (via daily or monthly telephone follow-up calls, or volunteer self-report during clinical visits), they will collect additional information by contacting the participant or the medical unit. All the information will be recorded it in the CRF, and reported back to the PI.

## Ethics and Dissemination

### Research Ethics Approval

Ethical approval (Reference number: IR. NREC.1399.006) was obtained for this study in accordance with the national guidelines from the National Ethics Committee on February 28, 2021.

### Protocol amendments

If amendments are needed in sections of the protocol that affect the potential benefit of the volunteers in the study or their safety, there will be a need to seek re-approval from the regulatory authorities (Food and Drug Organization) and the National Ethics Committee. These sections mainly include objectives, study design, study population, sample size, and intervention. Minor changes to the study, which do not affect the study as a whole, will not require re-approval by the National Ethics Committee.

### Informed consent

Informed consent will be obtained from all participants in this study. At the first arrival to the research center, a member of the study team will provide the necessary explanations to the volunteers regarding study conduct, number of visits, and number of specimens collected during the study. Participants will be informed that they can leave the study whenever they want and their questions will be answered.

The written informed consent has been prepared in accordance with the standards of the National Ethics Committee, which will be provided to the participants. They will be given enough time by the research team to study the form and make decisions on whether or not thay want to join the study. Any potential questions will be answered. The participants will be included in the study if they sign the form.

### Confidentiality

The principles of confidentiality regarding the collection, storage and dissemination of information obtained from volunteers will be observed in this study. To this end, the codes assigned to the participants will be written on the data collection forms instead of their names. Only members of the research team will have access to the participants' files and this information will be kept in a safe and secure place.

All documents will be kept confidential by the sponsor for five years after the end of the study.

### Dissemination policy

The results of this study will be reported to the Food and Drug Administration of Iran. In addition, the results will be published as articles in reputable scientific journals in the relevant fields after obtaining the approval of the sponsor.

Parts of the protocol, which do not contain the confidential information of the sponsor and researchers, will be made available to the public along with the results after obtaining their written approval.

# REFERENCES

1. ProMED. UNDIAGNOSED PNEUMONIA - CHINA (HUBEI): REQUEST FOR INFORMATION: ProMED; 2019 [cited 2020 11/1/2020]. Available from: https://promedmail.org/promed-post/?id=6864153%20#COVID19.

2. World Health Organization. Timeline: WHO's COVID-19 response: WHO; 2020 [cited 2020 11/1/2020]. Available from: https://[www.who.int/emergencies/diseases/novel-coronavirus-2019/interactive-timeline](http://www.who.int/emergencies/diseases/novel-coronavirus-2019/interactive-timeline).

3. Mulligan MJ. An Inactivated Virus Candidate Vaccine to Prevent COVID-19. JAMA. 2020;324(10):943-5.

4. Isakova-Sivak I, Rudenko L. A promising inactivated whole-virion SARS-CoV-2 vaccine. The Lancet Infectious Diseases.

5. WHO. Reported Cases and Deaths by Country, Territory, or Conveyance 2020 [cited 2020 11/28/2020]. Available from: https://[www.worldometers.info/coronavirus/#countries](http://www.worldometers.info/coronavirus/#countries).

6. Organization WH. Coronavirus disease 2019 (COVID-19): situation report, 31. 2020.

7. Tregoning JS, Brown ES, Cheeseman HM, Flight KE, Higham SL, Lemm N-M, et al. Vaccines for COVID-19. Clinical & Experimental Immunology. 2020;202(2):92-162

8. Derscheid RJ, Gallup JM, Knudson CJ, Varga SM, Grosz DD, van Geelen A, et al. Effects of formalin-inactivated respiratory syncytial virus (FI-RSV) in the perinatal lamb model of RSV. PloS one. 2013;8(12):e81472.

9. Food and Drug Organization. Guidelines for Safety Reporting During Conduct of Clinical Trials. In: Clinical Trial Committee, editor. 2018.

10. Organization WH. Global manual on surveillance of adverse events following immunization. 2014.

11. Food U, Administration D. Guidance for industry: toxicity grading scale for healthy adult and adolescent volunteers enrolled in preventive vaccine clinical trials. Food and Drug Administration, US Department of Health and Human Services. 2007.

## Appendix 1, toxicity scoring

The toxicity severity will be scored according to the guidelines of the United States Food and Drug Administration (11). This scoring is provided for use in adults and adolescents.

Table 13 Scoring the severity of local adverse reactions

| Local Reaction to  Injectable Product | Mild (Grade 1) | Moderate(Grade 2) | Severe (Grade 3) | Potentially Life  Threatening  (Grade 4) |
| --- | --- | --- | --- | --- |
| Pain | Does not interfere  with activity | Repeated use of non-narcotic pain reliever > 24 hours or interferes with activity | Any use of narcotic pain reliever or prevents daily  activity | Emergency room  (ER) visit or  hospitalization |
| Tenderness | Mild discomfort to touch | Discomfort with movement | Significant  discomfort at rest | ER visit or  hospitalization |
| Erythema/Redness * | 2.5 – 5 cm | 5.1 – 10 cm | > 10 cm | Necrosis or  exfoliative dermatitis |
| Induration/Swelling ** | 2.5 – 5 cm and does not interfere  with activity | 5.1 – 10 cm or  interferes with  activity | > 10 cm or  prevents daily  activity | Necrosis |

* In addition to grading the measured local reaction at the greatest single diameter, the measurement should be recorded as a continuous variable. ** Induration/Swelling should be evaluated and graded using the functional scale as well as the actual measurement.

Table 14 Scoring the severity of adverse reactions based on vital signs

| Vital Signs * | Mild (Grade 1) | Moderate (Grade 2) | Severe (Grade 3) | Potentially Life Threatening  (Grade 4) |
| --- | --- | --- | --- | --- |
| Fever (°C) ** | 38.0 – 38.4 | 38.5 – 38.9 | 39.0 – 40 | > 40 |
| Tachycardia - beats per  minute | 101 – 115 | 116 – 130 | > 130 | ER visit or hospitalization for arrhythmia |
| Bradycardia - beats per  minute*** | 50 – 54 | 45 – 49 | < 45 | ER visit or hospitalization for arrhythmia |
| Hypertension (systolic) -  mm Hg | 141 – 150 | 151 – 155 | > 155 | ER visit or hospitalization for malignant hypertension |
| Hypertension (diastolic) - mm Hg | 91 – 95 | 96 – 100 | > 100 | ER visit or hospitalization for  malignant hypertension |
| Hypotension (systolic) –  mm Hg | 85 – 89 | 80 – 84 | < 80 | ER visit or hospitalization for  hypotensive shock |
| Respiratory Rate – breaths per minute | 17 – 20 | 21 – 25 | > 25 | Intubation |

* Subject should be at rest for all vital sign measurements. ** Oral temperature; no recent hot or cold beverages or smoking. *** When resting heart rate is between 60 – 100 beats per minute. Use clinical judgement when characterizing bradycardia among some healthy subject populations, for example, conditioned athletes.

Table 15 Scoring the severity of systemic adverse reactions

| Systemic (General) | Mild (Grade 1) | Moderate(Grade 2) | Severe (Grade 3) | Potentially Life  Threatening  (Grade 4) |
| --- | --- | --- | --- | --- |
| Nausea/vomiting | No interference  with activity or 1 – 2  episodes/24 hours | Some interference  with activity or > 2  episodes/24 hours | Prevents daily  activity, requires  outpatient IV  hydration | ER visit or  hospitalization for  hypotensive shock |
| Diarrhea | 2 – 3 loose stools or  < 400 gms/24 hours | 4 – 5 stools or  400 – 800 gms/24  hours | 6 or more watery  stools or > 800 gms/24 hours  or requires outpatient IV  hydration | ER visit or  hospitalization |
| Headache | No interference with  activity | Repeated use of non-  narcotic pain  reliever > 24 hours or  some interference  with activity | Significant; any  use of narcotic  pain reliever or  prevents daily  activity | ER visit or  hospitalization |
| Fatigue | No interference with  activity | Some interference  with activity | Significant;  prevents daily  activity | ER visit or  hospitalization |
| Myalgia | No interference with  activity | Some interference  with activity | Significant;  prevents daily  activity | ER visit or  hospitalization |
| Illness or clinical adverse event (as defined according to applicable  regulations) | No interference with  activity | Some interference  with activity not  requiring medical  intervention | Prevents daily  activity and  requires medical  intervention | ER visit or  hospitalization |

Table 16 Scoring the severity of adverse reactions based on laboratory conditions (Serum)

| Serum * | Mild (Grade 1) | Moderate (Grade 2) | Severe  (Grade 3) | Potentially Life  Threatening  (Grade 4)** |
| --- | --- | --- | --- | --- |
| Sodium – Hyponatremia mEq/L | 132 – 134 | 130 – 131 | 125 – 129 | < 125 |
| Sodium – Hypernatremia mEq/L | 144 – 145 | 146 – 147 | 148 – 150 | > 150 |
| Potassium – Hyperkalemia mEq/L | 5.1 – 5.2 | 5.3 – 5.4 | 5.5 – 5.6 | > 5.6 |
| Potassium – Hypokalemia mEq/L | 3.5 – 3.6 | 3.3 – 3.4 | 3.1 – 3.2 | < 3.1 |
| Glucose – Hypoglycemia mg/dL | 65 – 69 | 55 – 64 | 45 – 54 | < 45 |
| Glucose – Hyperglycemia  Fasting – mg/dL  Random – mg/dL | 100 – 110  110 – 125 | 111 – 125  126 – 200 | >125  >200 | Insulin requirements or hyperosmolar coma |
| Blood Urea Nitrogen  BUN mg/dL | 23 – 26 | 27 – 31 | > 31 | Requires dialysis |
| Creatinine – mg/dL | 1.5 – 1.7 | 1.8 – 2.0 | 2.1 – 2.5 | > 2.5 or requires dialysis |
| Calcium – hypocalcemia mg/dL | 8.0 – 8.4 | 7.5 – 7.9 | 7.0 – 7.4 | < 7.0 |
| Calcium – hypercalcemia mg/dL | 10.5 – 11.0 | 11.1 – 11.5 | 11.6 – 12.0 | > 12.0 |
| Magnesium – hypomagnesemia mg/dL | 1.3 – 1.5 | 1.1 – 1.2 | 0.9 – 1.0 | < 0.9 |
| Phosphorous – hypophosphatemia mg/dL | 2.3 – 2.5 | 2.0 – 2.2 | 1.6 – 1.9 | < 1.6 |
| CPK – mg/dL | 1.25 – 1.5 x ULN*** | 1.6 – 3.0 x ULN | 3.1 –10 x ULN | > 10 x ULN |
| Albumin – Hypoalbuminemia g/dL | 2.8 – 3.1 | 2.5 – 2.7 | < 2.5 | -- |
| Total Protein – Hypoproteinemia g/dL | 5.5 – 6.0 | 5.0 – 5.4 | < 5.0 | -- |
| Alkaline phosphate –  increase by factor | 1.1 – 2.0 x ULN | 2.1 – 3.0 x ULN | 3.1 – 10 x ULN | > 10 x ULN |
| Liver Function Tests –ALT, AST  increase by factor | 1.1 – 2.5 x ULN | 2.6 – 5.0 x ULN | 5.1 – 10 x ULN | > 10 x ULN |
| Bilirubin – when accompanied  by any increase in Liver Function Test  increase by factor | 1.1 – 1.25 x ULN | 1.26 – 1.5 x ULN | 1.51 – 1.75 x ULN | > 1.75 x ULN |
| Bilirubin – when Liver Function Test is normal; increase by factor | 1.1 – 1.5 x ULN | 1.6 – 2.0 x ULN | 2.0 – 3.0 x ULN | > 3.0 x ULN |
| Cholesterol | 201 – 210 | 211 – 225 | > 226 | --- |
| Pancreatic enzymes – amylase, lipase | 1.1 – 1.5 x ULN | 1.6 – 2.0 x ULN | 2.1 – 5.0 x ULN | > 5.0 x ULN |

* The laboratory values provided in the tables serve as guidelines and are dependent upon institutional normal parameters. Institutional normal reference ranges should be provided to demonstrate that they are appropriate. ** The clinical signs or symptoms associated with laboratory abnormalities might result in characterization of the laboratory abnormalities as Potentially Life Threatening (Grade 4). For example, a low sodium value that falls within a grade 3 parameter (125-129 mE/L) should be recorded as a grade 4 hyponatremia event if the subject had a new seizure associated with the low sodium value. ***ULN” is the upper limit of the normal range.

Table 17 Scoring the severity of adverse reactions based on laboratory conditions (Hematology)

| Hematology * | Mild (Grade 1) | Moderate  (Grade 2) | Severe (Grade 3) | Potentially Life  Threatening (Grade 4) |
| --- | --- | --- | --- | --- |
| Hemoglobin (Female) - gm/dL | 11.0 – 12.0 | 9.5 – 10.9 | 8.0 – 9.4 | < 8.0 |
| Hemoglobin (Female)  change from baseline value - gm/dL | Any decrease – 1.5 | 1.6 – 2.0 | 2.1 – 5.0 | > 5.0 |
| Hemoglobin (Male) - gm/dL | 12.5 – 13.5 | 10.5 – 12.4 | 8.5 – 10.4 | < 8.5 |
| Hemoglobin (Male)  change from baseline value – gm/dL | Any decrease – 1.5 | 1.6 – 2.0 | 2.1 – 5.0 | > 5.0 |
| WBC Increase - cell/mm^3^ | 10,800 – 15,000 | 15,001 – 20,000 | 20,001 – 25, 000 | > 25,000 |
| WBC Decrease - cell/mm^3^ | 2,500 – 3,500 | 1,500 – 2,499 | 1,000 – 1,499 | < 1,000 |
| Lymphocytes Decrease - cell/mm^3^ | 750 – 1,000 | 500 – 749 | 250 – 499 | < 250 |
| Neutrophils Decrease - cell/mm^3^ | 1,500 – 2,000 | 1,000 – 1,499 | 500 – 999 | < 500 |
| Eosinophils - cell/mm^3^ | 650 – 1500 | 1501 - 5000 | > 5000 | Hypereosinophilic |
| Platelets Decreased - cell/mm^3^ | 125,000 – 140,000 | 100,000 – 124,000 | 25,000 – 99,000 | < 25,000 |
| PT – increase by factor  (prothrombin time) | 1.0 – 1.10 x ULN** | 1.11 – 1.20 x ULN | 1.21 – 1.25 x ULN | > 1.25 ULN |
| PTT – increase by factor  (partial thromboplastin time) | 1.0 – 1.2 x ULN | 1.21 – 1.4 x ULN | 1.41 – 1.5 x ULN | > 1.5 x ULN |
| Fibrinogen increase - mg/dL | 400 – 500 | 501 – 600 | > 600 | -- |
| Fibrinogen decrease - mg/dL | 150 – 200 | 125 – 149 | 100 – 124 | < 100 or associated  with gross bleeding  or (DIC) |

Table 18 Scoring the severity of adverse reactions based on laboratory conditions (Urine)

| Urine * | Mild (Grade 1) | Moderate  (Grade 2) | Severe (Grade 3) | Potentially Life Threatening  (Grade 4) |
| --- | --- | --- | --- | --- |
| Protein | Trace | 1+ | 2+ | Hospitalization or  dialysis |
| Glucose | Trace | 1+ | 2+ | Hospitalization for  hyperglycemia |
| Blood (microscopic) –  red blood cells per  high power field (rbc/hpf) | 1 - 10 | 11 – 50 | > 50 and/or gross blood | Hospitalization or packed red blood cells (PRBC) transfusion |

* The laboratory values provided in the tables serve as guidelines and are dependent upon institutional normal parameters. Institutional normal reference ranges should be provided to demonstrate that they are appropriate.

## Appendix 2, COVID-19 case definition

### Suspected case

A disease with clinical signs and epidemiological manifestations:

Clinical findings:

Abrupt onset of fever and cough

Or

Sudden onset of at least three or more symptoms such as fever, cough, general weakness/fatigue, headache, myalgia, sore throat, runny nose, dyspnea, anorexia/nausea/vomiting, diarrhea, loss of consciousness

Epidemiological evidence

Accommodation, employment or travel to areas where the virus is likely to circulate (such as accommodation centers, crowded places, conferences and ceremonies, health centers, etc.) during the last 14 days

B) A patient with severe acute respiratory infection (SARI) needs to be hospitalized with the onset of symptoms within the last 10 days

### Probable case

A) A suspected patient who is in close contact with a probable or confirmed patient or a cluster of patients with at least one confirmed case report among them

B) A suspected patient with imaging findings in favor of COVID-19

• Such as unilateral or bilateral multilobar infiltrates, especially peripheral infiltration in CT Scan of lung or chest radiograph and clinically confirmed pulmonary ground-glass opacity

A patient experiencing acute loss of sense of smell or taste

D) Death in a patient with suspected COVID-19 (above criteria), not justified by any other reason

### Confirmed case

Patient with laboratory confirmation of the presence of SARS-CoV-2, regardless of the appearance of clinical signs and symptoms

### Close contact

A person who has been in contact with the probable or confirmed case within two days before to 14 days after the onset of symptoms in the following circumstances:

1) Face to face contact at a distance of less than 1 meter for at least 15 minutes

2) Direct physical contact with the probable or confirmed case

3) Taking care of probable or confirmed case without using appropriate personal protective equipment

Or

4) In other situations, examinations are performed based on the probability of local transmission.

## Appendix 3, Randomization sequence list


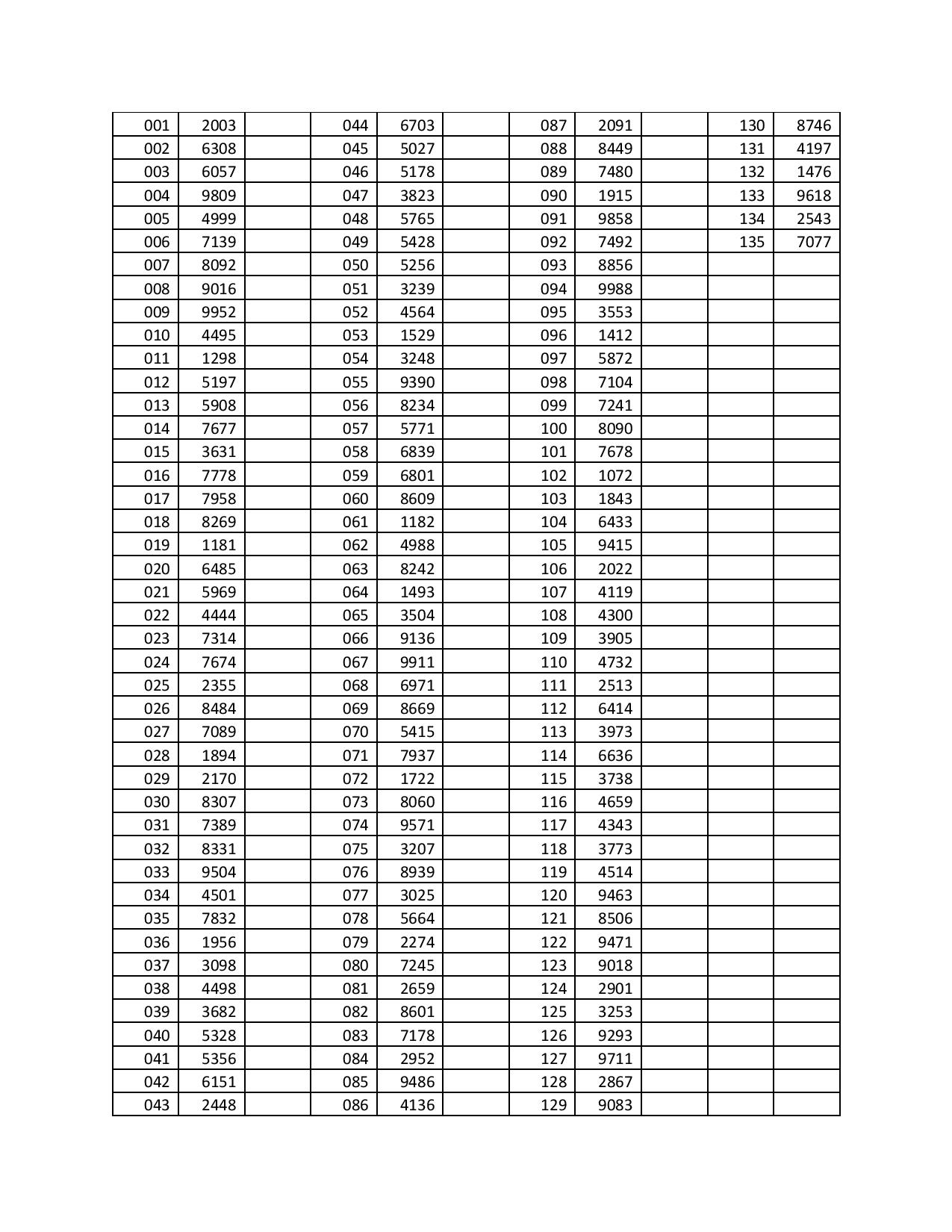

Supplement: Supplementary data 2 [file mmc2.docx]
