# Supplementary material for: Safety and immunogenicity of an inactivated SARS-CoV-2 vaccine (FAKHRAVAC®) in healthy adults aged 18–55 years: Randomized, double-blind, placebo-controlled, phase I clinical trial
Source: Vaccine X. 2023 Oct 27;15:100401. doi: 10.1016/j.jvacx.2023.100401 (PMC10628354; doi:10.1016/j.jvacx.2023.100401)

**FAKHRAVAC**

**Phase 1**

**Results**

Contents

[Baseline comparisons 8](#_Toc102994733)

[Participant flow diagram 8](#_Toc102994734)

[Comparison of participant's baseline characteristics 9](#_Toc102994735)

[Comparison of demographic information in the sentinel group 9](#_Toc102994736)

[Comparison of demographic information in randomized participants 10](#_Toc102994737)

[Comparison of baseline vital signs on screening day 11](#_Toc102994738)

[Comparison of baseline laboratory results on screening day 12](#_Toc102994739)

[Safety outcomes 14](#_Toc102994740)

[Medically Attended Adverse Events (MAAE) 14](#_Toc102994741)

[Vital signs at the time of vaccination 15](#_Toc102994742)

[First injection 15](#_Toc102994743)

[Second injection 17](#_Toc102994744)

[Local adverse reactions 19](#_Toc102994745)

[First injection 19](#_Toc102994746)

[Second injection 20](#_Toc102994747)

[Systemic adverse reactions 21](#_Toc102994748)

[First injection 21](#_Toc102994749)

[Second injection 22](#_Toc102994750)

[Laboratory findings 23](#_Toc102994751)

[First injection 24](#_Toc102994752)

[Second injection 26](#_Toc102994753)

[Immunogenicity outcomes 28](#_Toc102994754)

[Neutralizing antibody activity 28](#_Toc102994755)

[Serum ELISA IgG levels for SARS-CoV-2 31](#_Toc102994756)

[Serum IgG levels for S antigen 31](#_Toc102994757)

[Serum IgG levels for Nucleocapsid antigen 35](#_Toc102994758)

[Serum ELISA lymphokine levels 39](#_Toc102994759)

[Peripheral blood flowcytometry for lymphocyte subtypes composition 44](#_Toc102994760)

Index of tables

[Table 1. Comparison of baseline characteristics in the sentinel group 9](#_Toc102994761)

[Table 2. Comparison of baseline characteristics in randomized participants 10](#_Toc102994762)

[Table 3. Comparison of baseline vital signs in different vaccine strengths of 0.5 × 10^6^ TCID_50_ (5 µg/dose), 2.5 × 10^6^ TCID_50_ (10 µg/dose) and placebo in the sentinel group 11](#_Toc102994763)

[Table 4. Comparison of baseline vital signs in two injection schedules and different vaccine strengths of 0.5 × 10^6^ TCID_50_ (5 µg/dose), 2.5 × 10^6^ TCID_50_ (10 µg/dose) and placebo in the randomized participants 11](#_Toc102994764)

[Table 5. Comparison of baseline laboratory results in different vaccine strengths of 0.5 × 10^6^ TCID_50_ (5 µg/dose), 2.5 × 10^6^ TCID_50_ (10 µg/dose) and placebo in the sentinel group 12](#_Toc102994765)

[Table 6. . Comparison of baseline laboratory results in two injection schedules and different vaccine strengths of 0.5 × 10^6^ TCID_50_ (5 µg/dose), 2.5 × 10^6^ TCID_50_ (10 µg/dose) and placebo in the randomized participants 13](#_Toc102994766)

[Table 7. Frequncy of seeking medical attention or receiving medication in two injection schedules and different vaccine strengths of 0.5 × 10^6^ TCID_50_ (5 µg/dose), 2.5 × 10^6^ TCID_50_ (10 µg/dose) and placebo 14](#_Toc102994767)

[Table 8. Vital signs at the time of 1^st^ injection in two administration schedules and different vaccine strengths of 0.5 × 10^6^ TCID_50_ (5 µg/dose), 2.5 × 10^6^ TCID_50_ (10 µg/dose) and placebo 15](#_Toc102994768)

[Table 9. Grading of abnormal vital signs at the time of 1^st^ injection in two administration schedules and different vaccine strengths of 0.5 × 10^6^ TCID_50_ (5 µg/dose), 2.5 × 10^6^ TCID_50_ (10 µg/dose) and placebo 16](#_Toc102994769)

[Table 10. Vital signs at the time of 2^nd^ injection in two administration schedules and different vaccine strengths of 0.5 × 10^6^ TCID_50_ (5 µg/dose), 2.5 × 10^6^ TCID_50_ (10 µg/dose) and placebo 17](#_Toc102994770)

[Table 11. Grading of abnormal vital signs at the time of 2^nd^ injection in two administration schedules and different vaccine strengths of 0.5 × 10^6^ TCID_50_ (5 µg/dose), 2.5 × 10^6^ TCID_50_ (10 µg/dose) and placebo 18](#_Toc102994771)

[Table 12. Grades of local adverse reactions in the six days following the 1^st^ injection in two administration schedules and different vaccine strengths of 0.5 × 10^6^ TCID_50_ (5 µg/dose), 2.5 × 10^6^ TCID_50_ (10 µg/dose) and placebo 19](#_Toc102994772)

[Table 13. Grades of local adverse reactions in the six days following the 2^nd^ injection in two administration schedules and different vaccine strengths of 0.5 × 10^6^ TCID_50_ (5 µg/dose), 2.5 × 10^6^ TCID_50_ (10 µg/dose) and placebo 20](#_Toc102994773)

[Table 14. Grades of systemic adverse reactions in the six days following the 1^st^ injection in two administration schedules and different vaccine strengths of 0.5 × 10^6^ TCID_50_ (5 µg/dose), 2.5 × 10^6^ TCID_50_ (10 µg/dose) and placebo 21](#_Toc102994774)

[Table 15. Grades of systemic adverse reactions in the six days following the 2^nd^ injection in two administration schedules and different vaccine strengths of 0.5 × 10^6^ TCID_50_ (5 µg/dose), 2.5 × 10^6^ TCID_50_ (10 µg/dose) and placebo 22](#_Toc102994775)

[Table 16. Median and interquartile range (IQR) of laboratory findings at screening day and one week after each injection in two administration schedules and different vaccine strengths of 0.5 × 10^6^ TCID_50_ (5 µg/dose), 2.5 × 10^6^ TCID_50_ (10 µg/dose) and placebo 23](#_Toc102994776)

[Table 17. Grading of abnormal laboratory findings one week after 1^st^ injection in two administration schedules and different vaccine strengths of 0.5 × 10^6^ TCID_50_ (5 µg/dose), 2.5 × 10^6^ TCID_50_ (10 µg/dose) and placebo 24](#_Toc102994777)

[Table 18. Grading of abnormal laboratory findings one week after 2^nd^ injection in two administration schedules and different vaccine strengths of 0.5 × 10^6^ TCID_50_ (5 µg/dose), 2.5 × 10^6^ TCID_50_ (10 µg/dose) and placebo 26](#_Toc102994778)

[Table 19. Geometric mean titer, geometric mean ratio (compared to placebo), and geometric mean fold increase (compared to day zero) for serum neutralizing antibody titers and their 95% confidence intervals at predefined time intervals in two administration schedules and different vaccine strengths of 0.5 × 10^6^ TCID_50_ (5 µg/dose), 2.5 × 10^6^ TCID_50_ (10 µg/dose) and placebo (has been included in the main manuscript) 28](#_Toc102994779)

[Table 20. Proportion of participants with four-fold increase in neutralizing antibody titer at predefined time intervals in two administration schedules and different vaccine strengths of 0.5 × 10^6^ TCID_50_ (5 µg/dose), 2.5 × 10^6^ TCID_50_ (10 µg/dose) and placebo (has been included in the main manuscript) 30](#_Toc102994780)

[Table 21. Geometric mean, geometric mean ratio (compared to placebo), geometric mean fold increase (compared to day zero), and geometric mean fold ratio for serum specific IgG antibody levels against S antigen and their 95% confidence intervals at predefined time intervals in two administration schedules and different vaccine strengths of 0.5 × 10^6^ TCID_50_ (5 µg/dose), 2.5 × 10^6^ TCID_50_ (10 µg/dose) and placebo 31](#_Toc102994781)

[Table 22. Geometric mean, geometric mean ratio (compared to placebo), geometric mean fold increase (compared to day zero), and geometric mean fold ratio for serum specific IgG antibody levels against N antigen and their 95% confidence intervals at predefined time intervals in two administration schedules and different vaccine strengths of 0.5 × 10^6^ TCID_50_ (5 µg/dose), 2.5 × 10^6^ TCID_50_ (10 µg/dose) and placebo 35](#_Toc102994782)

Index of figures

[Figure 1. Participant flow diagram (has been included in the main manuscript) 8](#_Toc102994783)

[Figure 2. Proportion of participants experiencing local adverse reactions in the six days following the 1^st^ injection in two administration schedules and different vaccine strengths of 0.5 × 10^6^ TCID_50_ (5 µg/dose), 2.5 × 10^6^ TCID_50_ (10 µg/dose) and placebo 19](#_Toc102994784)

[Figure 3. Proportion of participants experiencing local adverse reactions in the six days following the 2^nd^ injection in two administration schedules and different vaccine strengths of 0.5 × 10^6^ TCID_50_ (5 µg/dose), 2.5 × 10^6^ TCID_50_ (10 µg/dose) and placebo 20](#_Toc102994785)

[Figure 4. Proportion of participants experiencing systemic adverse reactions in the six days following the 1^st^ injection in two administration schedules and different vaccine strengths of 0.5 × 10^6^ TCID_50_ (5 µg/dose), 2.5 × 10^6^ TCID_50_ (10 µg/dose) and placebo 21](#_Toc102994786)

[Figure 5. Proportion of participants experiencing systemic adverse reactions in the six days following the 2^nd^ injection in two administration schedules and different vaccine strengths of 0.5 × 10^6^ TCID_50_ (5 µg/dose), 2.5 × 10^6^ TCID_50_ (10 µg/dose) and placebo 22](#_Toc102994787)

[Figure 6. Changes in serum neutralizing antibody titers over the study period for each individual participant and the group mean in two administration schedules and different vaccine strengths of 0.5 × 10^6^ TCID_50_ (5 µg/dose), 2.5 × 10^6^ TCID_50_ (10 µg/dose) and placebo 29](#_Toc102994788)

[Figure 7. Changes in group means of serum neutralizing antibody titers over the study period in two administration schedules and different vaccine strengths of 0.5 × 10^6^ TCID_50_ (5 µg/dose), 2.5 × 10^6^ TCID_50_ (10 µg/dose) and placebo 29](#_Toc102994789)

[Figure 8. Changes in serum specific IgG antibody levels against S antigen over the study period for each individual participant and the group mean in two administration schedules and different vaccine strengths of 0.5 × 10^6^ TCID_50_ (5 µg/dose), 2.5 × 10^6^ TCID_50_ (10 µg/dose) and placebo 33](#_Toc102994790)

[Figure 9. Changes in group means of serum specific IgG antibody levels against S antigen over the study period in two administration schedules and different vaccine strengths of 0.5 × 10^6^ TCID_50_ (5 µg/dose), 2.5 × 10^6^ TCID_50_ (10 µg/dose) and placebo 33](#_Toc102994791)

[Figure 10. Serum specific IgG antibody levels against S antigen over the study period for each individual participant and the group mean and its 95% confidence interval in two administration schedules and different vaccine strengths of 0.5 × 10^6^ TCID_50_ (5 µg/dose), 2.5 × 10^6^ TCID_50_ (10 µg/dose) and placebo 34](#_Toc102994792)

[Figure 11. Serum specific IgG antibody levels against S antigen in natural logarithmic scale over the study period for each individual participant and the group mean and its 95% confidence interval in two administration schedules and different vaccine strengths of 0.5 × 10^6^ TCID_50_ (5 µg/dose), 2.5 × 10^6^ TCID_50_ (10 µg/dose) and placebo 34](#_Toc102994793)

[Figure 12. Changes in serum specific IgG antibody levels against N antigen over the study period for each individual participant and the group mean in two administration schedules and different vaccine strengths of 0.5 × 10^6^ TCID_50_ (5 µg/dose), 2.5 × 10^6^ TCID_50_ (10 µg/dose) and placebo 37](#_Toc102994794)

[Figure 13. Changes in group means of serum specific IgG antibody levels against N antigen over the study period in two administration schedules and different vaccine strengths of 0.5 × 10^6^ TCID_50_ (5 µg/dose), 2.5 × 10^6^ TCID_50_ (10 µg/dose) and placebo 37](#_Toc102994795)

[Figure 14. Serum specific IgG antibody levels against N antigen over the study period for each individual participant and the group mean and its 95% confidence interval in two administration schedules and different vaccine strengths of 0.5 × 10^6^ TCID_50_ (5 µg/dose), 2.5 × 10^6^ TCID_50_ (10 µg/dose) and placebo 38](#_Toc102994796)

[Figure 15. Serum specific IgG antibody levels against N antigen in natural logarithmic scale over the study period for each individual participant and the group mean and its 95% confidence interval in two administration schedules and different vaccine strengths of 0.5 × 10^6^ TCID_50_ (5 µg/dose), 2.5 × 10^6^ TCID_50_ (10 µg/dose) and placebo 38](#_Toc102994797)

[Figure 16. Serum concentrations pg/ml of IL-6 in study participants at day 0 and 14 days after 2^nd^ injection in two administration schedules and different vaccine strengths of 0.5 × 10^6^ TCID_50_ (5 µg/dose), 2.5 × 10^6^ TCID_50_ (10 µg/dose) and placebo 39](#_Toc102994798)

[Figure 17. Serum concentrations pg/ml of IL-2 in study participants at day 0 and 14 days after 2^nd^ injection in two administration schedules and different vaccine strengths of 0.5 × 10^6^ TCID_50_ (5 µg/dose), 2.5 × 10^6^ TCID_50_ (10 µg/dose) and placebo 39](#_Toc102994799)

[Figure 18. Serum concentrations pg/ml of IL-4 in study participants at day 0 and 14 days after 2^nd^ injection in two administration schedules and different vaccine strengths of 0.5 × 10^6^ TCID_50_ (5 µg/dose), 2.5 × 10^6^ TCID_50_ (10 µg/dose) and placebo 40](#_Toc102994800)

[Figure 19. Serum concentrations pg/ml of IL-5 in study participants at day 0 and 14 days after 2^nd^ injection in two administration schedules and different vaccine strengths of 0.5 × 10^6^ TCID_50_ (5 µg/dose), 2.5 × 10^6^ TCID_50_ (10 µg/dose) and placebo 40](#_Toc102994801)

[Figure 20. Serum concentrations pg/ml of TNF-α in study participants at day 0 and 14 days after 2^nd^ injection in two administration schedules and different vaccine strengths of 0.5 × 10^6^ TCID_50_ (5 µg/dose), 2.5 × 10^6^ TCID_50_ (10 µg/dose) and placebo 41](#_Toc102994802)

[Figure 21. Serum concentrations pg/ml of IL-10 in study participants at day 0 and 14 days after 2^nd^ injection in two administration schedules and different vaccine strengths of 0.5 × 10^6^ TCID_50_ (5 µg/dose), 2.5 × 10^6^ TCID_50_ (10 µg/dose) and placebo 41](#_Toc102994803)

[Figure 22. Serum concentrations pg/ml of IL-12 in study participants at day 0 and 14 days after 2^nd^ injection in two administration schedules and different vaccine strengths of 0.5 × 10^6^ TCID_50_ (5 µg/dose), 2.5 × 10^6^ TCID_50_ (10 µg/dose) and placebo 42](#_Toc102994804)

[Figure 23. Serum concentrations pg/ml of IL-17 in study participants at day 0 and 14 days after 2^nd^ injection in two administration schedules and different vaccine strengths of 0.5 × 10^6^ TCID_50_ (5 µg/dose), 2.5 × 10^6^ TCID_50_ (10 µg/dose) and placebo 42](#_Toc102994805)

[Figure 24. Serum concentrations pg/ml of γ-INF in study participants at day 0 and 14 days after 2^nd^ injection in two administration schedules and different vaccine strengths of 0.5 × 10^6^ TCID_50_ (5 µg/dose), 2.5 × 10^6^ TCID_50_ (10 µg/dose) and placebo 43](#_Toc102994806)

[Figure 25. Proportion of lymphocytes in peripheral blood measured by flowcytometry at day 0 and two weeks after 2^nd^ injection in two administration schedules and different vaccine strengths of 0.5 × 10^6^ TCID_50_ (5 µg/dose), 2.5 × 10^6^ TCID_50_ (10 µg/dose) and placebo 44](#_Toc102994807)

[Figure 26. Proportion of lymphocytic population expressing CD3 marker in peripheral blood measured by flowcytometry at day 0 and two weeks after 2^nd^ injection in two administration schedules and different vaccine strengths of 0.5 × 10^6^ TCID_50_ (5 µg/dose), 2.5 × 10^6^ TCID_50_ (10 µg/dose) and placebo 44](#_Toc102994808)

[Figure 27. Proportion of lymphocytic population expressing CD4 marker within CD3 positive subset in peripheral blood measured by flowcytometry at day 0 and two weeks after 2^nd^ injection in two administration schedules and different vaccine strengths of 0.5 × 10^6^ TCID_50_ (5 µg/dose), 2.5 × 10^6^ TCID_50_ (10 µg/dose) and placebo 45](#_Toc102994809)

[Figure 28. Proportion of lymphocytic population expressing CD8 marker within CD3 positive subset in peripheral blood measured by flowcytometry at day 0 and two weeks after 2^nd^ injection in two administration schedules and different vaccine strengths of 0.5 × 10^6^ TCID_50_ (5 µg/dose), 2.5 × 10^6^ TCID_50_ (10 µg/dose) and placebo 45](#_Toc102994810)

[Figure 29. CD4/CD8 ratio within CD3 positive lymphocytic subset in peripheral blood measured by flowcytometry at day 0 and two weeks after 2^nd^ injection in two administration schedules and different vaccine strengths of 0.5 × 10^6^ TCID_50_ (5 µg/dose), 2.5 × 10^6^ TCID_50_ (10 µg/dose) and placebo 46](#_Toc102994811)

[Figure 30. CD4/CD8 ratio within lymphocytic cell population in peripheral blood measured by flowcytometry at day 0 and two weeks after 2^nd^ injection in two administration schedules and different vaccine strengths of 0.5 × 10^6^ TCID_50_ (5 µg/dose), 2.5 × 10^6^ TCID_50_ (10 µg/dose) and placebo 46](#_Toc102994812)

[Figure 31. Proportion of lymphocytic population expressing CD56 marker within CD3 negative subset in peripheral blood measured by flowcytometry at day 0 and two weeks after 2^nd^ injection in two administration schedules and different vaccine strengths of 0.5 × 10^6^ TCID_50_ (5 µg/dose), 2.5 × 10^6^ TCID_50_ (10 µg/dose) and placebo 47](#_Toc102994813)

[Figure 32. Proportion of CD19 or CD20 in peripheral blood measured by flowcytometry at day 0 and two weeks after 2^nd^ injection in two administration schedules and different vaccine strengths of 0.5 × 10^6^ TCID_50_ (5 µg/dose), 2.5 × 10^6^ TCID_50_ (10 µg/dose) and placebo 47](#_Toc102994814)

# Baseline comparisons

## Participant flow diagram

Figure 1. Participant flow diagram (has been included in the main manuscript)


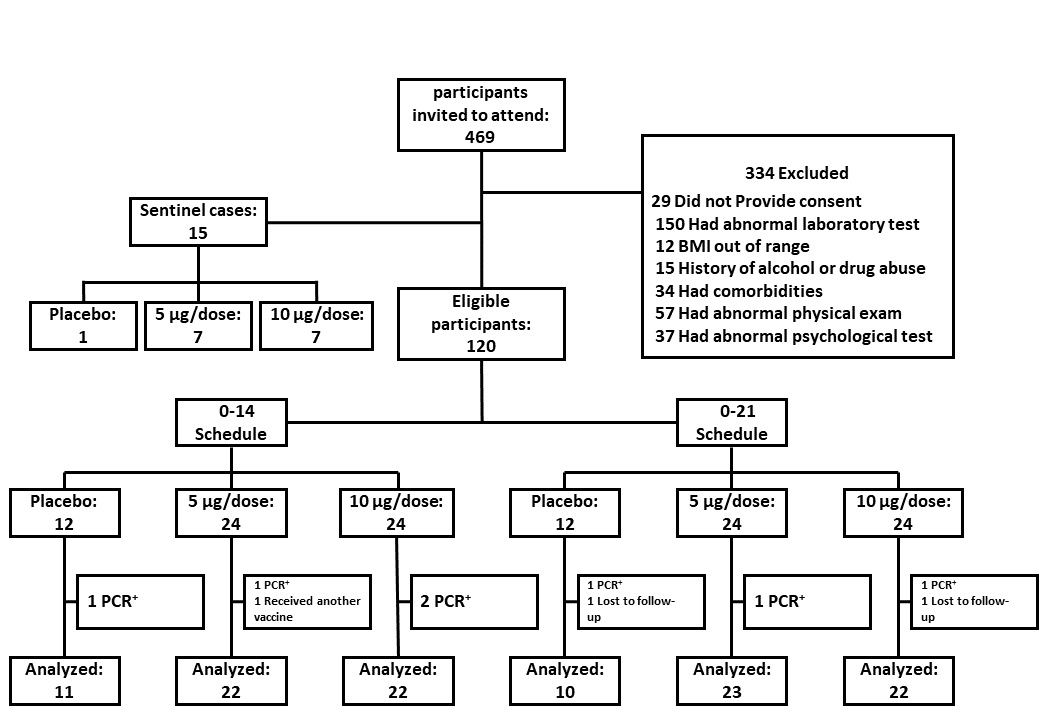


## Comparison of participant's baseline characteristics

### Comparison of demographic information in the sentinel group

Table 1. Comparison of baseline characteristics in the sentinel group

| **Characteristic** | **Overall, N = 15** | **Placebo, N = 1** | **Strength 0.5, N = 7** | **Strength 2.5, N = 7** |
| --- | --- | --- | --- | --- |
| **Sex** |  |  |  |  |
| Male | 15 (100%) | 1 | 7 (100%) | 7 (100%) |
| Female | 0 | 0 | 0 | 0 |
| **Age (Year)** | 36.0 (30.0, 37.5) | 38.0 | 36.0 (29.0, 37.5) | 35.0 (30.0, 36.5) |
| **BMI** | 27.6 (22.4, 30.8) | 30.8 | 26.2 (21.9, 30.8) | 27.6 (24.4, 29.8) |
| **Smoking** |  |  |  |  |
| Current Smoker | 3 (20%) | 1 | 1 (14%) | 1 (14%) |
| Ex-smoker | 4 (27%) | 0 | 3 (43%) | 1 (14%) |
| Never-smoker | 8 (53%) | 0 | 3 (43%) | 5 (71%) |
| **Education** |  | 0 |  |  |
| Diploma | 7 (47%) | 0 | 3 (43%) | 4 (57%) |
| Bachelor | 4 (27%) | 0 | 3 (43%) | 1 (14%) |
| Master | 2 (13%) | 0 | 1 (14%) | 1 (14%) |
| Doctoral and above | 2 (13%) | 1 | 0 | 1 (14%) |
| **Job** |  |  |  |  |
| Government employee | 2 (13%) | 0 | 1 (14%) | 1 (14%) |
| Private employee | 4 (27%) | 0 | 0 | 4 (57%) |
| Self-employed | 7 (47%) | 1 | 4 (57%) | 2 (29%) |
| Student | 2 (13%) | 0 | 2 (29%) | 0 |
| n (%); Median (IQR) | | | | |

### Comparison of demographic information in randomized participants

Table 2. Comparison of baseline characteristics in randomized participants

|  | Schedule 0-14 | | | Schedule 0-21 | | |
| --- | --- | --- | --- | --- | --- | --- |
| **Characteristic** | **Placebo,**  **N = 12** | **Strength 0.5,**  **N = 24** | **Strength 2.5,**  **N = 24** | **Placebo,**  **N = 12** | **Strength 0.5,**  **N = 24** | **Strength 2.5,**  **N = 24** |
| **Sex** |  |  |  |  |  |  |
| Male | 8 (67%) | 12 (50%) | 17 (71%) | 10 (83%) | 17 (71%) | 15 (62%) |
| Female | 4 (33%) | 12 (50%) | 7 (29%) | 2 (17%) | 7 (29%) | 9 (38%) |
| **Age (Year)** |  |  |  |  |  |  |
| Median (Q1,Q3) | 34 (24, 44) | 36 (31, 40) | 35 (30, 41) | 37 (24, 41) | 30 (25, 38) | 38 (35, 42) |
| Mean (SD) | 34.5 (10.95) | 35.29 (6.58) | 34.88 (9.42) | 34 (9.90) | 32.75 (10.27) | 37.96 (6.97) |
| Median (min-max) | 34 (20 - 51) | 35.5 (22 - 52) | 35 (19 - 52) | 37 (19 - 47) | 30 (20 - 54) | 38 (22 – 49) |
| **BMI** |  |  |  |  |  |  |
| Median  (Q1,Q3) | 23.8  (20.4, 26.0) | 25.8  (23.3, 28.2) | 25.0  (21.6, 29.1) | 27.6  (23.1, 29.0) | 26.1  (24.0, 27.7) | 24.8  (21.9, 28.5) |
| Mean (SD) | 24.16 (4.38) | 24.65 (3.23) | 25.59 (4.12) | 26.71 (3.64) | 25.86 (3.70) | 25.84 (4.99) |
| Median  (min-max) | 23.8  (19.4 – 33.6) | 25.8  (20.3 – 31.5) | 24.95  (19.5 – 32.8) | 27.6  (21 – 32.8) | 26.1  (19.1 – 32.6) | 24.8  (18.4 – 35.1) |
| **Smoking** |  |  |  |  |  |  |
| Current Smoker | 2 (17%) | 1 (4.2%) | 2 (8.3%) | 6 (50%) | 3 (12%) | 5 (21%) |
| Ex-smoker | 0 (0%) | 1 (4.2%) | 5 (21%) | 0 (0%) | 4 (17%) | 4 (17%) |
| Never-smoker | 10 (83%) | 22 (92%) | 17 (71%) | 6 (50%) | 17 (71%) | 15 (62%) |
| **Education** |  |  |  |  |  |  |
| Elementary | 1 (8.3%) | 0 (0%) | 0 (0%) | 1 (8.3%) | 0 (0%) | 1 (4.2%) |
| Diploma | 3 (25%) | 6 (25%) | 7 (29%) | 3 (25%) | 10 (42%) | 7 (29%) |
| Bachelor | 6 (50%) | 10 (42%) | 7 (29%) | 5 (42%) | 10 (42%) | 9 (38%) |
| Master | 2 (17%) | 5 (21%) | 9 (38%) | 3 (25%) | 3 (12%) | 5 (21%) |
| Doctoral and above | 0 (0%) | 3 (12%) | 1 (4.2%) | 0 (0%) | 1 (4.2%) | 2 (8.3%) |
| **Job** |  |  |  |  |  |  |
| Unemployed/Retired | 0 (0%) | 1 (4.2%) | 0 (0%) | 1 (8.3%) | 2 (8.3%) | 2 (8.3%) |
| Government employee | 1 (8.3%) | 9 (38%) | 12 (50%) | 3 (25%) | 3 (12%) | 9 (38%) |
| Private employee | 2 (17%) | 2 (8.3%) | 2 (8.3%) | 1 (8.3%) | 4 (17%) | 4 (17%) |
| Self-employed | 3 (25%) | 6 (25%) | 5 (21%) | 2 (17%) | 8 (33%) | 5 (21%) |
| Student | 2 (17%) | 2 (8.3%) | 3 (12%) | 4 (33%) | 4 (17%) | 1 (4.2%) |
| Housewife | 4 (33%) | 4 (17%) | 2 (8.3%) | 1 (8.3%) | 3 (12%) | 3 (12%) |
|  | | | | | | |

## Comparison of baseline vital signs on screening day

Table 3. Comparison of baseline vital signs in different vaccine strengths of 0.5 × 10^6^ TCID_50_ (5 µg/dose), 2.5 × 10^6^ TCID_50_ (10 µg/dose) and placebo in the sentinel group

| **Characteristic** | **Placebo, N = 1** | **Strength 0.5, N = 7** | **Strength 2.5, N = 7** |
| --- | --- | --- | --- |
| Body temperature(°C) | 36.20 (36.20, 36.20) | 36.60 (36.50, 36.75) | 36.60 (36.55, 36.70) |
| O2 saturation | 97 (97, 97) | 97 (96, 98) | 97 (97, 98) |
| Heart rate (Per minute) | 75 (75, 75) | 78 (76, 86) | 84 (74, 100) |
| Respiration rate (Per minute) | 14 (14, 14) | 17 (16, 18) | 17 (16, 18) |
| Systolic blood pressure(mmHg) | 110 (110, 110) | 110 (110, 120) | 120 (110, 120) |
| Diastolic blood essure(mmHg) | 80 (80, 80) | 80 (70, 80) | 80 (75, 80) |
| Median (IQR) | | | |

Table 4. Comparison of baseline vital signs in two injection schedules and different vaccine strengths of 0.5 × 10^6^ TCID_50_ (5 µg/dose), 2.5 × 10^6^ TCID_50_ (10 µg/dose) and placebo in the randomized participants

|  | **Schedule 0-14** | | | **Schedule 0-21** | | |  |
| --- | --- | --- | --- | --- | --- | --- | --- |
| **Characteristic** | **Placebo, N = 12** | **Strength 0.5, N = 24** | **Strength 2.5, N = 24** | **Placebo, N = 12** | **Strength 0.5, N = 24** | **Strength 2.5, N = 24** |  |
| Body temperature(°C) | 36.7 (36.55, 36.73) | 36.7 (36.5, 36.7) | 36.7 (36.5, 36.8) | 36.65 (36.38, 36.73) | 36.6 (36.5, 36.7) | 36.7 (36.48, 36.7) |  |
| O2 saturation | 98 (98, 98) | 98 (97, 98) | 98 (97, 98) | 97 (96, 98) | 97 (97, 98) | 98 (97, 98) |  |
| Heart rate(Per minute) | 80 (75, 91) | 83 (75, 88) | 79 (74, 89) | 83 (81, 86) | 80 (77, 87) | 78 (75, 82) |  |
| Respiration rate(Per minute) | 18 (16, 18) | 18 (17, 18) | 19 (17, 20) | 17 (17, 19) | 18 (17, 18) | 18 (17, 19) |  |
| Systolic blood sure(mmHg) | 110 (110, 121) | 120 (112, 122) | 114 (110, 123) | 120 (115, 125) | 120 (110, 125) | 116 (110, 126) |  |
| Diastolic blood pressure(mmHg) | 80 (76, 81) | 78 (70, 80) | 80 (74, 80) | 80 (76, 85) | 80 (75, 81) | 78 (74, 82) |  |
| Median (IQR) | | | | | | | |

## Comparison of baseline laboratory results on screening day

Table 5. Comparison of baseline laboratory results in different vaccine strengths of 0.5 × 10^6^ TCID_50_ (5 µg/dose), 2.5 × 10^6^ TCID_50_ (10 µg/dose) and placebo in the sentinel group

| **Characteristic** | **Overall, N = 15** | **Placebo, N = 1** | **Strength 0.5, N =7** | **Strength 2.5, N =7** |
| --- | --- | --- | --- | --- |
| Hemoglobin mg/dL | 15.90 (15.70, 16.65) | 16.20 (16.20, 16.20) | 16.20 (15.80, 16.65) | 15.80 (14.70, 16.30) |
| WBC cell/mm3×103 | 5.46 (4.84, 6.78) | 8.20 (8.20, 8.20) | 5.16 (4.37, 5.90) | 6.20 (5.42, 6.78) |
| Lymphocytes cell/mm3×103 | 1.70 (1.58, 1.90) | 2.71 (2.71, 2.71) | 1.79 (1.60, 1.90) | 1.66 (1.53, 1.75) |
| Neutrophils cell/mm3×103 | 3.40 (2.47, 4.36) | 4.65 (4.65, 4.65) | 2.52 (2.04, 3.69) | 4.08 (3.11, 4.42) |
| Eosinophils - cell/mm3×103 | 0.14 (0.09, 0.20) | 0.34 (0.34, 0.34) | 0.14 (0.09, 0.17) | 0.14 (0.09, 0.19) |
| Platelets cell/mm3×103 | 261 (212, 301) | 353 (353, 353) | 247 (212, 298) | 261 (220, 288) |
| BUN mg/dL | 14.00 (12.60, 15.70) | 12.10 (12.10, 12.10) | 14.00 (12.35, 15.35) | 14.10 (13.35, 16.60) |
| Creatinine - mg/dL | 1.00 (0.89, 1.11) | 1.04 (1.04, 1.04) | 1.02 (0.92, 1.17) | 0.98 (0.85, 1.05) |
| AST IU/L | 20.0 (18.0, 22.5) | 15.0 (15.0, 15.0) | 20.0 (18.5, 21.0) | 22.0 (19.5, 23.5) |
| ALT IU/L | 19 (15, 26) | 16 (16, 16) | 18 (14, 26) | 23 (20, 26) |
| Alkaline phosphatase IU/L | 191 (147, 230) | 135 (135, 135) | 222 (195, 246) | 170 (144, 206) |
| Bilirubin total | 0.67 (0.53, 0.83) | 0.76 (0.76, 0.76) | 0.67 (0.59, 0.88) | 0.56 (0.47, 0.81) |
| Sodium, mEq/L | 139 (138, 140.5) | 139 (139.00, 139.00) | 140 (139.50, 141.50) | 138 (138.00, 139.50) |
| Median (IQR) | | | | |

Table 6. . Comparison of baseline laboratory results in two injection schedules and different vaccine strengths of 0.5 × 10^6^ TCID_50_ (5 µg/dose), 2.5 × 10^6^ TCID_50_ (10 µg/dose) and placebo in the randomized participants

|  |  | **Schedule 0-14** | | | **Schedule 0-21** | | |
| --- | --- | --- | --- | --- | --- | --- | --- |
| **Characteristic** | **Overall,**  **N = 120** | **Placebo,**  **N = 12** | **Strength 0.5,**  **N = 24** | **Strength 2.5,**  **N = 24** | **Placebo,**  **N = 12** | **Strength 0.5,**  **N = 24** | **Strength 2.5,**  **N = 24** |
| Hemoglobin mg/dL | 15.30  (14.10, 16.10) | 15.15  (14.37, 15.83) | 14.20  (13.58, 15.55) | 15.60  (14.60, 16.70) | 15.60  (15.20, 16.55) | 15.40  (14.47, 16.10) | 14.70  (13.70, 16.13) |
| WBC cell/mm3×103 | 6.10  (5.39, 7.01) | 6.15  (5.60, 6.60) | 5.44  (5.14, 6.15) | 6.40  (5.88, 7.50) | 6.10  (5.59, 7.31) | 6.20  (5.03, 6.78) | 6.23  (5.68, 6.72) |
| Lymphocytes cell/mm3×103 | 1.88  (1.69, 2.21) | 1.95  (1.83, 2.11) | 1.79  (1.66, 2.00) | 1.97  (1.81, 2.48) | 1.98  (1.75, 2.12) | 1.83  (1.62, 2.16) | 1.88  (1.62, 2.34) |
| Neutrophils cell/mm3×103 | 3.59  (3.06, 4.33) | 3.81  (3.11, 4.36) | 3.15  (2.92, 3.75) | 4.02  (3.53, 4.63) | 3.80  (3.25, 4.34) | 3.44  (2.92, 4.25) | 3.65  (3.25, 4.19) |
| Eosinophils - cell/mm3×103 | 0.14  (0.08, 0.22) | 0.10  (0.09, 0.13) | 0.14  (0.07, 0.19) | 0.17  (0.09, 0.23) | 0.18  (0.12, 0.22) | 0.11  (0.08, 0.19) | 0.15  (0.09, 0.26) |
| Platelets cell/mm3×103 | 250  (214, 285) | 269  (216, 312) | 234  (204, 287) | 262  (234, 281) | 254  (246, 284) | 238  (211, 289) | 238  (204, 277) |
| BUN mg/dL | 12.1  (10.3, 15.3) | 13.20  (10.62, 15.15) | 12.05  (10.47, 15.15) | 13.25  (10.87, 16.18) | 11.5  (10.3, 12.3) | 11.1  (9.3, 15.5) | 12.2  (11.0, 15.6) |
| Creatinine - mg/dL | 0.98  (0.83, 1.11) | 0.94  (0.82, 1.08) | 0.93  (0.81, 1.03) | 1.00  (0.84, 1.14) | 1.05  (0.82, 1.16) | 0.97  (0.92, 1.06) | 1.00  (0.90, 1.13) |
| AST IU/L | 17.0  (15.0, 20.0) | 16.5  (14.8, 20.2) | 17.0  (14.0, 20.0) | 18. 0  (16.8, 21.0) | 17.5  (14.5, 19.5) | 17.0  (16.0, 21.0) | 16.0  (14.0, 18.2) |
| ALT IU/L | 18  (12, 25) | 16  (10, 22) | 16  (11, 20) | 20  (15, 30) | 20  (18, 24) | 18  (15, 26) | 14  (11, 24) |
| Alkaline phosphatase IU/L | 150  (126, 181) | 142  (126, 159) | 138  (118, 154) | 166  (154, 210) | 182  (139, 203) | 162  (128, 180) | 128  (108, 162) |
| Bilirubin total | 0.58  (0.47, 0.76) | 0.72  (0.57, 0.84) | 0.58  (0.40, 0.74) | 0.62  (0.52, 0.86) | 0.62  (0.50, 0.79) | 0.54  (0.46, 0.73) | 0.55  (0.46, 0.64) |
| Sodium, mEq/L | 140  (139, 141) | 139.50  (139, 140) | 140  (139, 141) | 140  (139, 140) | 140  (139.75, 141.25) | 141  (140, 142) | 141  (139, 141) |
| Median (IQR) | | | | | | | |

# Safety outcomes

## Medically Attended Adverse Events (MAAE)

Table 7. Frequncy of seeking medical attention or receiving medication in two injection schedules and different vaccine strengths of 0.5 × 10^6^ TCID_50_ (5 µg/dose), 2.5 × 10^6^ TCID_50_ (10 µg/dose) and placebo

|  | Day 7 | Day 14 | Day 21 | Day 28 | Day 35 | Day 42 | Day 49 | Day 72 | Month 2-3 | Month 4-6 | Total |
| --- | --- | --- | --- | --- | --- | --- | --- | --- | --- | --- | --- |
| **Attend Clinic** |  | | | | | | | | | | 25 |
| Schedule 0-14, Placebo | 1 | 0 | 0 | 1 | 0 | 0 | 0 | 1 | 0 | 0 | 3 |
| Schedule 0-14, Strength 0.5 | 1 | 1 | 0 | 1 | 0 | 0 | 0 | 0 | 0 | 1 | 4 |
| Schedule 0-14, Strength 2.5 | 0 | 0 | 0 | 0 | 0 | 0 | 0 | 0 | 0 | 2 | 2 |
| Schedule 0-21, Placebo | 0 | 0 | 0 | 1 | 0 | 0 | 1 | 0 | 0 | 1 | 3 |
| Schedule 0-21, Strength 0.5 | 0 | 0 | 0 | 0 | 0 | 0 | 0 | 0 | 3 | 1 | 4 |
| Schedule 0-21, Strength 2.5 | 0 | 0 | 1 | 0 | 0 | 1 | 2 | 0 | 0 | 3 | 7 |
| Sentinel | 0 | 0 | 0 | 0 | 0 | 0 | 0 | 0 | 0 | 2 | 2 |
| **Received any Medication** |  | | | | | | | | | | 54 |
| Schedule 0-14, Placebo | 0 | 0 | 2 | 1 | 0 | 0 | 0 | 0 | 1 | 4 | 8 |
| Schedule 0-14, Strength 0.5 | 0 | 0 | 0 | 2 | 0 | 1 | 0 | 0 | 0 | 2 | 5 |
| Schedule 0-14, Strength 2.5 | 1 | 1 | 1 | 0 | 0 | 1 | 0 | 0 | 0 | 5 | 9 |
| Schedule 0-21, Placebo | 0 | 1 | 0 | 0 | 0 | 0 | 0 | 0 | 0 | 2 | 3 |
| Schedule 0-21, Strength 0.5 | 1 | 0 | 0 | 0 | 0 | 0 | 0 | 0 | 3 | 3 | 7 |
| Schedule 0-21, Strength 2.5 | 1 | 2 | 2 | 1 | 1 | 0 | 1 | 0 | 1 | 7 | 16 |
| Sentinel | 1 | 0 | 0 | 1 | 0 | 0 | 0 | 0 | 0 | 4 | 6 |
| **Hospitalized** |  | | | | | | | | | | 3 |
| Schedule 0-14, Placebo | 0 | 0 | 0 | 0 | 0 | 0 | 0 | 1 | 0 | 0 | 1 |
| Schedule 0-14, Strength 0.5 | 0 | 0 | 0 | 0 | 0 | 0 | 0 | 0 | 0 | 0 | 0 |
| Schedule 0-14, Strength 2.5 | 0 | 0 | 0 | 0 | 0 | 0 | 0 | 0 | 0 | 0 | 0 |
| Schedule 0-21, Placebo | 0 | 0 | 0 | 0 | 0 | 0 | 0 | 0 | 0 | 1 | 1 |
| Schedule 0-21, Strength 0.5 | 0 | 0 | 0 | 0 | 0 | 0 | 0 | 0 | 0 | 0 | 0 |
| Schedule 0-21, Strength 2.5 | 0 | 0 | 0 | 0 | 0 | 0 | 0 | 0 | 0 | 1 | 1 |
| Sentinel | 0 | 0 | 0 | 0 | 0 | 0 | 0 | 0 | 0 | 0 | 0 |

## Vital signs at the time of vaccination

### First injection

Table 8. Vital signs at the time of 1^st^ injection in two administration schedules and different vaccine strengths of 0.5 × 10^6^ TCID_50_ (5 µg/dose), 2.5 × 10^6^ TCID_50_ (10 µg/dose) and placebo

|  |  | | **Schedule 0-14** | | | | **Schedule 0-21** | | |
| --- | --- | --- | --- | --- | --- | --- | --- | --- | --- |
| **Characteristic** | **Overall, N = 120** | | **Placebo, N = 12** | | **Strength 0.5, N = 24** | **Strength 2.5, N = 24** | **Placebo, N = 12** | **Strength 0.5, N = 24** | **Strength 2.5, N = 24** |
| **Body Temperature** | | | | |  |  |  |  |  |
| Before IMP | 36.6 (36.5, 36.7) | | 36.7 (36.5, 36.7) | | 36.60 (36.40, 36.70) | 36.50 (36.50, 36.60) | 36.60 (36.50, 36.60) | 36.50 (36.50, 36.70) | 36.50 (36.40, 36.65) |
| After 1 hour | 36.6 (36.5, 36.7) | | 36.7 (36.55, 36.8) | | 36.50 (36.50, 36.60) | 36.60 (36.45, 36.75) | 36.60 (36.52, 36.70) | 36.55 (36.48, 36.70) | 36.60 (36.50, 36.70) |
| After 2 hours | 36.7 (36.5, 36.8) | | 36.8 (36.65, 36.8) | | 36.60 (36.40, 36.70) | 36.70 (36.65, 36.80) | 36.70 (36.50, 36.80) | 36.70 (36.57, 36.80) | 36.50 (36.45, 36.80) |
| After 3 hours | 36.7 (36.5, 36.8) | | 36.7 (36.5, 36.8) | | 36.50 (36.40, 36.70) | 36.70 (36.65, 36.85) | 36.50 (36.43, 36.70) | 36.70 (36.50, 36.90) | 36.50 (36.50, 36.60) |
| **Systolic Blood Pressure** | | | |  | |  |  |  |  |
| Before IMP | 115 (106, 120) | | 114 (105, 120) | | 113 (105, 121) | 112 (110, 122) | 118 (103, 126) | 116 (110, 120) | 112 (108, 120) |
| After 1 hour | 120 (110, 125) | | 118 (110, 121) | | 120 (113, 120) | 120 (117, 125) | 120 (110, 126) | 120 (110, 120) | 122 (118, 126) |
| After 2 hours | 120 (110, 125) | | 120 (110, 122) | | 110 (110, 120) | 120 (120, 126) | 119 (110, 125) | 120 (110, 120) | 124 (119, 126) |
| After 3 hours | 120 (110, 125) | | 120 (110, 125) | | 112 (110, 120) | 122 (114, 125) | 120 (110, 124) | 120 (110, 125) | 121 (115, 125) |
| **Diastolic Blood Pressure** | | | | | |  |  |  |  |
| Before IMP | 78 (70, 80) | | 80 (72, 80) | | 75 (70, 80) | 77 (72, 80) | 73 (70, 83) | 80 (70, 80) | 77 (74, 80) |
| After 1 hour | 72 (70, 82) | | 70 (67, 76) | | 72 (70, 80) | 80 (70, 85) | 70 (70, 76) | 70 (70, 80) | 78 (70, 83) |
| After 2 hours | 75 (70, 82) | | 70 (68, 76) | | 72 (70, 80) | 80 (70, 82) | 73 (70, 79) | 75 (70, 80) | 80 (72, 85) |
| After 3 hours | 78 (70, 84) | | 70 (69, 80) | | 71 (70, 80) | 82 (74, 85) | 77 (70, 83) | 76 (70, 81) | 80 (73, 84) |
| **PO2 Saturation** | | | |  | |  |  |  |  |
| Before IMP | 98 (97, 98) | | 98 (97, 99) | | 98 (98, 99) | 97 (97, 98) | 98 (97, 98) | 98 (97, 98) | 98 (97, 98) |
| After 1 hour | 98 (97, 98) | | 98 (97, 98) | | 97 (97, 98) | 98 (97, 98) | 98 (98, 98) | 98 (97, 98) | 98 (97, 98) |
| After 2 hours | 98 (97, 98) | | 98 (97, 98) | | 98 (97, 98) | 98 (97, 98) | 97 (97, 98) | 98 (97, 98) | 98 (97, 99) |
| After 3 hours | 97 (97, 98) | | 98 (98, 98) | | 97 (97, 98) | 97 (97, 98) | 98 (97, 98) | 98 (97, 98) | 97 (97, 98) |
| **Heart Rate** | |  | |  | |  |  |  |  |
| Before IMP | 82 (77, 89) | | 80 (75, 83) | | 82 (78, 91) | 80 (76, 87) | 85 (79, 90) | 85 (78, 90) | 82 (78, 90) |
| After 1 hour | 82 (79, 86) | | 82 (80, 86) | | 83 (80, 89) | 82 (80, 86) | 83 (80, 86) | 80 (78, 84) | 82 (78, 88) |
| After 2 hours | 82 (80, 86) | | 82 (80, 88) | | 82 (80, 84) | 86 (84, 88) | 82 (82, 85) | 82 (76, 83) | 83 (79, 86) |
| After 3 hours | 84 (80, 87) | | 85 (81, 87) | | 84 (82, 86) | 86 (82, 88) | 82 (79, 86) | 80 (76, 82) | 85 (82, 88) |
| **Respiratory Rate** | | | |  | |  |  |  |  |
| Before IMP | 18 (17, 19) | | 18 (17, 19) | | 17 (17, 19) | 18 (16, 19) | 18 (18, 20) | 17 (17, 19) | 18 (17, 19) |
| After 1 hour | 17 (17, 18) | | 17 (17, 18) | | 17 (16, 18) | 18 (17, 20) | 18 (16, 19) | 17 (17, 17) | 18 (17, 18) |
| After 2 hours | 17 (16, 18) | | 18 (17, 18) | | 17 (16, 18) | 17 (16, 19) | 18 (16, 18) | 17 (16, 18) | 18 (17, 19) |
| After 3 hours | 18 (16, 19) | | 17 (16, 18) | | 18 (16, 18) | 18 (16, 19) | 18 (16, 18) | 17 (17, 18) | 18 (17, 19) |
| Median (IQR) | | | | | | | | | |

Table 9. Grading of abnormal vital signs at the time of 1^st^ injection in two administration schedules and different vaccine strengths of 0.5 × 10^6^ TCID_50_ (5 µg/dose), 2.5 × 10^6^ TCID_50_ (10 µg/dose) and placebo

|  | |  | **Schedule 0-14** | | | **Schedule 0-21** | | |  |
| --- | --- | --- | --- | --- | --- | --- | --- | --- | --- |
| **Characteristic** | |  | **Placebo, N = 12** | **Strength 0.5, N = 24** | **Strength 2.5, N = 24** | **Placebo, N = 12** | **Strength 0.5, N = 24** | **Strength 2.5, N = 24** |  |
| **Body temperature(°C)** | |  |  |  |  |  |  |  |  |
| Grade 1 | |  | 0 | 0 | 0 | 0 | 0 | 0 |  |
| Grade 2 | |  | 0 | 0 | 0 | 0 | 0 | 0 |  |
| **Heart rate (Per minute)** | |  |  |  |  |  |  |  |  |
| Grade 1 | |  | 0 | 0 | 0 | 0 | 0 | 0 |  |
| Grade 2 | |  | 0 | 0 | 0 | 0 | 0 | 0 |  |
| **Respiration rate (Per minute)** | |  |  |  |  |  |  |  |  |
| Grade 1 | |  | 1 (8.3%) | 3 (12%) | 2 (8.3%) | 1 (8.3%) | 1 (4.2%) | 2 (8.3%) |  |
| Grade 2 | |  | 0 | 0 | 0 | 0 | 0 | 0 |  |
| **Systolic blood pressure(mmHg)** | | |  |  |  |  |  |  |  |
| Grade 1 | |  | 0 | 0 | 0 | 0 | 0 | 0 |  |
| Grade 2 | |  | 0 | 0 | 0 | 0 | 0 | 0 |  |
| **Diastolic blood pressure(mmHg)** | | |  |  |  |  |  |  |  |
| Grade 1 | |  | 0 | 0 | 0 | 0 | 0 | 0 |  |
| Grade 2 | |  | 0 | 0 | 0 | 0 | 0 | 0 |  |
|  | n (%) | | | | | | | | |

### Second injection

Table 10. Vital signs at the time of 2^nd^ injection in two administration schedules and different vaccine strengths of 0.5 × 10^6^ TCID_50_ (5 µg/dose), 2.5 × 10^6^ TCID_50_ (10 µg/dose) and placebo

|  | **Schedule 0-14** | | | **Schedule 0-21** | | |
| --- | --- | --- | --- | --- | --- | --- |
| **Characteristic** | **Placebo, N = 11** | **Strength 0.5, N = 23** | **Strength 2.5, N = 23** | **Placebo, N = 10** | **Strength 0.5, N = 24** | **Strength 2.5, N = 23** |
| **Body Temperature** | |  |  |  |  |  |
| Before IMP | 36.70 (36.50, 36.70) | 36.60 (36.40, 36.70) | 36.50 (36.50, 36.60) | 36.60 (36.50, 36.60) | 36.50 (36.50, 36.70) | 36.50 (36.40, 36.65) |
| After 1 hour | 36.70 (36.55, 36.80) | 36.50 (36.50, 36.60) | 36.60 (36.45, 36.75) | 36.60 (36.52, 36.70) | 36.55 (36.48, 36.70) | 36.60 (36.50, 36.70) |
| After 2 hours | 36.80 (36.65, 36.80) | 36.60 (36.40, 36.70) | 36.70 (36.65, 36.80) | 36.70 (36.50, 36.80) | 36.70 (36.57, 36.80) | 36.50 (36.45, 36.80) |
| After 3 hours | 36.70 (36.50, 36.80) | 36.50 (36.40, 36.70) | 36.70 (36.65, 36.85) | 36.50 (36.43, 36.70) | 36.70 (36.50, 36.90) | 36.50 (36.50, 36.60) |
| **Systolic Blood Pressure** | | |  |  |  |  |
| Before IMP | 111 (103, 121) | 115 (109, 126) | 112 (106, 118) | 112 (106, 120) | 113 (108, 124) | 111 (106, 124) |
| After 1 hour | 120 (112, 122) | 121 (118, 125) | 118 (110, 121) | 120 (115, 125) | 119 (110, 126) | 111 (105, 126) |
| After 2 hours | 125 (118, 125) | 122 (120, 125) | 122 (115, 128) | 120 (115, 126) | 121 (118, 125) | 116 (110, 124) |
| After 3 hours | 120 (112, 121) | 119 (110, 124) | 120 (110, 125) | 121 (112, 125) | 120 (110, 128) | 115 (110, 123) |
| **Diastolic Blood Pressure** | | |  |  |  |  |
| Before IMP | 74 (70, 79) | 76 (70, 82) | 77 (72, 80) | 72 (68, 80) | 76 (69, 80) | 76 (70, 81) |
| After 1 hour | 72 (70, 74) | 79 (74, 84) | 72 (70, 78) | 74 (70, 79) | 78 (70, 80) | 71 (69, 78) |
| After 2 hours | 72 (70, 75) | 80 (70, 82) | 75 (68, 80) | 72 (70, 80) | 76 (71, 80) | 75 (70, 78) |
| After 3 hours | 70 (70, 74) | 80 (70, 84) | 70 (70, 80) | 72 (69, 79) | 75 (70, 79) | 76 (66, 82) |
| **PO2 Saturation** | | |  |  |  |  |
| Before IMP | 98 (97, 98) | 98 (97, 98) | 97 (96, 98) | 96 (95, 96) | 96 (96, 97) | 96 (96, 97) |
| After 1 hour | 97.00 (97.00, 98.00) | 98.00 (97.00, 98.50) | 98.00 (97.00, 99.00) | 96.50 (96.00, 97.75) | 97.00 (96.00, 98.00) | 97.00 (96.00, 98.00) |
| After 2 hours | 98.00 (97.50, 98.50) | 98.00 (98.00, 98.00) | 98.00 (97.00, 98.00) | 97.00 (96.25, 97.75) | 98.00 (97.00, 99.00) | 97.00 (96.00, 98.00) |
| After 3 hours | 97.00 (97.00, 97.00) | 97.00 (97.00, 98.00) | 97.00 (97.00, 98.00) | 97.00 (96.00, 97.00) | 97.00 (96.00, 98.00) | 97.00 (96.00, 98.00) |
| **Heart Rate** | | |  |  |  |  |
| Before IMP | 80 (72, 84) | 82 (76, 86) | 80 (74, 88) | 79 (69, 86) | 81 (74, 90) | 81 (74, 92) |
| After 1 hour | 82 (80, 84) | 82 (74, 88) | 82 (78, 84) | 82 (77, 85) | 78 (74, 82) | 84 (76, 88) |
| After 2 hours | 86 (82, 88) | 84 (80, 87) | 82 (79, 86) | 84 (78, 88) | 82 (76, 84) | 82 (73, 84) |
| After 3 hours | 82 (81, 82) | 83 (79, 87) | 82 (78, 85) | 81 (74, 84) | 82 (75, 85) | 82 (75, 88) |
| **Respiratory Rate** | | |  |  |  |  |
| Before IMP | 20 (18, 20) | 19 (19, 20) | 20 (19, 20) | 19 (19, 20) | 19 (19, 20) | 19 (19, 20) |
| After 1 hour | 19.00 (17.00, 19.50) | 19.00 (18.00, 20.00) | 19.00 (17.00, 20.00) | 19.00 (17.50, 19.75) | 19.00 (18.50, 20.00) | 19.00 (19.00, 20.00) |
| After 2 hours | 19.00 (17.00, 20.00) | 19.00 (19.00, 20.00) | 18.00 (17.00, 19.50) | 18.50 (18.00, 19.00) | 19.00 (18.00, 20.00) | 19.00 (19.00, 20.00) |
| After 3 hours | 18.00 (18.00, 19.00) | 19.00 (18.00, 20.50) | 18.00 (17.00, 18.50) | 18.00 (17.00, 19.00) | 18.00 (17.75, 20.00) | 19.00 (19.00, 19.00) |
|  | | | | | | |

Table 11. Grading of abnormal vital signs at the time of 2^nd^ injection in two administration schedules and different vaccine strengths of 0.5 × 10^6^ TCID_50_ (5 µg/dose), 2.5 × 10^6^ TCID_50_ (10 µg/dose) and placebo

|  | **Schedule 0-14** | | | **Schedule 0-21** | | |
| --- | --- | --- | --- | --- | --- | --- |
| **Characteristic** | **Placebo, N = 11** | **Strength 0.5, N = 23** | **Strength 2.5, N = 23** | **Placebo, N = 10** | **Strength 0.5, N = 24** | **Strength 2.5, N = 23** |
| **Body temperature(°C)** | | |  |  |  |  |
| Grade 1 | 0 | 0 | 0 | 0 | 0 | 0 |
| Grade 2 | 0 | 0 | 0 | 0 | 0 | 0 |
| **Heart rate(Per minute)** | | | |  |  |  |
| Grade 1 | 0 | 0 | 0 | 0 | 0 | 0 |
| Grade 2 | 0 | 0 | 0 | 0 | 0 | 0 |
| **Respiration rate(Per minute)** | | |  |  |  |  |
| Grade 1 | 1 (9.1%) | 2 (8.7%) | 2 (8.7%) | 0 | 2 (8.3%) | 4 (17%) |
| Grade 2 | 0 | 0 | 0 | 0 | 0 | 0 |
| **Systolic blood pressure(mmHg)** | | |  |  |  |  |
| Grade 1 | 0 | 0 | 0 | 0 | 0 | 0 |
| Grade 2 | 0 | 0 | 0 | 0 | 0 | 0 |
| **Diastolic blood pressure(mmHg)** | | | |  |  |  |
| Grade 1 | 0 | 0 | 0 | 0 | 0 | 0 |
| Grade 2 | 0 | 0 | 0 | 0 | 0 | 0 |
| n (%) | | | | | | |

## Local adverse reactions

### First injection

Figure 2. Proportion of participants experiencing local adverse reactions in the six days following the 1^st^ injection in two administration schedules and different vaccine strengths of 0.5 × 10^6^ TCID_50_ (5 µg/dose), 2.5 × 10^6^ TCID_50_ (10 µg/dose) and placebo


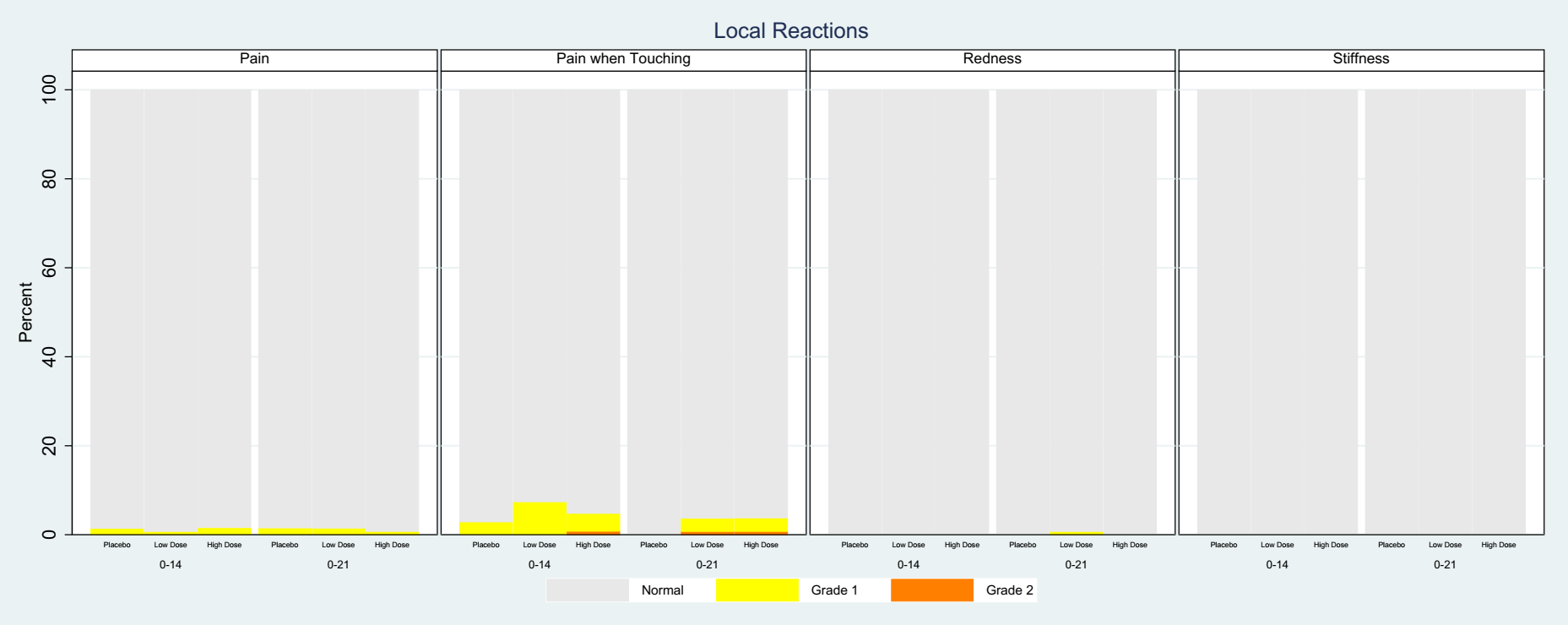


Table 12. Grades of local adverse reactions in the six days following the 1^st^ injection in two administration schedules and different vaccine strengths of 0.5 × 10^6^ TCID_50_ (5 µg/dose), 2.5 × 10^6^ TCID_50_ (10 µg/dose) and placebo

|  | **Schedule 0-14** | | | **Schedule 0-21** | | |
| --- | --- | --- | --- | --- | --- | --- |
| **Characteristic** | **Placebo, N = 12** | **Strength 0.5, N = 24** | **Strength 2.5, N = 24** | **Placebo, N = 12** | **Strength 0.5, N = 24** | **Strength 2.5, N = 24** |
| **Pain** |  |  |  |  |  |  |
| Grade 1 | 1 (1.4%) | 1 (0.7%) | 2 (1.6%) | 1 (1.5%) | 2 (1.5%) | 1 (0.8%) |
| Grade 2 | 0 | 0 | 0 | 0 | 0 | 0 |
| **Pain when touching** | | |  |  |  |  |
| Grade 1 | 2 (2.9%) | 10 (7.4%) | 5 (4.0%) | 0 | 4 (3.0%) | 4 (3.0%) |
| Grade 2 | 0 | 0 | 1 (0.8%) | 0 | 1 (0.7%) | 1 (0.8%) |
| **Redness** |  |  |  |  |  |  |
| Grade 1 | 0 | 0 | 0 | 0 | 1 (0.7%) | 0 |
| Grade 2 | 0 | 0 | 0 | 0 | 0 | 0 |
| **Stiffness** |  |  |  |  |  |  |
| Grade 1 | 0 | 0 | 0 | 0 | 0 | 0 |
| Grade 2 | 0 | 0 | 0 | 0 | 0 | 0 |
| n (%) | | | | | | |

### Second injection

Figure 3. Proportion of participants experiencing local adverse reactions in the six days following the 2^nd^ injection in two administration schedules and different vaccine strengths of 0.5 × 10^6^ TCID_50_ (5 µg/dose), 2.5 × 10^6^ TCID_50_ (10 µg/dose) and placebo


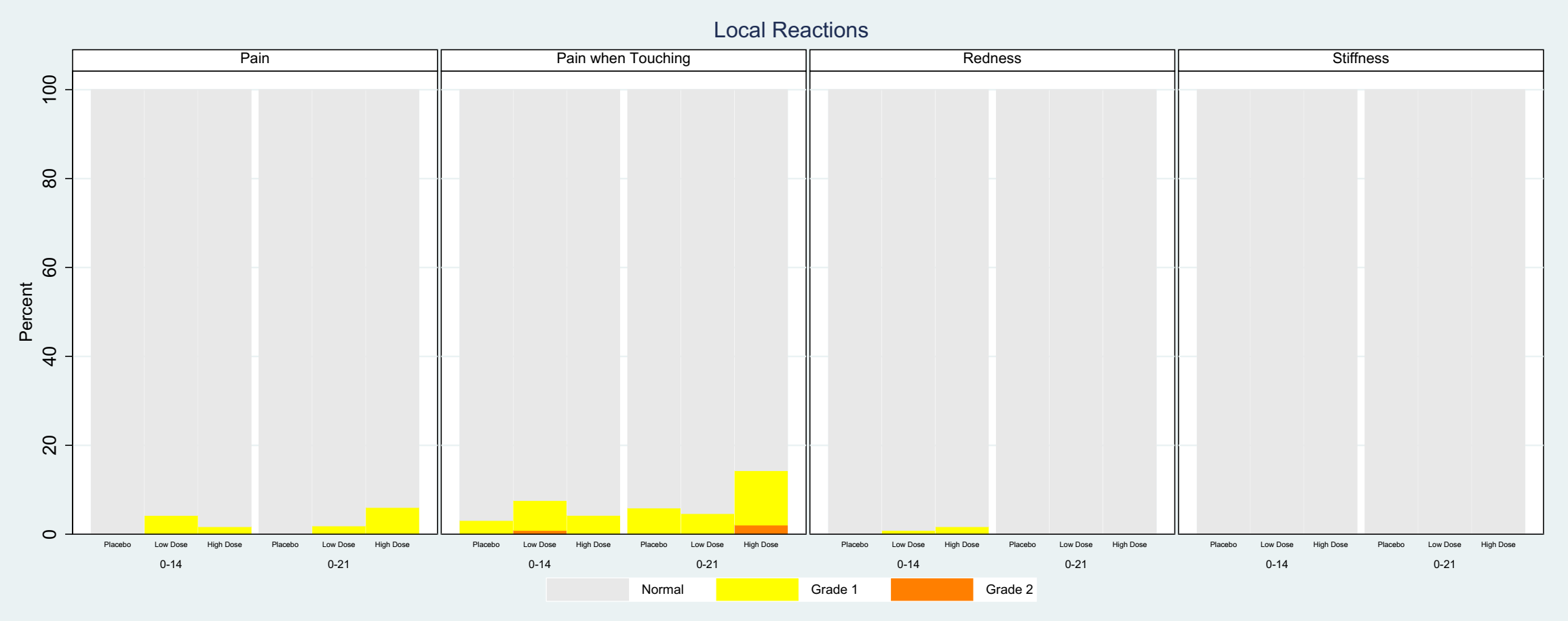


Table 13. Grades of local adverse reactions in the six days following the 2^nd^ injection in two administration schedules and different vaccine strengths of 0.5 × 10^6^ TCID_50_ (5 µg/dose), 2.5 × 10^6^ TCID_50_ (10 µg/dose) and placebo

|  | **Schedule 0-14** | | | **Schedule 0-21** | | |
| --- | --- | --- | --- | --- | --- | --- |
| **Characteristic** | **Placebo, N = 11** | **Strength 0.5, N = 23** | **Strength 2.5, N = 23** | **Placebo, N = 10** | **Strength 0.5, N = 24** | **Strength 2.5, N = 23** |
| **Pain** | |  |  |  |  |  |
| Grade 1 | 0 (0%) | 5 (4.2%) | 2 (1.7%) | 1 (2.0%) | 2 (1.7%) | 4 (3.2%) |
| Grade 2 | 0 (0%) | 0 (0%) | 0 (0%) | 0 (0%) | 0 (0%) | 0 (0%) |
| **Pain when touching** | | |  |  |  |  |
| Grade 1 | 2 (3.1%) | 8 (6.7%) | 5 (4.2%) | 1 (2.0%) | 5 (4.2%) | 12 (9.7%) |
| Grade 2 | 0 (0%) | 1 (0.8%) | 0 (0%) | 0 (0%) | 0 (0%) | 2 (1.6%) |
| **Redness** | | |  |  |  |  |
| Grade 1 | 0 (0%) | 1 (0.8%) | 2 (1.7%) | 0 (0%) | 0 (0%) | 0 (0%) |
| Grade 2 | 0 (0%) | 0 (0%) | 0 (0%) | 0 (0%) | 0 (0%) | 0 (0%) |
| Stiffnes |  |  |  |  |  |  |
| Grade 1 | 0 (0%) | 1 (0.8%) | 0 (0%) | 0 (0%) | 2 (1.7%) | 1 (0.8%) |
| Grade 2 | 0 (0%) | 0 (0%) | 0 (0%) | 0 (0%) | 0 (0%) | 0 (0%) |
| n (%) | | | | | | |

## Systemic adverse reactions

### First injection

Figure 4. Proportion of participants experiencing systemic adverse reactions in the six days following the 1^st^ injection in two administration schedules and different vaccine strengths of 0.5 × 10^6^ TCID_50_ (5 µg/dose), 2.5 × 10^6^ TCID_50_ (10 µg/dose) and placebo


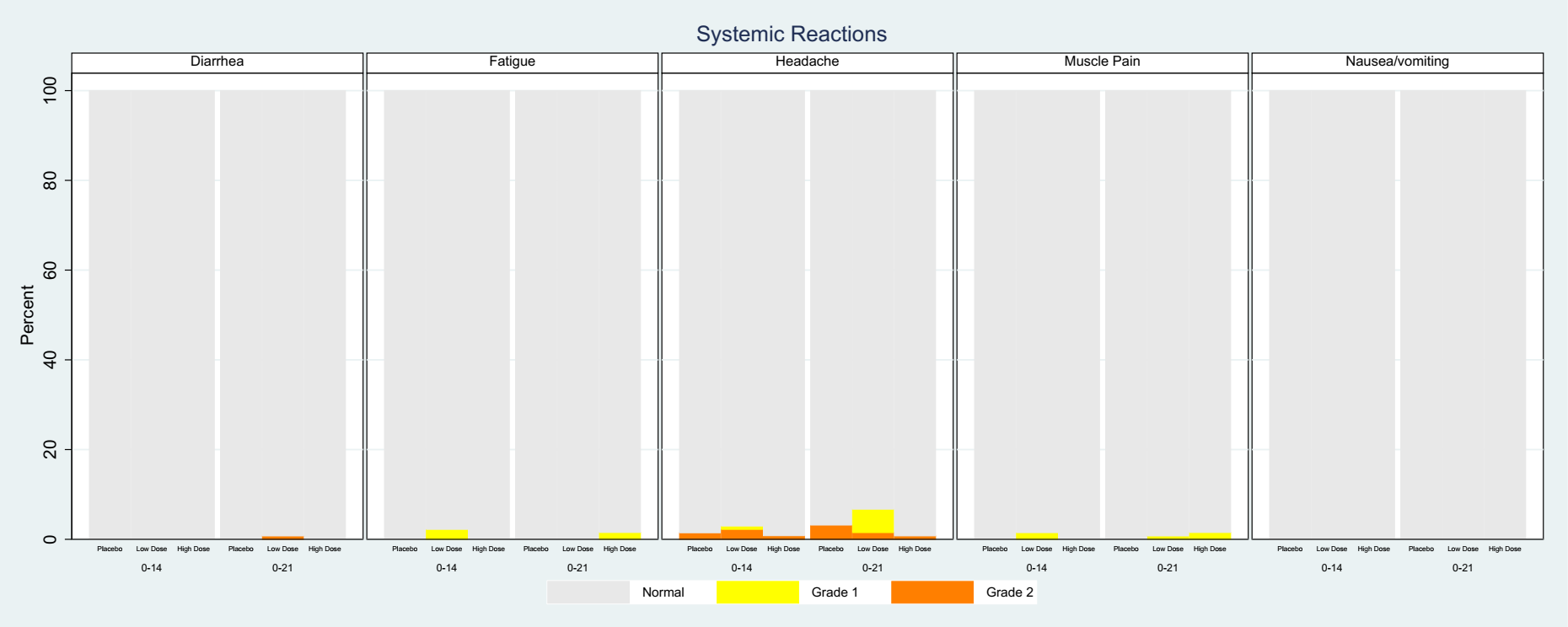


Table 14. Grades of systemic adverse reactions in the six days following the 1^st^ injection in two administration schedules and different vaccine strengths of 0.5 × 10^6^ TCID_50_ (5 µg/dose), 2.5 × 10^6^ TCID_50_ (10 µg/dose) and placebo

|  | **Schedule 0-14** | | | | **Schedule 0-21** | | |  |
| --- | --- | --- | --- | --- | --- | --- | --- | --- |
| **Characteristic** | **Placebo, N = 12** | **Strength 0.5, N = 24** | | **Strength 2.5, N = 24** | **Placebo, N = 12** | **Strength 0.5, N = 24** | **Strength 2.5, N = 24** | |
| **Nausea** |  |  |  | |  |  |  |  |
| Grade 1 | 0 | 0 | 0 | | 0 | 0 | 0 |  |
| Grade 2 | 0 | 0 | 0 | | 0 | 0 | 0 |  |
| **Diarrhea** |  |  |  | |  |  |  |  |
| Grade 1 | 0 | 0 | 0 | | 0 | 0 | 0 |  |
| Grade 2 | 0 | 0 | 0 | | 0 | 1 (0.7%) | 0 |  |
| **Headache** |  |  |  | |  |  |  |  |
| Grade 1 | 0 | 1 (0.7%) | 0 | | 0 | 7 (5.2%) | 0 |  |
| Grade 2 | 1 (1.4%) | 3 (2.2%) | 1 (0.8%) | | 2 (3.2%) | 2 (1.5%) | 1 (0.8%) |  |
| **Fatigue** |  |  |  | |  |  |  |  |
| Grade 1 | 0 | 3 (2.2%) | 0 | | 0 | 0 | 2 (1.5%) |  |
| Grade 2 | 0 | 0 | 0 | | 0 | 0 | 0 |  |
| **Muscular pain** |  |  |  | |  |  |  |  |
| Grade 1 | 0 | 2 (1.5%) | 0 | | 0 | 1 (0.7%) | 2 (1.5%) |  |
| Grade 2 | 0 | 0 | 0 | | 0 | 0 | 0 |  |
| **Vaccine-induced clinical** | |  |  | |  |  |  |  |
| Grade 1 | 1 (1.4%) | 1 (0.7%) | 0 | | 1 (1.6%) | 2 (1.5%) | 0 |  |
| Grade 2 | 0 | 2 (1.5%) | 0 | | 0 | 0 | 0 |  |

### Second injection

Figure 5. Proportion of participants experiencing systemic adverse reactions in the six days following the 2^nd^ injection in two administration schedules and different vaccine strengths of 0.5 × 10^6^ TCID_50_ (5 µg/dose), 2.5 × 10^6^ TCID_50_ (10 µg/dose) and placebo


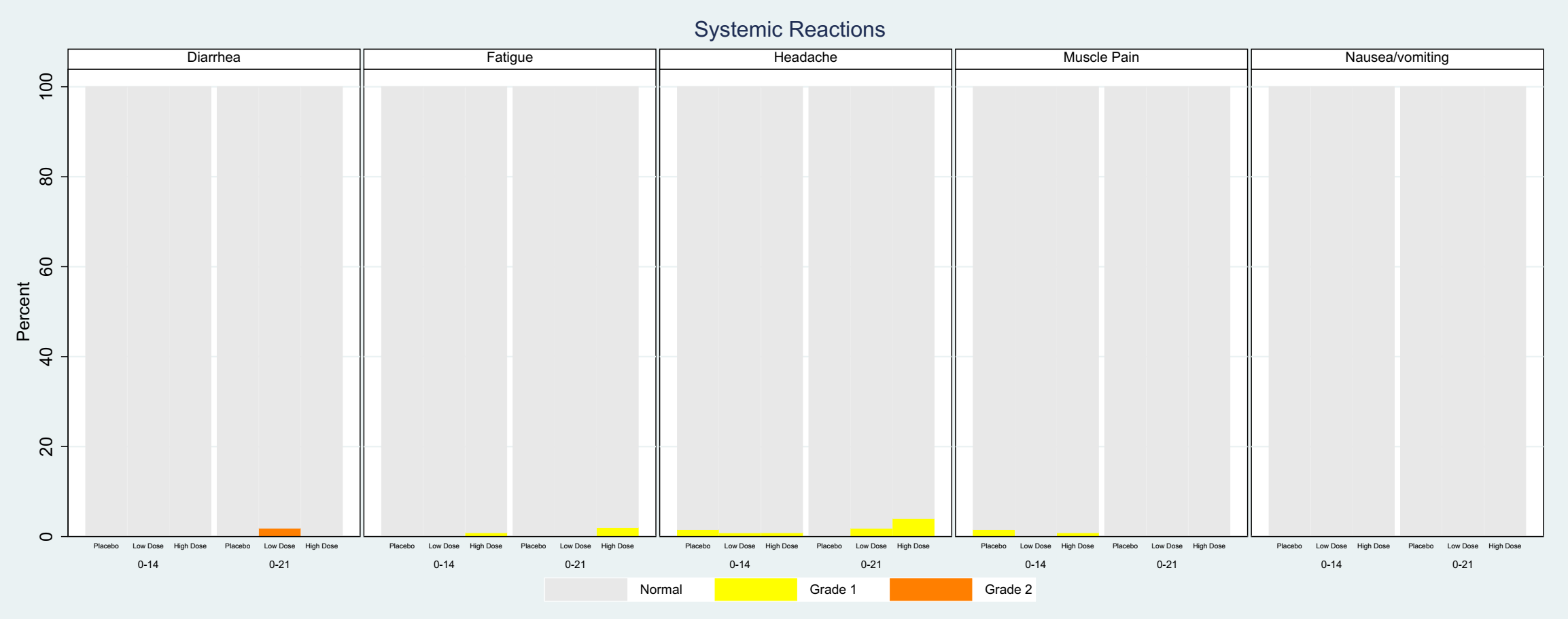


Table 15. Grades of systemic adverse reactions in the six days following the 2^nd^ injection in two administration schedules and different vaccine strengths of 0.5 × 10^6^ TCID_50_ (5 µg/dose), 2.5 × 10^6^ TCID_50_ (10 µg/dose) and placebo

|  | **Schedule 0-14** | | | **Schedule 0-21** | | |
| --- | --- | --- | --- | --- | --- | --- |
| **Characteristic** | **Placebo, N = 11** | **Strength 0.5, N = 23** | **Strength 2.5, N = 23** | **Placebo, N = 10** | **Strength 0.5, N = 24** | **Strength 2.5, N = 23** |
| **Nausea** |  |  |  |  |  |  |
| Grade 1 | 0 (0%) | 0 (0%) | 0 (0%) | 0 (0%) | 0 (0%) | 0 (0%) |
| Grade 2 | 0 (0%) | 0 (0%) | 0 (0%) | 0 (0%) | 0 (0%) | 0 (0%) |
| **Diarrhea** |  |  |  |  |  |  |
| Grade 1 | 0 (0%) | 0 (0%) | 0 (0%) | 0 (0%) | 0 (0%) | 0 (0%) |
| Grade 2 | 0 (0%) | 0 (0%) | 0 (0%) | 0 (0%) | 2 (2.0%) | 0 (0%) |
| **Headache** |  |  |  |  |  |  |
| Grade 1 | 1 (1.5%) | 1 (0.8%) | 1 (0.9%) | 0 (0%) | 1 (1.0%) | 2 (1.9%) |
| Grade 2 | 0 (0%) | 0 (0%) | 0 (0%) | 0 (0%) | 0 (0%) | 1 (1.0%) |
| **Fatigue** |  |  |  |  |  |  |
| Grade 1 | 0 (0%) | 0 (0%) | 1 (0.9%) | 0 (0%) | 0 (0%) | 1 (1.0%) |
| Grade 2 | 0 (0%) | 0 (0%) | 0 (0%) | 0 (0%) | 0 (0%) | 0 (0%) |
| **Muscular pain** |  |  |  |  |  |  |
| Grade 1 | 1 (1.5%) | 0 (0%) | 1 (0.9%) | 0 (0%) | 0 (0%) | 0 (0%) |
| Grade 2 | 0 (0%) | 0 (0%) | 0 (0%) | 0 (0%) | 0 (0%) | 0 (0%) |
| **Vaccine-induced clinical** |  |  |  |  |  |  |
| Grade 1 | 0 (0%) | 0 (0%) | 0 (0%) | 0 (0%) | 0 (0%) | 0 (0%) |
| Grade 2 | 0 (0%) | 0 (0%) | 0 (0%) | 0 (0%) | 0 (0%) | 0 (0%) |

## Laboratory findings

Table 16. Median and interquartile range (IQR) of laboratory findings at screening day and one week after each injection in two administration schedules and different vaccine strengths of 0.5 × 10^6^ TCID_50_ (5 µg/dose), 2.5 × 10^6^ TCID_50_ (10 µg/dose) and placebo

|  | **Schedule 0-14** | | | **Schedule 0-21** | | |
| --- | --- | --- | --- | --- | --- | --- |
| **Characteristic** | **Placebo, N = 12** | **Strength 0.5, N = 24** | **Strength 2.5, N = 24** | **Placebo, N = 12** | **Strength 0.5, N = 24** | **Strength 2.5, N = 24** |
| **Hemoglobin gm/Dl** | | | | | | |
| Screening | 15.15 (14.37, 15.83) | 14.20 (13.58, 15.55) | 15.60 (14.60, 16.70) | 15.60 (15.20, 16.55) | 15.40 (14.47, 16.10) | 14.70 (13.70, 16.13) |
| 7 days after 1^st^ Vaccination | 15.15 (14.28, 16.10) | 13.75 (13.13, 15.08) | 15.60 (14.38, 16.13) | 15.35 (14.77, 15.98) | 15.30 (13.85, 15.60) | 14.80 (13.65, 15.75) |
| 7 days after 2^nd^ Vaccination | 14.00 (13.30, 15.45) | 13.70 (12.85, 14.80) | 15.60 (14.40, 15.80) | 14.80 (14.10, 16.00) | 15.10 (14.40, 15.80) | 14.45 (13.55, 15.45) |
| **WBC cell/mm3** |  |  |  |  |  |  |
| Screening | 6.15 (5.60, 6.60) | 5.44 (5.14, 6.15) | 6.40 (5.88, 7.50) | 6.10 (5.59, 7.31) | 6.20 (5.03, 6.78) | 6.23 (5.68, 6.72) |
| 7 days after 1^st^ Vaccination | 6.30 (5.93, 7.29) | 5.57 (5.00, 6.16) | 6.19 (5.31, 6.76) | 6.31 (5.98, 7.42) | 6.46 (5.35, 6.75) | 5.90 (5.25, 6.59) |
| 7 days after 2^nd^ Vaccination | 5.91 (5.58, 7.25) | 5.28 (5.10, 5.90) | 6.20 (5.57, 6.92) | 6.08 (5.49, 8.01) | 6.20 (5.43, 6.61) | 5.60 (4.78, 6.06) |
| **Lymphocytes cell/mm3** | | | | | | |
| Screening | 1.95 (1.83, 2.11) | 1.79 (1.66, 2.00) | 1.97 (1.81, 2.48) | 1.98 (1.75, 2.12) | 1.83 (1.62, 2.16) | 1.88 (1.62, 2.34) |
| 7 days after 1^st^ Vaccination | 2.34 (1.91, 2.51) | 1.85 (1.69, 2.10) | 2.06 (1.84, 2.60) | 2.03 (1.71, 2.55) | 1.99 (1.65, 2.42) | 1.80 (1.62, 2.25) |
| 7 days after 2^nd^ Vaccination | 2.18 (1.91, 2.59) | 2.01 (1.75, 2.17) | 2.06 (1.82, 2.56) | 1.63 (1.49, 2.18) | 2.06 (1.66, 2.23) | 1.91 (1.60, 2.29) |
| **Neutrophils cell/mm3** | | | | | | |
| Screening | 3.81 (3.11, 4.36) | 3.15 (2.92, 3.75) | 4.02 (3.53, 4.63) | 3.80 (3.25, 4.34) | 3.44 (2.92, 4.25) | 3.65 (3.25, 4.19) |
| 7 days after 1^st^ Vaccination | 3.40 (3.00, 4.24) | 3.15 (2.96, 3.54) | 3.23 (2.60, 3.94) | 3.49 (3.23, 4.87) | 3.56 (2.88, 4.25) | 3.36 (2.76, 3.71) |
| 7 days after 2^nd^ Vaccination | 3.23 (2.75, 3.66) | 2.70 (2.38, 3.27) | 3.32 (2.90, 3.59) | 4.15 (2.95, 5.03) | 3.44 (3.06, 3.74) | 3.01 (2.69, 3.46) |
| **Eosinophils - cell/mm3** | | | | | | |
| Screening | 0.10 (0.09, 0.13) | 0.14 (0.07, 0.19) | 0.17 (0.09, 0.23) | 0.18 (0.12, 0.22) | 0.11 (0.08, 0.19) | 0.15 (0.09, 0.26) |
| 7 days after 1^st^ Vaccination | 0.20 (0.15, 0.26) | 0.16 (0.08, 0.24) | 0.18 (0.10, 0.24) | 0.18 (0.11, 0.30) | 0.16 (0.09, 0.23) | 0.12 (0.08, 0.23) |
| 7 days after 2^nd^ Vaccination | 0.18 (0.11, 0.23) | 0.19 (0.11, 0.26) | 0.21 (0.12, 0.34) | 0.19 (0.15, 0.24) | 0.19 (0.09, 0.33) | 0.18 (0.09, 0.20) |
| **Platelets cell/mm3** |  |  |  |  |  |  |
| Screening | 269 (216, 312) | 234 (204, 287) | 262 (234, 281) | 254 (246, 284) | 238 (211, 289) | 238 (204, 277) |
| 7 days after 1^st^ Vaccination | 258 (218, 304) | 233 (187, 262) | 244 (228, 286) | 266 (251, 268) | 253 (205, 268) | 244 (193, 278) |
| 7 days after 2^nd^ Vaccination | 246 (184, 288) | 234 (189, 254) | 260 (239, 287) | 259 (236, 298) | 238 (213, 280) | 237 (204, 265) |
| **BUN mg/dL** |  |  |  |  |  |  |
| Screening | 13.20 (10.62, 15.15) | 12.05 (10.47, 15.15) | 13.25 (10.87, 16.18) | 11.5 (10.3, 12.3) | 11.1 (9.3, 15.5) | 12.2 (11.0, 15.6) |
| 7 days after 1^st^ Vaccination | 13.85 (11.30, 15.13) | 13.55 (12.13, 14.48) | 15.00 (12.78, 16.50) | 11.9 (10.0, 13.7) | 12.4 (9.9, 13.5) | 15.4 (11.8, 16.9) |
| 7 days after 2^nd^ Vaccination | 12.70 (12.10, 13.70) | 13.80 (13.00, 15.55) | 13.60 (11.80, 15.60) | 12.6 (10.8, 13.5) | 11.8 (10.7, 15.2) | 13.4 (12.1, 15.6) |
| **Creatinine – mg/dL** | | | |  |  |  |
| Screening | 0.94 (0.82, 1.08) | 0.93 (0.81, 1.03) | 1.00 (0.84, 1.14) | 1.05 (0.82, 1.16) | 0.97 (0.92, 1.06) | 1.00 (0.90, 1.13) |
| 7 days after 1^st^ Vaccination | 0.97 (0.93, 1.11) | 0.94 (0.81, 1.07) | 1.05 (0.90, 1.20) | 1.04 (0.91, 1.19) | 0.98 (0.94, 1.06) | 1.07 (0.87, 1.12) |
| 7 days after 2^nd^ Vaccination | 0.96 (0.77, 1.08) | 0.98 (0.80, 1.07) | 1.02 (0.91, 1.13) | 0.87 (0.86, 0.99) | 1.03 (0.95, 1.06) | 1.05 (0.90, 1.13) |
| **AST IU/L** |  |  |  |  |  |  |
| Screening | 16.5 (14.8, 20.2) | 17.0 (14.0, 20.0) | 18.0 (16.8, 21.0) | 17.5 (14.5, 19.5) | 17.0 (16.0, 21.0) | 16.0 (14.0, 18.2) |
| 7 days after 1^st^ Vaccination | 16 (13, 20) | 15 (14, 18) | 17 (15, 19) | 18 (13, 22) | 18 (14, 19) | 15 (14, 18) |
| 7 days after 2^nd^ Vaccination | 13 (11, 18) | 15 (12, 16) | 18 (14, 19) | 14 (12, 20) | 16 (12, 18) | 15 (14, 18) |
| **ALT IU/L** |  |  |  |  |  |  |
| Screening | 16 (10, 22) | 16 (11, 20) | 20 (15, 30) | 20 (18, 24) | 18 (15, 26) | 14 (11, 24) |
| 7 days after 1^st^ Vaccination | 14 (10, 20) | 14 (12, 19) | 17 (12, 25) | 18 (16, 26) | 23 (15, 27) | 17 (12, 20) |
| 7 days after 2^nd^ Vaccination | 10 (10, 16) | 11 (10, 18) | 21 (13, 32) | 17 (13, 20) | 21 (15, 30) | 18 (12, 24) |
| **Alkaline phosphatase IU/L** | | | |  |  |  |
| Screening | 142 (126, 159) | 138 (118, 154) | 166 (154, 210) | 182 (139, 203) | 162 (128, 180) | 128 (108, 162) |
| 7 days after 1^st^ Vaccination | 138 (124, 172) | 133 (116, 156) | 158 (148, 205) | 188 (159, 214) | 157 (128, 170) | 130 (115, 176) |
| 7 days after 2^nd^ Vaccination | 137 (114, 155) | 132 (120, 148) | 170 (154, 208) | 157 (124, 198) | 152 (133, 172) | 123 (102, 142) |
| **Bilirubin total** |  |  |  |  |  |  |
| Screening | 0.72 (0.57, 0.84) | 0.58 (0.40, 0.74) | 0.62 (0.52, 0.86) | 0.62 (0.50, 0.79) | 0.54 (0.46, 0.73) | 0.55 (0.46, 0.64) |
| 7 days after 1^st^ Vaccination | 0.81 (0.65, 1.00) | 0.53 (0.43, 0.71) | 0.56 (0.46, 0.67) | 0.54 (0.38, 0.76) | 0.53 (0.48, 0.69) | 0.58 (0.54, 0.86) |
| 7 days after 2^nd^ Vaccination | 0.70 (0.52, 0.80) | 0.46 (0.39, 0.66) | 0.61 (0.45, 0.64) | 0.58 (0.39, 0.62) | 0.46 (0.41, 0.75) | 0.67 (0.53, 0.82) |
| **Sodium, mEq/L** |  |  |  |  |  |  |
| Screening | 139.5 (139, 140) | 140 (139, 141) | 140 (139, 140) | 140 (139.75, 141.25) | 141 (140, 142) | 141 (139, 141) |
| 7 days after 1^st^ Vaccination | 140 (139, 140) | 140 (139, 141) | 140 (139, 140) | 140 (139, 141) | 140 (140, 141) | 140 (140, 141) |
| 7 days after 2^nd^ Vaccination | 139 (138, 140) | 139 (139, 140) | 139 (138, 140) | 139 (138, 140) | 139 (139, 140) | 140 (139, 140) |

### First injection

Table 17. Grading of abnormal laboratory findings one week after 1^st^ injection in two administration schedules and different vaccine strengths of 0.5 × 10^6^ TCID_50_ (5 µg/dose), 2.5 × 10^6^ TCID_50_ (10 µg/dose) and placebo

|  | **Schedule 0-14** | | | **Schedule 0-21** | | |
| --- | --- | --- | --- | --- | --- | --- |
| **Characteristic** | **Placebo, N = 12** | **Strength 0.5, N = 24** | **Strength 2.5, N = 24** | **Placebo, N = 12** | **Strength 0.5, N = 24** | **Strength 2.5, N = 24** |
| **Hemoglobin mg/dL** |  |  |  |  |  |  |
| Grade 1 | 0 | 2 (9.09%) | 0 | 0 | 1 (4.35%) | 0 |
| Grade 2 | 0 | 0 | 0 | 0 | 0 | 0 |
| **WBC cell/mm3** |  |  |  |  |  |  |
| Grade 1 | 0 | 0 | 0 | 0 | 0 | 0 |
| Grade 2 | 0 | 0 | 0 | 0 | 0 | 0 |
| **CRP (mg/dL)** |  |  |  |  |  |  |
| Grade 1 | 0 | 0 | 0 | 1 (11.11%) | 0 | 2 (9.09%) |
| Grade 2 | 0 | 0 | 0 | 1(11.11%) | 0 | 0 |
| **Lymphocytes cell/mm3** |  |  |  |  |  |  |
| Grade 1 | 0 | 1 (4.55%) | 0 | 0 | 0 | 1 (4.76%) |
| Grade 2 | 0 | 0 | 0 | 0 | 0 | 0 |
| **Neutrophils cell/mm3** |  |  |  |  |  |  |
| Grade 1 | 0 | 1 (4.55%) | 0 | 0 | 0 | 1 (4.76%) |
| Grade 2 | 0 | 0 | 0 | 0 | 0 | 0 |
| **Eosinophils - cell/mm3** |  |  |  |  |  |  |
| Grade 1 | 0 | 0 | 0 | 1 (11.11%) | 0 | 1 (4.76%) |
| Grade 2 | 0 | 0 | 0 | 0 | 0 | 0 |
| **Platelets cell/mm3** |  |  |  |  |  |  |
| Grade 1 | 0 | 0 | 0 | 0 | 0 | 0 |
| Grade 2 | 0 | 0 | 0 | 0 | 0 | 0 |
| **LDH(U/L)** |  |  |  |  |  |  |
| Grade 1 | 0 | 0 | 0 | 0 | 0 | 0 |
| Grade 2 | 0 | 0 | 0 | 0 | 0 | 0 |
| **BUN mg/dL** |  |  |  |  |  |  |
| Grade 1 | 1 (9.09%) | 1 (4.55%) | 0 | 0 | 0 | 0 |
| Grade 2 | 0 | 0 | 0 | 0 | 0 | 0 |
| **Creatinine - mg/dL** |  |  |  |  |  |  |
| Grade 1 | 0 | 0 | 0 | 0 | 0 | 0 |
| Grade 2 | 0 | 0 | 0 | 0 | 0 | 0 |
| **AST IU/L** |  |  |  |  |  |  |
| Grade 1 | 0 | 0 | 0 | 0 | 0 | 0 |
| Grade 2 | 0 | 0 | 0 | 0 | 0 | 0 |
| **ALT IU/L** |  |  |  |  |  |  |
| Grade 1 | 0 | 0 | 1 (4.55%) | 0 | 1 (4.35%) | 0 |
| Grade 2 | 0 | 0 | 0 | 0 | 0 | 0 |
| **Alkaline phosphatase IU/L** |  |  |  |  |  |  |
| Grade 1 | 0 | 0 | 0 | 0 | 0 | 0 |
| Grade 2 | 0 | 0 | 0 | 0 | 0 | 0 |
| **Bilirubin total** |  |  |  |  |  |  |
| Grade 1 | 1 (9.09%) | 2 (9.09%) | 0 | 0 | 1 (4.35%) | 0 |
| Grade 2 | 0 | 0 | 0 | 0 | 0 | 1 (4.55%) |
| **Potassium, mEq/L** |  |  |  |  |  |  |
| Grade 1 | 0 | 0 | 0 | 0 | 0 | 0 |
| Grade 2 | 0 | 0 | 0 | 0 | 0 | 0 |
| **Sodium, mEq/L** |  |  |  |  |  |  |
| Grade 1 | 0 | 0 | 0 | 0 | 0 | 0 |
| Grade 2 | 0 | 0 | 0 | 0 | 0 | 0 |
| **CPK (mg/dL)** |  |  |  |  |  |  |
| Grade 1 | 0 | 1 (4.55%) | 0 | 0 | 0 | 0 |
| Grade 2 | 0 | 0 | 0 | 0 | 0 | 0 |
| **ESR, mm/hr** |  |  |  |  |  |  |
| Grade 1 | 0 | 1 (4.55%) | 0 | 0 | 0 | 0 |
| Grade 2 | 0 | 0 | 0 | 0 | 0 | 0 |
| **U/A,Urine glucose** |  |  |  |  |  |  |
| Grade 1 | 0 | 0 | 0 | 0 | 0 | 0 |
| Grade 2 | 0 | 0 | 0 | 0 | 0 | 0 |
| **U/A,Urine protein** |  |  |  |  |  |  |
| Grade 1 | 1 (9.09%) | 2 (9.09%) | 2 (9.09%) | 0 | 0 | 1 (4.55%) |
| Grade 2 | 1 (9.09%) | 0 | 0 | 0 | 0 | 0 |
| **U/A,RBC** |  |  |  |  |  |  |
| Grade 1 | 1 (10%) | 0 | 2 (9.09%) | 0 | 0 | 1 (4.55%) |
| Grade 2 | 0 | 1 (4.55%) | 0 | 0 | 0 | 0 |
| n (%) | | | | | | |

### Second injection

Table 18. Grading of abnormal laboratory findings one week after 2^nd^ injection in two administration schedules and different vaccine strengths of 0.5 × 10^6^ TCID_50_ (5 µg/dose), 2.5 × 10^6^ TCID_50_ (10 µg/dose) and placebo

|  | **Schedule 0-14** | | | | | | **Schedule 0-21** | | | | |
| --- | --- | --- | --- | --- | --- | --- | --- | --- | --- | --- | --- |
| **Characteristic** | **Placebo, N = 11** | | **Strength 0.5, N = 23** | | **Strength 2.5, N = 23** | | **Placebo, N = 10** | | **Strength 0.5, N = 24** | | **Strength 2.5, N = 23** |
| **Hemoglobin mg/Dl** |  | |  | |  | |  | |  | |  |
| Grade 1 | 1 (9.09%) | | 2 (8.70%) | | 0 | | 0 | | 2 (10%) | | 0 |
| Grade 2 | 0 | | 0 | | 0 | | 0 | | 0 | | 0 |
| **WBC cell/mm3** |  | |  | |  | |  | |  | |  |
| Grade 1 | 0 | | 0 | | 0 | | 0 | | 0 | | 0 |
| Grade 2 | 0 | | 0 | | 0 | | 0 | | 0 | | 0 |
| **Lymphocytes cell/mm3** |  | |  | |  | |  | |  | |  |
| Grade 1 | 0 | | 1 (4.35%) | | 0 | | 0 | | 0 | | 0 |
| Grade 2 | 0 | | 0 | | 0 | | 0 | | 0 | | 0 |
| Grade 3 | 0 | | 0 | | 0 | | 0 | | 0 | | 1 (5%) |
| **Neutrophils cell/mm3** |  | |  | |  | |  | |  | |  |
| Grade 1 | 1 (10%) | | 2 (8.70%) | | 1 (4.55%) | | 0 | | 1 (5%) | | 2 (10%) |
| Grade 2 | 0 | | 0 | | 0 | | 0 | | 0 | | 0 |
| **Platelets cell/mm3** |  |  | |  | |  | |  | |  | |
| Grade 1 | 0 | 0 | | 0 | | 0 | | 0 | | 0 | |
| Grade 2 | 0 | 0 | | 0 | | 0 | | 0 | | 0 | |
| **Eosinophils - cell/mm3** |  | |  | |  | |  | |  | |  |
| Grade 1 | 0 | | 1 (4.35%) | | 1 (4.55%) | | 1 (11.11%) | | 0 | | 0 |
| Grade 2 | 0 | | 0 | | 0 | | 0 | | 0 | | 0 |
| **CRP (mg/dL)** |  | |  | |  | |  | |  | |  |
| Grade 1 | 0 | | 0 | | 0 | | 0 | | 0 | | 0 |
| Grade 2 | 0 | | 0 | | 0 | | 0 | | 0 | | 1 (5%) |
| **BUN mg/dL** |  | |  | |  | |  | |  | |  |
| Grade 1 | 1 (10%) | | 0 | | 0 | | 0 | | 0 | | 0 |
| Grade 2 | 0 | | 0 | | 0 | | 0 | | 0 | | 0 |
| **Creatinine - mg/dL** |  | |  | |  | |  | |  | |  |
| Grade 1 | 0 | | 0 | | 0 | | 0 | | 0 | | 0 |
| Grade 2 | 0 | | 0 | | 0 | | 0 | | 0 | | 0 |
| **AST IU/L** |  | |  | |  | |  | |  | |  |
| Grade 1 | 0 | | 1 (4.35%) | | 0 | | 0 | | 1 (5%) | | 0 |
| Grade 2 | 0 | | 0 | | 0 | | 0 | | 0 | | 0 |
| **ALT IU/L** |  | |  | |  | |  | |  | |  |
| Grade 1 | 0 | | 1 (4.35%) | | 1 (4.55%) | | 0 | | 1 (5%) | | 1 (5%) |
| Grade 2 | 0 | | 0 | | 0 | | 0 | | 0 | | 0 |
| **Alkaline phosphatase IU/L** | | |  | |  | |  | |  | |  |
| Grade 1 | 0 | | 0 | | 0 | | 0 | | 0 | | 0 |
| Grade 2 | 0 | | 0 | | 0 | | 0 | | 0 | | 0 |
| **Bilirubin total** |  | |  | |  | |  | |  | |  |
| Grade 1 | 0 | | 1 (4.35%) | | 1 (4.55%) | | 0 | | 0 | | 0 |
| Grade 2 | 0 | | 0 | | 0 | | 0 | | 0 | | 0 |
| **Potassium, mEq/L** |  | |  | |  | |  | |  | |  |
| Grade 1 | 0 | | 1 (4.55%) | | 0 | | 0 | | 0 | | 0 |
| Grade 2 | 0 | | 0 | | 0 | | 0 | | 0 | | 0 |
| **Sodium, mEq/L** |  | |  | |  | |  | |  | |  |
| Grade 1 | 0 | | 0 | | 0 | | 0 | | 0 | | 0 |
| Grade 2 | 0 | | 0 | | 0 | | 0 | | 0 | | 0 |
| **U/A,Urine glucose** |  | |  | |  | |  | |  | |  |
| Grade 1 | 0 | | 0 | | 0 | | 0 | | 0 | | 0 |
| Grade 2 | 0 | | 0 | | 0 | | 0 | | 0 | | 0 |
| **ESR, mm/hr** |  | |  | |  | |  | |  | |  |
| Grade 1 | 0 | | 0 | | 0 | | 0 | | 0 | | 0 |
| Grade 2 | 0 | | 0 | | 0 | | 0 | | 0 | | 0 |
| **LDH(U/L)** |  | |  | |  | |  | |  | |  |
| Grade 1 | 0 | | 0 | | 0 | | 0 | | 0 | | 1 (5%) |
| Grade 2 | 0 | | 0 | | 0 | | 0 | | 0 | | 0 |
| **CPK (mg/dL)** |  | |  | |  | |  | |  | |  |
| Grade 1 | 0 | | 0 | | 0 | | 0 | | 0 | | 0 |
| Grade 2 | 0 | | 0 | | 0 | | 0 | | 0 | | 0 |
| **U/A,Urine protein** |  | |  | |  | |  | |  | |  |
| Grade 1 | 1 (9.09%) | | 1 (4.35%) | | 1 (4.55%) | | 0 | | 1 (5%) | | 2 (10%) |
| Grade 2 | 0 | | 0 | | 0 | | 0 | | 0 | | 0 |
| **U/A,RBC** |  | |  | |  | |  | |  | |  |
| Grade 1 | 0 | | 1 (4.35%) | | 0 | | 0 | | 0 | | 0 |
| Grade 2 | 0 | | 0 | | 0 | | 0 | | 0 | | 2 (10.53%) |
| n (%) | | | | | | | | | | | |

# Immunogenicity outcomes

## Neutralizing antibody activity

Table 19. Geometric mean titer, geometric mean ratio (compared to placebo), and geometric mean fold increase (compared to day zero) for serum neutralizing antibody titers and their 95% confidence intervals at predefined time intervals in two administration schedules and different vaccine strengths of 0.5 × 10^6^ TCID_50_ (5 µg/dose), 2.5 × 10^6^ TCID_50_ (10 µg/dose) and placebo (has been included in the main manuscript)

| day | 0 | Injection day | 14 days after 2^nd^ injection | 28 days after 2^nd^ injection |
| --- | --- | --- | --- | --- |
| GMT |  | | | |
| Placebo, 0-14 | 1 (1-1, N: 10) | 1.1 (0.9 - 1.34, N: 12) | 1 (1-1, N: 11) | 1.07 (0.9 - 1.27, N: 6) |
| Strength 0.5, 0-14 | 1 (1-1, N: 24) | 1.56 (1.13 - 2.15, N: 29) | 2.74 (1.76 - 4.28, N: 29) | 3.34 (2.06 - 5.41, N: 25) |
| Strength 2.5, 0-14 | 1.07 (0.93 - 1.23, N: 29) | 2.15 (1.35 - 3.41, N: 30) | 9.03 (5.17 - 15.8, N: 26) | 6.21 (3.32 - 11.64, N: 14) |
| Placebo, 0-21 | 1 (1-1, N: 9) | 1.12 (0.87 - 1.43, N: 10) | 1 (1-1, N: 4) | 1.2 (0.77 - 1.85, N: 7) |
| Strength 0.5, 0-21 | 1 (1-1, N: 19) | 1.78 (1.22 - 2.59, N: 24) | 5.2 (2.8 - 9.65, N: 14) | 3.42 (1.54 - 7.57, N: 12) |
| Strength 2.5, 0-21 | 1 (1-1, N: 21) | 2.39 (1.37 - 4.16, N: 23) | 11.77 (2.29 - 60.52, N: 4) | 4.71 (2.39 - 9.27, N: 16) |
| GMR |  | | | |
| Placebo, 0-14 | 1 | 1 | 1 | 1 |
| Strength 0.5, 0-14 | 1 (0.83-1.2, N:10) | 1.42 (0.72-2.79, N:12) | 2.74 (1.2-6.28, N:11) | 3.12 (1.17-8.35, N:6) |
| Strength 2.5, 0-14 | 1.07 (0.89-1.28, N:24) | 1.96 (1-3.83, N:29) | 9.03 (3.89-20.95, N:29) | 5.81 (2.02-16.69, N:25) |
| Placebo, 0-21 | 1 | 1 | 1 | 1 |
| Strength 0.5, 0-21 | 1 (1-1, N:19) | 1.59 (0.74-3.43, N:24) | 5.2 (1.63-16.55, N:14) | 2.86 (0.93-8.75, N:12) |
| Strength 2.5, 0-21 | 1 (1-1, N:21) | 2.14 (0.99-4.62, N:23) | 11.77 (2.77-49.94, N:4) | 3.94 (1.35-11.45, N:16) |
| GMFI |  | | | |
| Placebo, 0-14 | 1 | 1.12 (0.87 - 1.43, N: 10) | 1 (1-1, N: 9) | 1.11 (0.8 - 1.53, N: 4) |
| Strength 0.5, 0-14 | 1 | 1.51 (1.04 - 2.18, N: 23) | 2.51 (1.54 - 4.07, N: 24) | 3.5 (2.07 - 5.91, N: 20) |
| Strength 2.5, 0-14 | 1 | 2.12 (1.35 - 3.32, N: 28) | 8.13 (4.6 - 14.4, N: 24) | 6.23 (3.14 - 12.34, N: 13) |
| Placebo, 0-21 | 1 | 1.15 (0.83 - 1.59, N: 8) | 1 (1-1, N: 2) | 1.2 (0.77 - 1.85, N: 7) |
| Strength 0.5, 0-21 | 1 | 1.71 (1.1 - 2.67, N: 19) | 5.31 (2.56 - 11.02, N: 12) | 3.42 (1.54 - 7.57, N: 12) |
| Strength 2.5, 0-21 | 1 | 2.35 (1.3 - 4.25, N: 21) | 8.43 (0.78 - 91.56, N: 3) | 4.71 (2.39 - 9.27, N: 16) |

Figure 6. Changes in serum neutralizing antibody titers over the study period for each individual participant and the group mean in two administration schedules and different vaccine strengths of 0.5 × 10^6^ TCID_50_ (5 µg/dose), 2.5 × 10^6^ TCID_50_ (10 µg/dose) and placebo


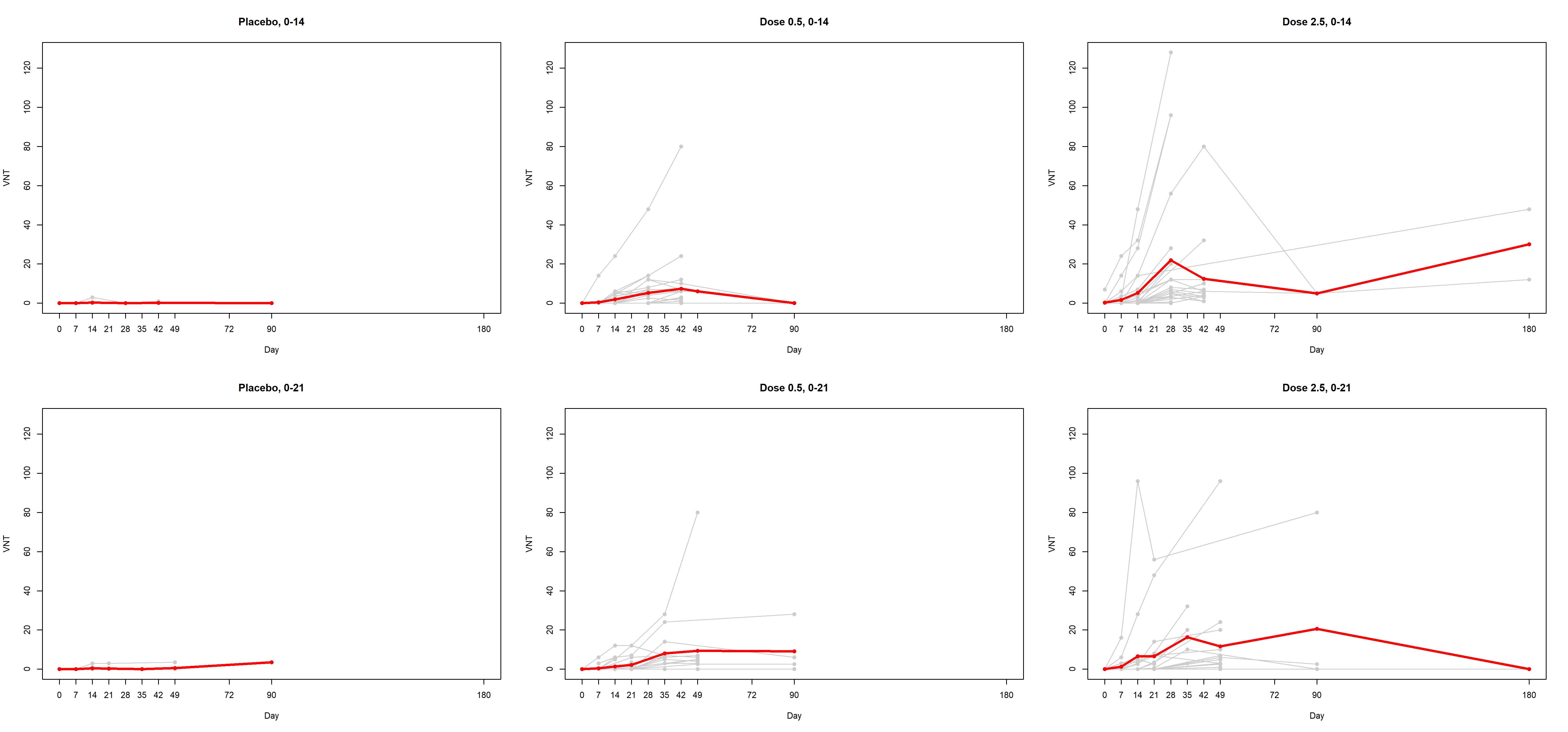


Figure 7. Changes in group means of serum neutralizing antibody titers over the study period in two administration schedules and different vaccine strengths of 0.5 × 10^6^ TCID_50_ (5 µg/dose), 2.5 × 10^6^ TCID_50_ (10 µg/dose) and placebo


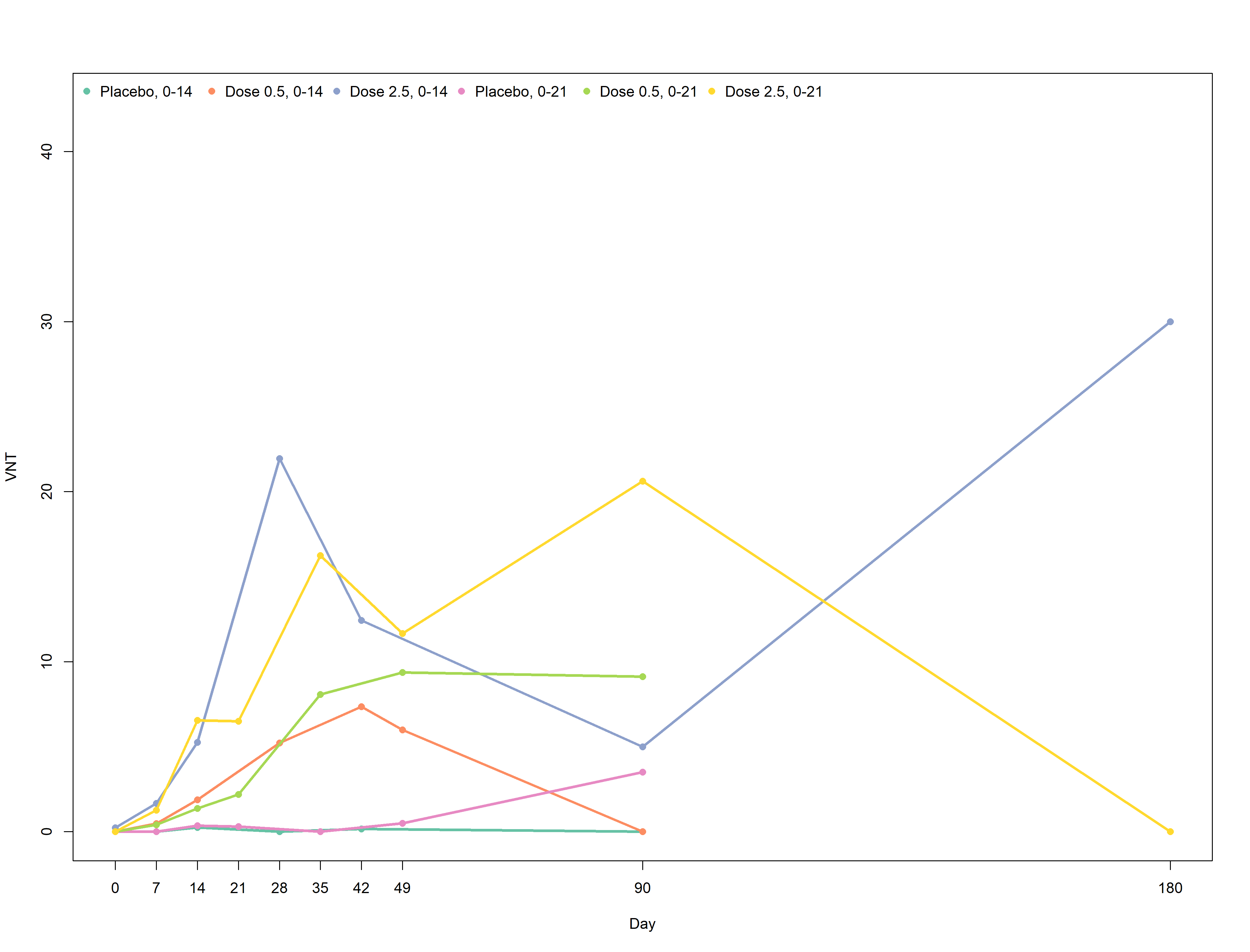


Table 20. Proportion of participants with four-fold increase in neutralizing antibody titer at predefined time intervals in two administration schedules and different vaccine strengths of 0.5 × 10^6^ TCID_50_ (5 µg/dose), 2.5 × 10^6^ TCID_50_ (10 µg/dose) and placebo (has been included in the main manuscript)

| Group | Day 0 | 2^nd^ injection | 2 wks after 2^nd^ injection | 4 wks after 2^nd^ injection |
| --- | --- | --- | --- | --- |
| Placebo, 0-14 %(n) | Ref | 0 % (12) | 0 % (11) | 0 % (6) |
| 5 µg/dose, 0-14 %(n) | Ref | 17 % (29) | 42 % (29) | 45 % (25) |
| 10 µg/dose, 0-14 %(n) | Ref | 29 % (30) | 71 % (26) | 62 % (14) |
| Placebo, 0-21 %(n) | Ref | 0 % (10) | 0 % (4) | 0 % (7) |
| 5 µg/dose, 0-21 %(n) | Ref | 21 % (24) | 75 % (14) | 60 % (12) |
| 10 µg/dose, 0-21 %(n) | Ref | 24 % (23) | 67 % (3) | 50 % (16) |

## Serum ELISA IgG levels for SARS-CoV-2

### Serum IgG levels for S antigen

Table 21. Geometric mean, geometric mean ratio (compared to placebo), geometric mean fold increase (compared to day zero), and geometric mean fold ratio for serum specific IgG antibody levels against S antigen and their 95% confidence intervals at predefined time intervals in two administration schedules and different vaccine strengths of 0.5 × 10^6^ TCID_50_ (5 µg/dose), 2.5 × 10^6^ TCID_50_ (10 µg/dose) and placebo

| **day** | **0** | **7** | **2^nd^ Injection day** | **7 days after 2^nd^ injection** | **14 days after 2^nd^ injection** | **90** | **180** |  |
| --- | --- | --- | --- | --- | --- | --- | --- | --- |
| **GM** |  | | | | | | | |
| 0-14, Placebo | 0.42 (0.26 - 0.68, N:10) | 0.38 (0.18 - 0.83, N:13) | 0.65 (0.58 - 0.75, N:12) | 0.64 (0.57 - 0.71, N:11) | 0.44 (0.3 - 0.65, N:6) | 0.85 (0.31 - 2.28, N:8) | 0.53 (0.06 - 4.66, N:3) |  |
| 0-14, Strength: 0.5 | 0.67 (0.6 - 0.75, N:24) | 0.56 (0.42 - 0.75, N:28) | 0.66 (0.59 - 0.73, N:29) | 0.57 (0.41 - 0.8, N:29) | 0.64 (0.53 - 0.78, N:25) | 1.21 (0.63 - 2.3, N:16) | 1.8 (0.29 - 11.16, N:5) |  |
| 0-14, Strength: 2.5 | 0.61 (0.43 - 0.87, N:29) | 0.56 (0.46 - 0.68, N:31) | 0.54 (0.38 - 0.77, N:30) | 0.89 (0.73 - 1.09, N:26) | 0.73 (0.52 - 1.02, N:15) | 1.75 (1.08 - 2.85, N:16) | 2.21 (1.07 - 4.55, N:9) |  |
| 0-21, Placebo | 0.62 (0.5 - 0.77, N:8) | 0.54 (0.3 - 0.95, N:9) | 0.59 (0.29 - 1.18, N:10) | 0.69 (0.47 - 1.01, N:5) | 0.44 (0.3 - 0.65, N:6) | 1.05 (0.34 - 3.28, N:6) | 0.78 (0.05 - 12.76, N:3) |  |
| 0-21, Strength: 0.5 | 0.58 (0.51 - 0.67, N:19) | 0.46 (0.33 - 0.64, N:22) | 0.6 (0.46 - 0.79, N:24) | 0.59 (0.46 - 0.76, N:14) | 0.64 (0.53 - 0.78, N:25) | 1.09 (0.53 - 2.22, N:12) | 0.45 (0.34 - 0.62, N:3) |  |
| 0-21, Strength: 2.5 | 0.39 (0.24 - 0.62, N:21) | 0.57 (0.45 - 0.72, N:22) | 0.5 (0.34 - 0.74, N:23) | 0.78 (0.6 - 1.01, N:7) | 0.73 (0.52 - 1.02, N:15) | 1.23 (0.87 - 1.75, N:20) | 1.08 (0.19 - 6.27, N:6) |  |
| **GMR** |  | | | | | | | |
| 0-14, Placebo | 1 | 1 | 1 | 1 | 1 | 1 | 1 |  |
| 0-14, Strength: 0.5 | 1.6 (0.93-2.74, N:10) | 1.46 (0.86-2.47, N:13) | 1 (0.65-1.55, N:12) | 0.9 (0.57-1.44, N:11) | 1.45 (0.9-2.32, N:6) | 1.43 (0.55-3.72, N:8) | 3.4 (0.6-19.38, N:3) |  |
| 0-14, Strength: 2.5 | 1.46 (0.86-2.47, N:24) | 1.46 (0.87-2.45, N:28) | 0.83 (0.54-1.28, N:29) | 1.4 (0.87-2.24, N:29) | 1.65 (1-2.73, N:25) | 2.07 (0.79-5.4, N:16) | 4.17 (0.85-20.42, N:5) |  |
| 0-21, Placebo | 1 | 1 | 1 | 1 | 1 | 1 | 1 |  |
| 0-21, Strength: 0.5 | 0.93 (0.51-1.72, N:19) | 0.86 (0.51-1.44, N:22) | 1.03 (0.56-1.9, N:24) | 0.86 (0.58-1.29, N:14) | 1.06 (0.64-1.75, N:12) | 1.04 (0.4-2.68, N:12) | 0.58 (0.05-7.14, N:3) |  |
| 0-21, Strength: 2.5 | 0.62 (0.34-1.13, N:21) | 1.06 (0.63-1.79, N:22) | 0.86 (0.46-1.59, N:23) | 1.13 (0.72-1.78, N:7) | 1.75 (1.09-2.82, N:16) | 1.17 (0.49-2.83, N:20) | 1.38 (0.16-12.11, N:6) |  |
| **GMFI** |  | | | | | | | |
| 0-14, Placebo | 1 | 0.75 (0.24 - 2.38, N: 10) | 1.58 (0.85 - 2.94, N: 10) | 1.51 (0.9 - 2.54, N: 9) | 0.44 (0.3 - 0.65, N: 6) | 0.75 (0.26 - 2.18, N: 7) | 0.53 (0.06 - 4.66, N: 3) |  |
| 0-14, Strength: 0.5 | 1 | 0.81 (0.56 - 1.19, N: 22) | 0.99 (0.88 - 1.13, N: 23) | 0.94 (0.72 - 1.22, N: 24) | 0.64 (0.53 - 0.78, N: 25) | 1.11 (0.6 - 2.04, N: 15) | 1.8 (0.29 - 11.16, N: 5) |  |
| 0-14, Strength: 2.5 | 1 | 0.93 (0.61 - 1.43, N: 29) | 0.96 (0.6 - 1.52, N: 28) | 1.6 (1.27 - 2.02, N: 24) | 0.73 (0.52 - 1.02, N: 15) | 1.75 (1.13 - 2.71, N: 19) | 2.21 (1.07 - 4.55, N: 9) |  |
| 0-21, Placebo | 1 | 0.71 (0.31 - 1.63, N:5) | 0.76 (0.25 - 2.35, N:7) | 1.2 (0.39 - 3.74, N:2) | 0.42 (0.27 - 0.65, N:7) | 0.42 (0.27 - 0.65, N:7) | 0.78 (0.05 - 12.76, N:3) |  |
| 0-21, Strength: 0.5 | 1 | 0.73 (0.48 - 1.11, N:18) | 0.96 (0.69 - 1.33, N:19) | 1.02 (0.76 - 1.36, N:12) | 0.44 (0.32 - 0.61, N:12) | 0.44 (0.32 - 0.61, N:12) | 0.45 (0.34 - 0.62, N:3) |  |
| 0-21, Strength: 2.5 | 1 | 1.23 (0.76 - 2.02, N:20) | 1.24 (0.63 - 2.42, N:21) | 1.68 (0.7 - 4.04, N:6) | 0.73 (0.55 - 0.97, N:16) | 0.73 (0.55 - 0.97, N:16) | 1.08 (0.19 - 6.27, N:6) |  |
| **GMFR** |  | | | | | | | |
| 0-14, Placebo | 1 | 1 | 1 | 1 | 1 | 1 | 1 |  |
| 0-14, Strength: 0.5 | 1 | 0.95 (0.48 - 1.88, N: 28) | 0.64 (0.32 - 1.28, N: 29) | 0.56 (0.28 - 1.14, N: 29) | 0.96 (0.42 - 2.17, N: 25) | 0.86 (0.39 - 1.89, N: 16) | 2.02 (0.64 - 6.4, N: 5) |  |
| 0-14, Strength: 2.5 | 1 | 1.05 (0.53 - 2.05, N: 31) | 0.59 (0.3 - 1.17, N: 30) | 1 (0.5 - 2.02, N: 26) | 1.27 (0.55 - 2.95, N: 15) | 1.43 (0.65 - 3.12, N: 16) | 2.93 (1.01 - 8.53, N: 9) |  |
| 0-21, Placebo | 1 | 1 | 1 | 1 | 1 | 1 | 1 |  |
| 0-21, Strength: 0.5 | 1 | 0.89 (0.42 - 1.87, N: 28) | 1.09 (0.53 - 2.24, N: 29) | 0.9 (0.37 - 2.14, N: 25) | 1.14 (0.5 - 2.58, N: 15) | 1.1 (0.48 - 2.56, N: 16) | 0.7 (0.21 - 2.32, N: 5) |  |
| 0-21, Strength: 2.5 | 1 | 1.66 (0.79 - 3.49, N: 31) | 1.39 (0.68 - 2.86, N: 26) | 1.68 (0.66 - 4.29, N: 15) | 3.03 (1.37 - 6.69, N: 19) | 1.94 (0.87 - 4.33, N: 16) | 2.32 (0.8 - 6.74, N: 9) |  |

Figure 8. Changes in serum specific IgG antibody levels against S antigen over the study period for each individual participant and the group mean in two administration schedules and different vaccine strengths of 0.5 × 10^6^ TCID_50_ (5 µg/dose), 2.5 × 10^6^ TCID_50_ (10 µg/dose) and placebo


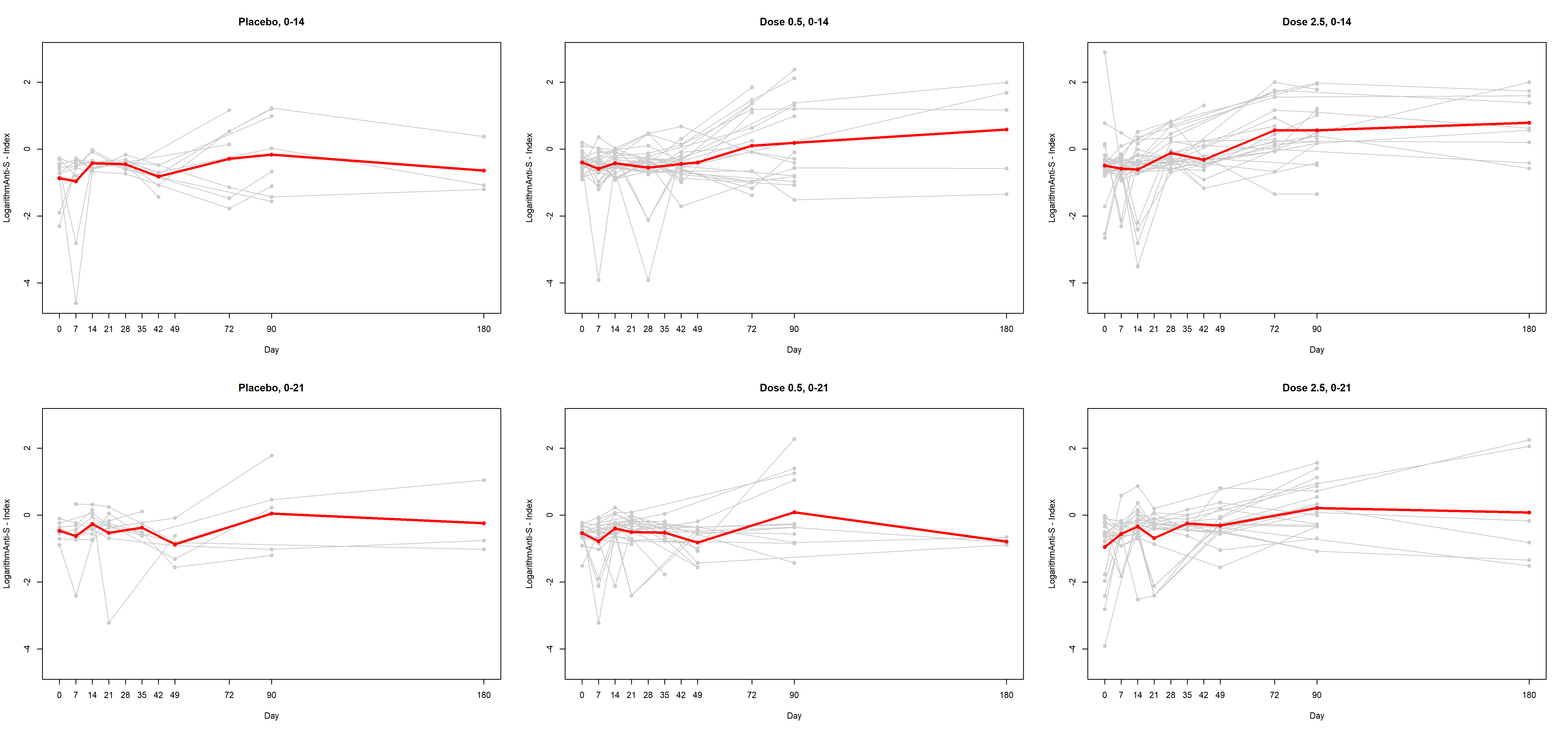


Figure 9. Changes in group means of serum specific IgG antibody levels against S antigen over the study period in two administration schedules and different vaccine strengths of 0.5 × 10^6^ TCID_50_ (5 µg/dose), 2.5 × 10^6^ TCID_50_ (10 µg/dose) and placebo


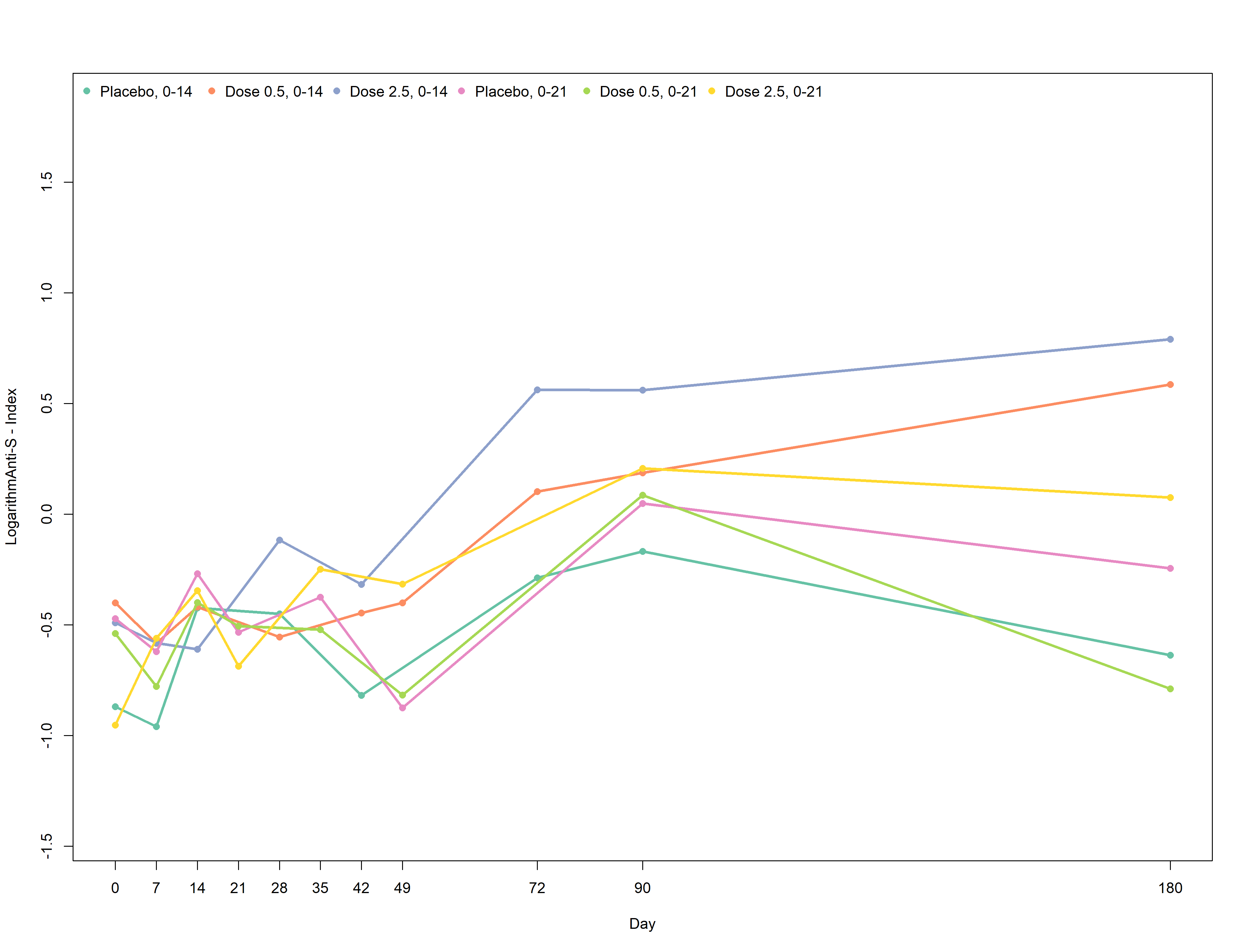


Figure 10. Serum specific IgG antibody levels against S antigen over the study period for each individual participant and the group mean and its 95% confidence interval in two administration schedules and different vaccine strengths of 0.5 × 10^6^ TCID_50_ (5 µg/dose), 2.5 × 10^6^ TCID_50_ (10 µg/dose) and placebo


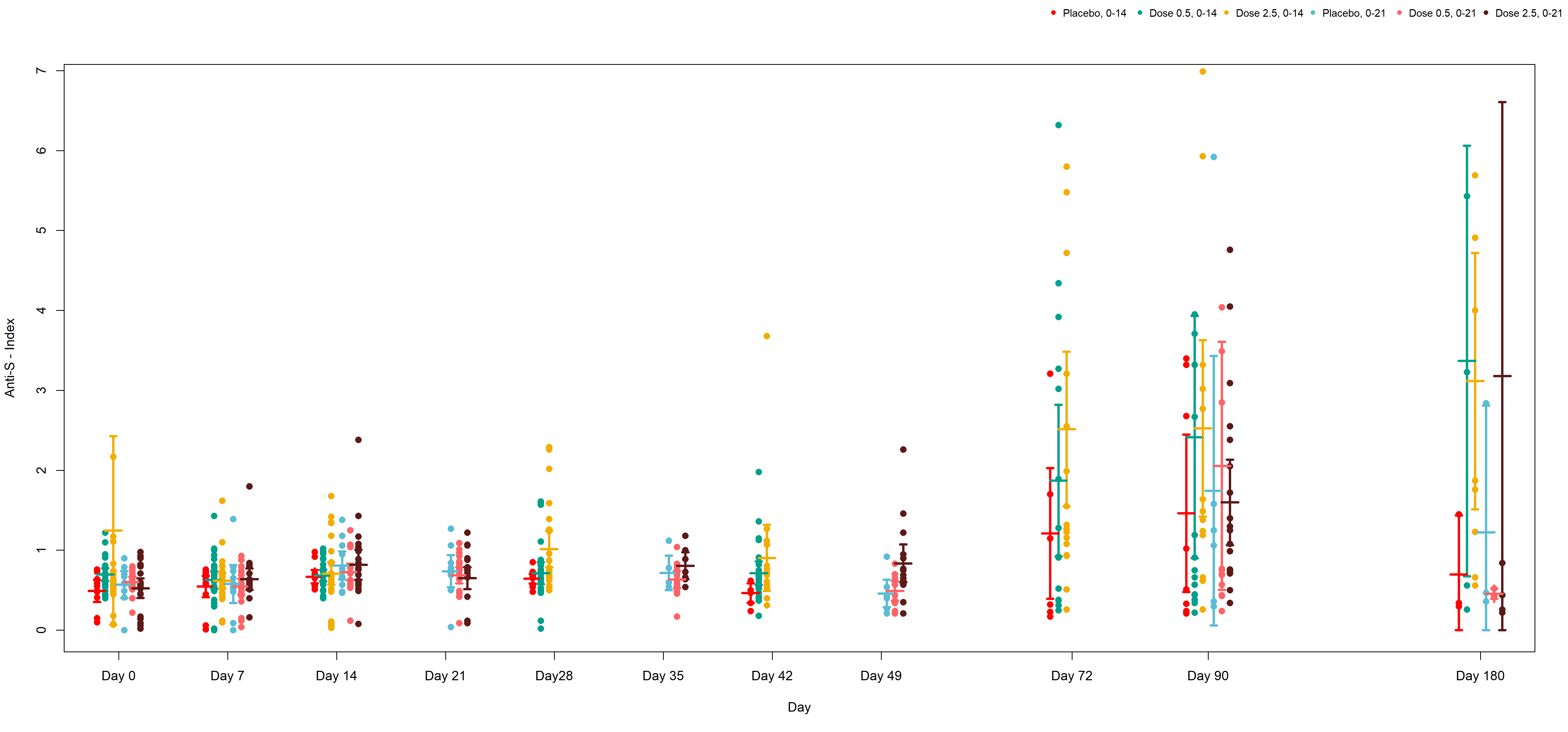


Figure 11. Serum specific IgG antibody levels against S antigen in natural logarithmic scale over the study period for each individual participant and the group mean and its 95% confidence interval in two administration schedules and different vaccine strengths of 0.5 × 10^6^ TCID_50_ (5 µg/dose), 2.5 × 10^6^ TCID_50_ (10 µg/dose) and placebo


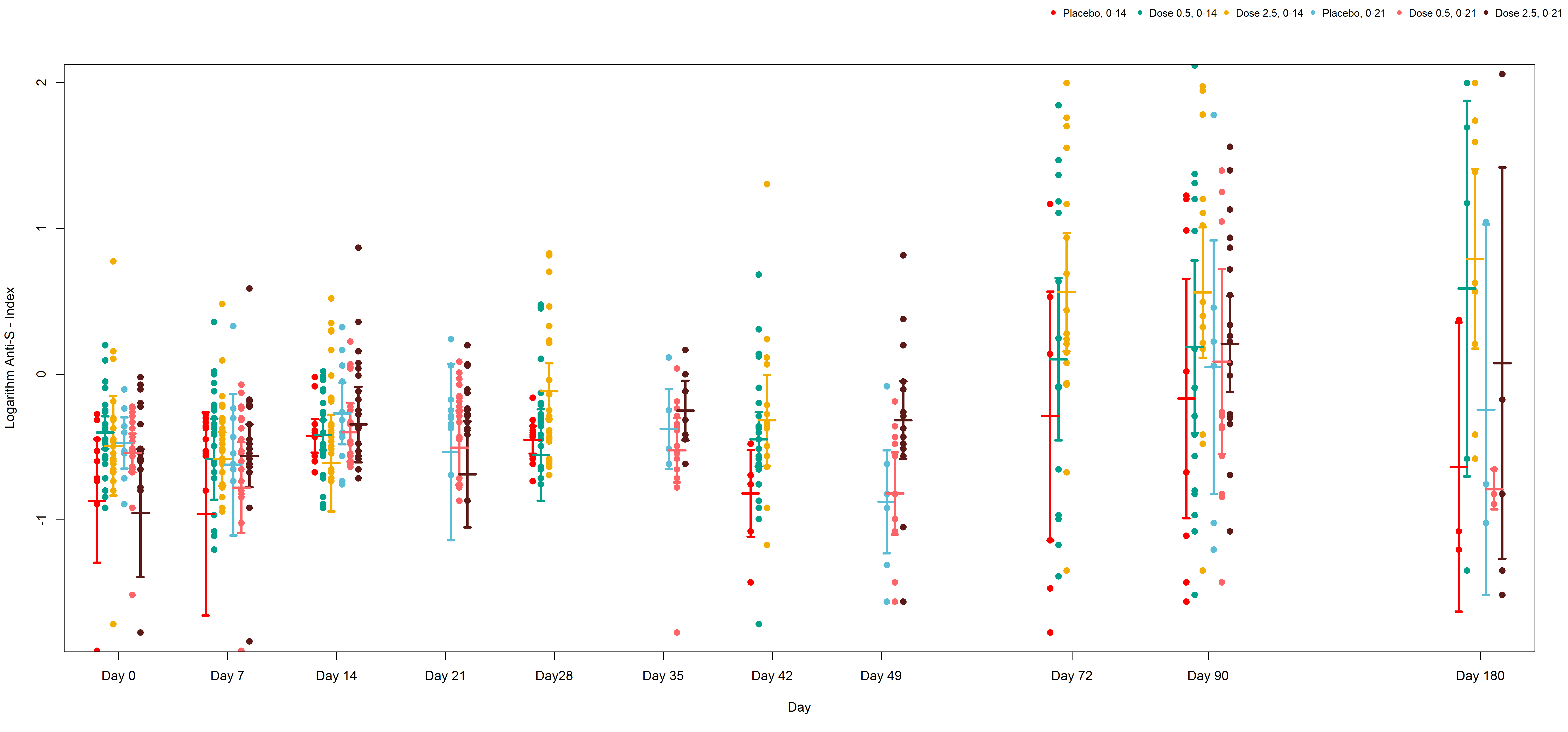


### Serum IgG levels for [Nucleocapsid](https://www.google.com/search?rlz=1C1CHZN_enFR966GB966&sxsrf=ALiCzsarwGeaCedO0cwcK8fOKIG7j-OVFw:1651988777631&q=Nucleocapsid&spell=1&sa=X&ved=2ahUKEwjay43imc_3AhWn4IUKHcy1D1UQkeECKAB6BAgCEDM) antigen

Table 22. Geometric mean, geometric mean ratio (compared to placebo), geometric mean fold increase (compared to day zero), and geometric mean fold ratio for serum specific IgG antibody levels against N antigen and their 95% confidence intervals at predefined time intervals in two administration schedules and different vaccine strengths of 0.5 × 10^6^ TCID_50_ (5 µg/dose), 2.5 × 10^6^ TCID_50_ (10 µg/dose) and placebo

| **day** | **0** | **7** | **2^nd^ Injection day** | **7 days after 2^nd^ injection** | **7 days after 2^nd^ injection** | **90** | **180** |  | |
| --- | --- | --- | --- | --- | --- | --- | --- | --- | --- |
| **GM** |  | | | | | | | |  |
| 0-14, Placebo | 0.39 (0.24 - 0.63, N:10) | 0.41 (0.25 - 0.68, N:13) | 0.85 (0.59 - 1.22, N:12) | 0.53 (0.34 - 0.83, N:11) | 0.51 (0.2 - 1.31, N:6) | 0.68 (0.51 - 0.9, N:8) | 0.56 (0.33 - 0.95, N:3) |  | |
| 0-14, Strength: 0.5 | 0.3 (0.23 - 0.38, N:24) | 0.34 (0.25 - 0.46, N:29) | 0.69 (0.45 - 1.05, N:29) | 1.6 (1.08 - 2.39, N:29) | 1.75 (1.2 - 2.53, N:25) | 1.5 (0.89 - 2.52, N:16) | 2.25 (0.41 - 12.3, N:5) |  | |
| 0-14, Strength: 2.5 | 0.27 (0.19 - 0.38, N:27) | 0.5 (0.33 - 0.76, N:31) | 1.28 (0.81 - 2.02, N:30) | 4.48 (3.44 - 5.83, N:26) | 3.61 (2.6 - 5.02, N:15) | 2.9 (1.7 - 4.96, N:16) | 2.61 (1.31 - 5.19, N:9) |  | |
| 0-21, Placebo | 0.25 (0.1 - 0.62, N:9) | 0.32 (0.14 - 0.7, N:10) | 0.63 (0.35 - 1.13, N:10) | 0.43 (0.2 - 0.93, N:5) | 0.71 (0.4 - 1.27, N:7) | 0.8 (0.28 - 2.32, N:6) | 1.42 (0.08 - 25.21, N:3) |  | |
| 0-21, Strength: 0.5 | 0.24 (0.17 - 0.35, N:19) | 0.34 (0.2 - 0.57, N:22) | 1.52 (1.1 - 2.1, N:24) | 2.62 (1.79 - 3.83, N:14) | 2.26 (0.99 - 5.15, N:12) | 2.43 (1.46 - 4.04, N:12) | 1.89 (0.1 - 34.91, N:3) |  | |
| 0-21, Strength: 2.5 | 0.21 (0.14 - 0.3, N:21) | 0.51 (0.3 - 0.86, N:22) | 1.41 (0.91 - 2.2, N:23) | 6.25 (2.76 - 14.14, N:7) | 4.05 (2.58 - 6.36, N:16) | 2.24 (1.34 - 3.73, N:20) | 1.82 (0.36 - 9.22, N:6) |  | |
| **GMR** |  | | | | | | | |  |
| 0-14, Placebo | 1 | 1 | 1 | 1 | 1 | 1 | 1 |  | |
| 0-14, Strength: 0.5 | 0.77 (0.44-1.36, N:10) | 0.82 (0.42-1.57, N:13) | 0.81 (0.38-1.72, N:12) | 3.04 (1.66-5.57, N:11) | 3.45 (1.64-7.29, N:6) | 2.21 (1-4.89, N:8) | 4 (0.84-19.16, N:3) |  | |
| 0-14, Strength: 2.5 | 0.7 (0.4-1.21, N:24) | 1.21 (0.63-2.31, N:29) | 1.51 (0.71-3.19, N:29) | 8.49 (4.59-15.68, N:29) | 7.14 (3.23-15.79, N:25) | 4.29 (1.94-9.49, N:16) | 4.64 (1.11-19.41, N:5) |  | |
| 0-21, Placebo | 1 | 1 | 1 | 1 | 1 | 1 | 1 |  | |
| 0-21, Strength: 0.5 | 0.97 (0.48-1.96, N:19) | 1.07 (0.44-2.62, N:22) | 2.43 (1.24-4.75, N:24) | 6.12 (2.82-13.29, N:14) | 3.17 (1.21-8.31, N:12) | 3.02 (1.1-8.32, N:12) | 1.33 (0.1-17.36, N:3) |  | |
| 0-21, Strength: 2.5 | 0.82 (0.41-1.63, N:21) | 1.6 (0.66-3.92, N:22) | 2.25 (1.15-4.42, N:23) | 14.61 (6.12-34.91, N:7) | 5.68 (2.27-14.24, N:16) | 2.78 (1.08-7.14, N:20) | 1.29 (0.14-11.89, N:6) |  | |
| **GMFI** |  | | | | | | | |  |
| 0-14, Placebo | 1 | 1.26 (0.69 - 2.3, N: 10) | 2.53 (1.69 - 3.81, N: 10) | 1.51 (0.99 - 2.31, N: 9) | 0.51 (0.2 - 1.31, N: 6) | 0.68 (0.51 - 0.9, N: 8) | 0.56 (0.33 - 0.95, N: 3) |  | |
| 0-14, Strength: 0.5 | 1 | 1.13 (0.84 - 1.51, N: 23) | 2.07 (1.3 - 3.29, N: 23) | 4.96 (3.35 - 7.35, N: 24) | 1.75 (1.2 - 2.53, N: 25) | 1.5 (0.89 - 2.52, N: 16) | 2.25 (0.41 - 12.3, N: 5) |  | |
| 0-14, Strength: 2.5 | 1 | 2.09 (1.46 - 2.98, N: 27) | 6.05 (4.52 - 8.11, N: 26) | 14.73 (9.3 - 23.31, N: 22) | 3.61 (2.6 - 5.02, N: 15) | 2.9 (1.7 - 4.96, N: 16) | 2.61 (1.31 - 5.19, N: 9) |  | |
| 0-21, Placebo | 1 | 1.36 (0.65 - 2.86, N:7) | 2.3 (0.92 - 5.73, N:8) | 2.84 (0.28 - 28.39, N:3) | 0.71 (0.4 - 1.27, N:7) | 0.8 (0.28 - 2.32, N:6) | 1.42 (0.08 - 25.21, N:3) |  | |
| 0-21, Strength: 0.5 | 1 | 1.3 (0.82 - 2.07, N:18) | 6.5 (4.77 - 8.86, N:19) | 9.17 (4.87 - 17.24, N:12) | 2.26 (0.99 - 5.15, N:12) | 2.43 (1.46 - 4.04, N:12) | 1.89 (0.1 - 34.91, N:3) |  | |
| 0-21, Strength: 2.5 | 1 | 2.26 (1.28 - 3.97, N:20) | 6.65 (3.75 - 11.79, N:21) | 38.09 (10.87 - 133.5, N:6) | 4.05 (2.58 - 6.36, N:16) | 2.24 (1.34 - 3.73, N:20) | 1.82 (0.36 - 9.22, N:6) |  | |
| **GMFR** |  | | | | | | | |  |
| 0-14, Placebo | 1 | 1 | 1 | 1 | 1 | 1 | 1 |  | |
| 0-14, Strength: 0.5 | 1 | 0.9 (0.49 - 1.64, N: 29) | 0.92 (0.5 - 1.69, N: 29) | 3.2 (1.73 - 5.93, N: 29) | 3.22 (1.56 - 6.63, N: 25) | 1.96 (0.98 - 3.95, N: 16) | 2.23 (0.79 - 6.24, N: 5) |  | |
| 0-14, Strength: 2.5 | 1 | 1.73 (0.96 - 3.14, N: 31) | 2.16 (1.18 - 3.92, N: 30) | 12.13 (6.54 - 22.49, N: 26) | 9.69 (4.59 - 20.45, N: 15) | 5.02 (2.51 - 10.04, N: 16) | 3.9 (1.5 - 10.14, N: 9) |  | |
| 0-21, Placebo | 1 | 1 | 1 | 1 | 1 | 1 | 1 |  | |
| 0-21, Strength: 0.5 | 1 | 1.04 (0.49 - 2.19, N: 29) | 2.49 (1.2 - 5.19, N: 29) | 4.13 (1.68 - 10.14, N: 25) | 3.76 (1.63 - 8.68, N: 15) | 3.01 (1.28 - 7.09, N: 16) | 1.85 (0.53 - 6.46, N: 5) |  | |
| 0-21, Strength: 2.5 | 1 | 1.73 (0.83 - 3.62, N: 31) | 2.76 (1.33 - 5.71, N: 26) | 14.95 (5.67 - 39.35, N: 15) | 7.86 (3.53 - 17.52, N: 19) | 3.72 (1.65 - 8.39, N: 16) | 2.18 (0.72 - 6.61, N: 9) |  | |

Figure 12. Changes in serum specific IgG antibody levels against N antigen over the study period for each individual participant and the group mean in two administration schedules and different vaccine strengths of 0.5 × 10^6^ TCID_50_ (5 µg/dose), 2.5 × 10^6^ TCID_50_ (10 µg/dose) and placebo


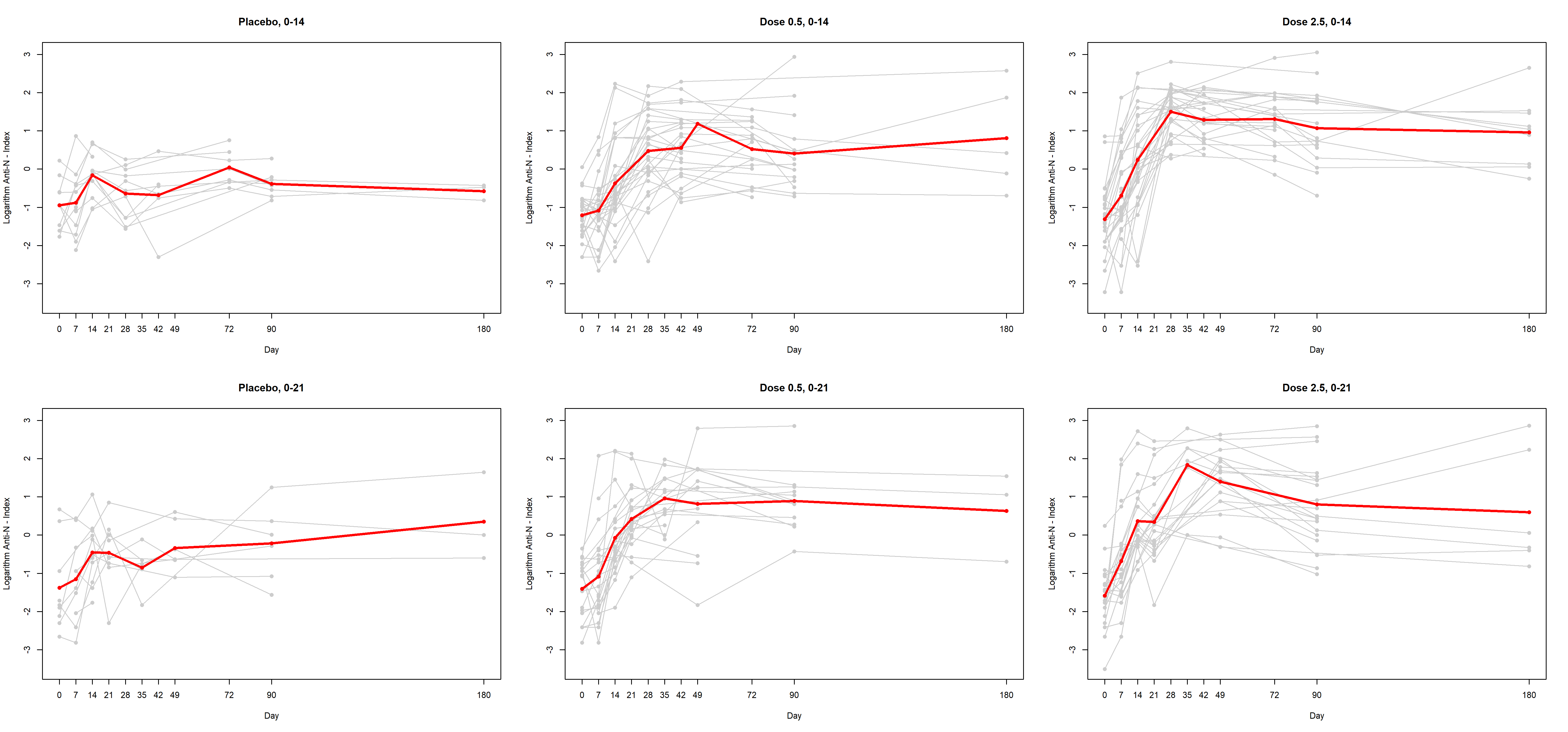


Figure 13. Changes in group means of serum specific IgG antibody levels against N antigen over the study period in two administration schedules and different vaccine strengths of 0.5 × 10^6^ TCID_50_ (5 µg/dose), 2.5 × 10^6^ TCID_50_ (10 µg/dose) and placebo


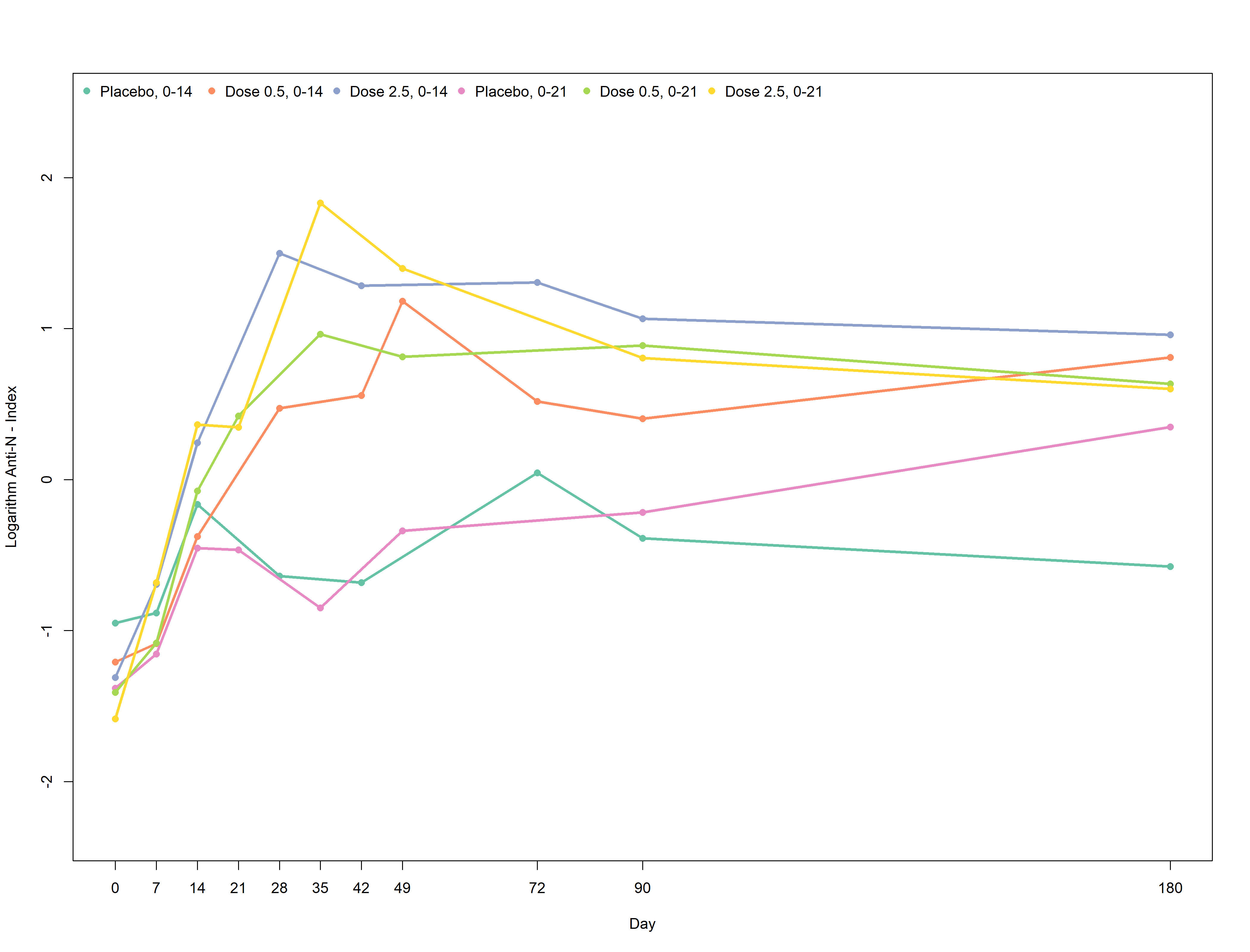


Figure 14. Serum specific IgG antibody levels against N antigen over the study period for each individual participant and the group mean and its 95% confidence interval in two administration schedules and different vaccine strengths of 0.5 × 10^6^ TCID_50_ (5 µg/dose), 2.5 × 10^6^ TCID_50_ (10 µg/dose) and placebo


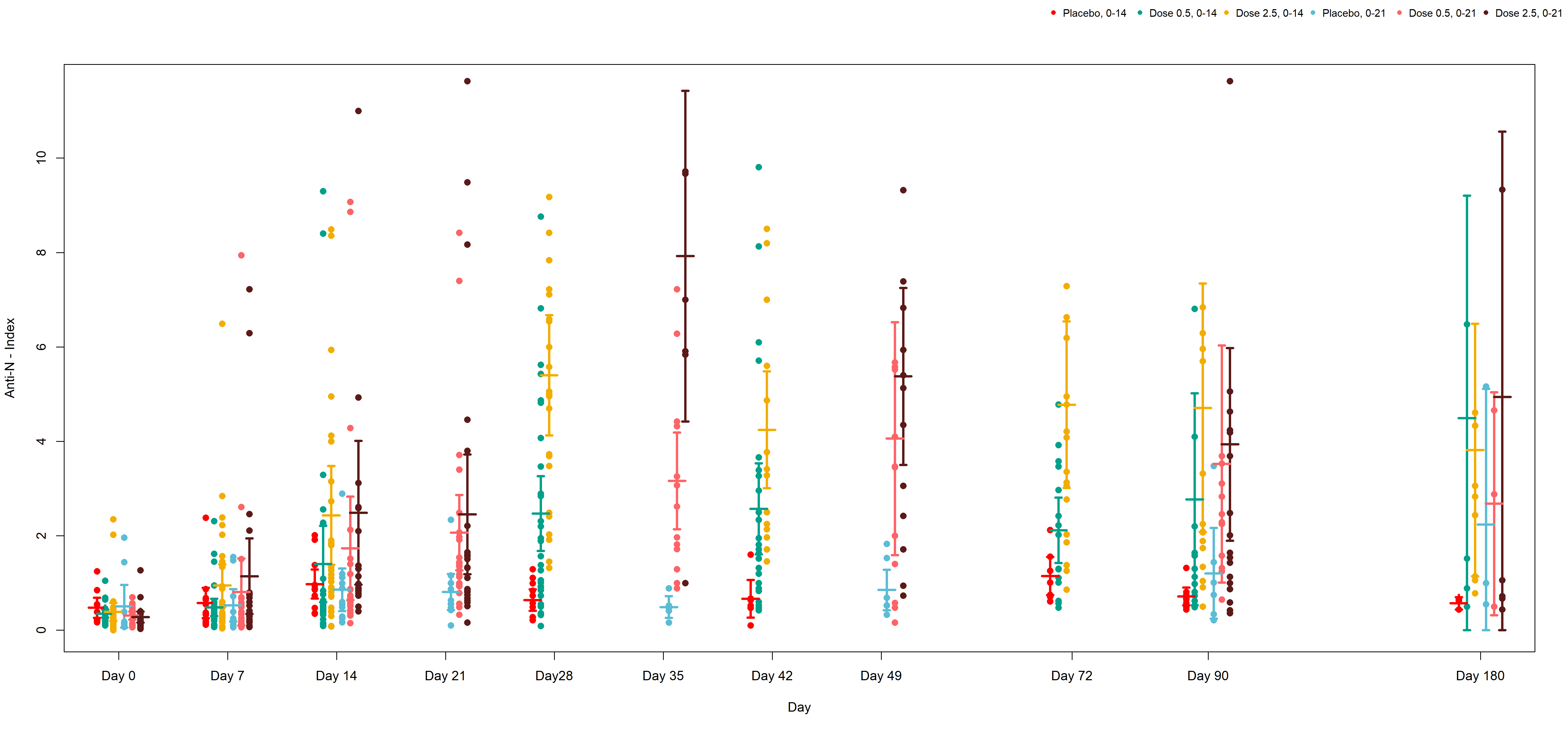


Figure 15. Serum specific IgG antibody levels against N antigen in natural logarithmic scale over the study period for each individual participant and the group mean and its 95% confidence interval in two administration schedules and different vaccine strengths of 0.5 × 10^6^ TCID_50_ (5 µg/dose), 2.5 × 10^6^ TCID_50_ (10 µg/dose) and placebo


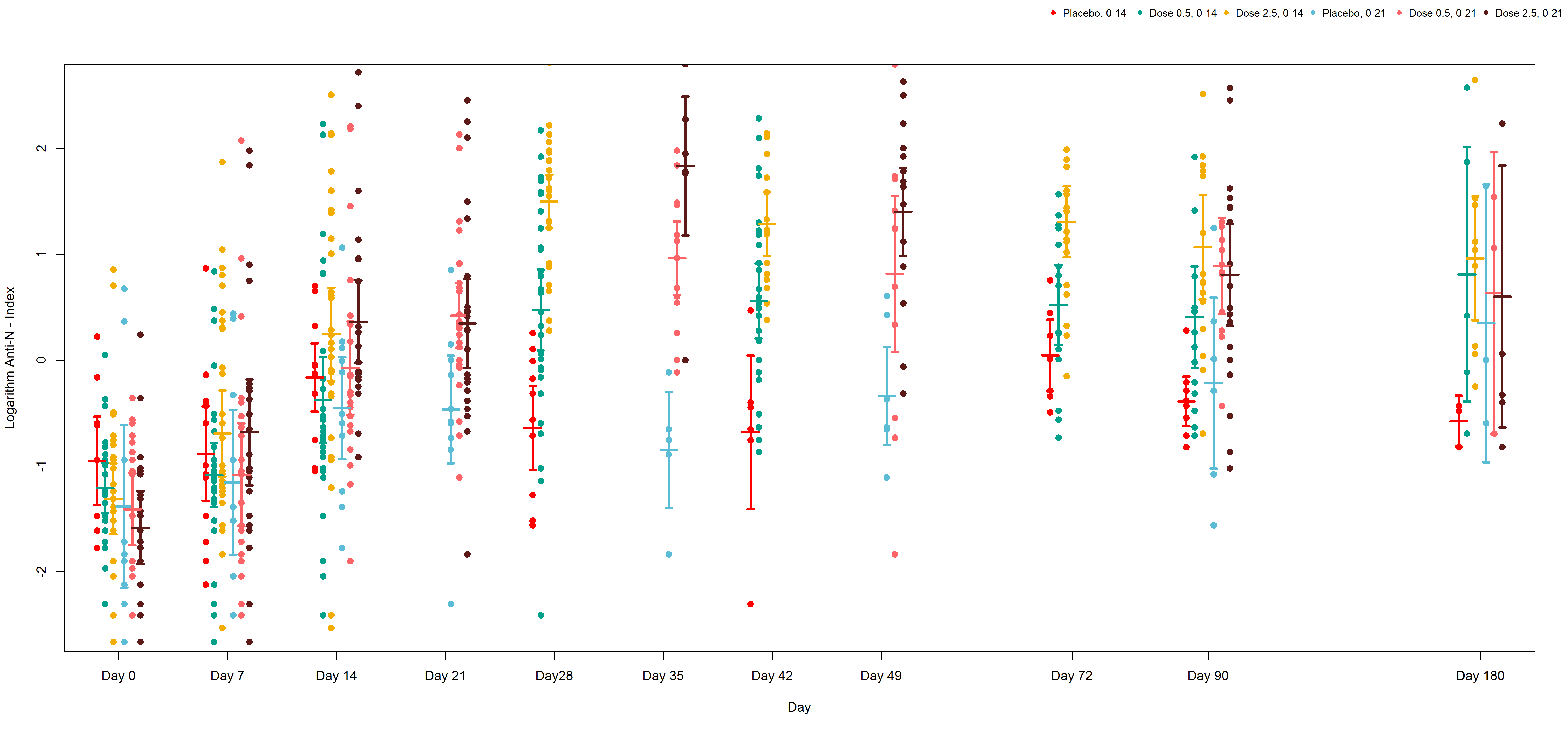


## Serum ELISA lymphokine levels

Figure 16. Serum concentrations pg/ml of IL-6 in study participants at day 0 and 14 days after 2^nd^ injection in two administration schedules and different vaccine strengths of 0.5 × 10^6^ TCID_50_ (5 µg/dose), 2.5 × 10^6^ TCID_50_ (10 µg/dose) and placebo


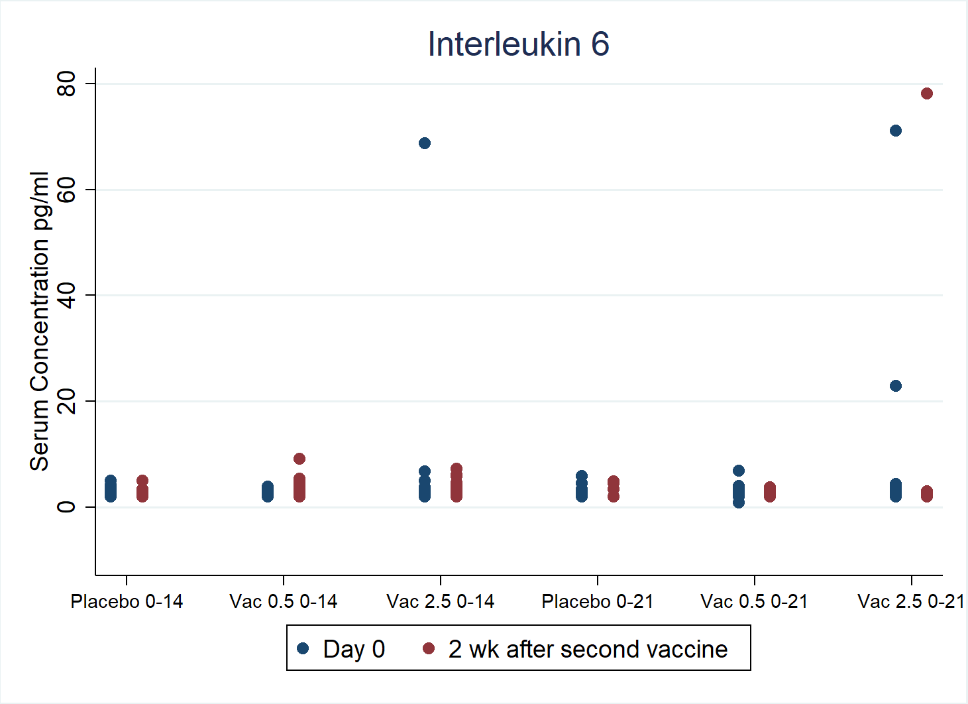


Figure 17. Serum concentrations pg/ml of IL-2 in study participants at day 0 and 14 days after 2^nd^ injection in two administration schedules and different vaccine strengths of 0.5 × 10^6^ TCID_50_ (5 µg/dose), 2.5 × 10^6^ TCID_50_ (10 µg/dose) and placebo


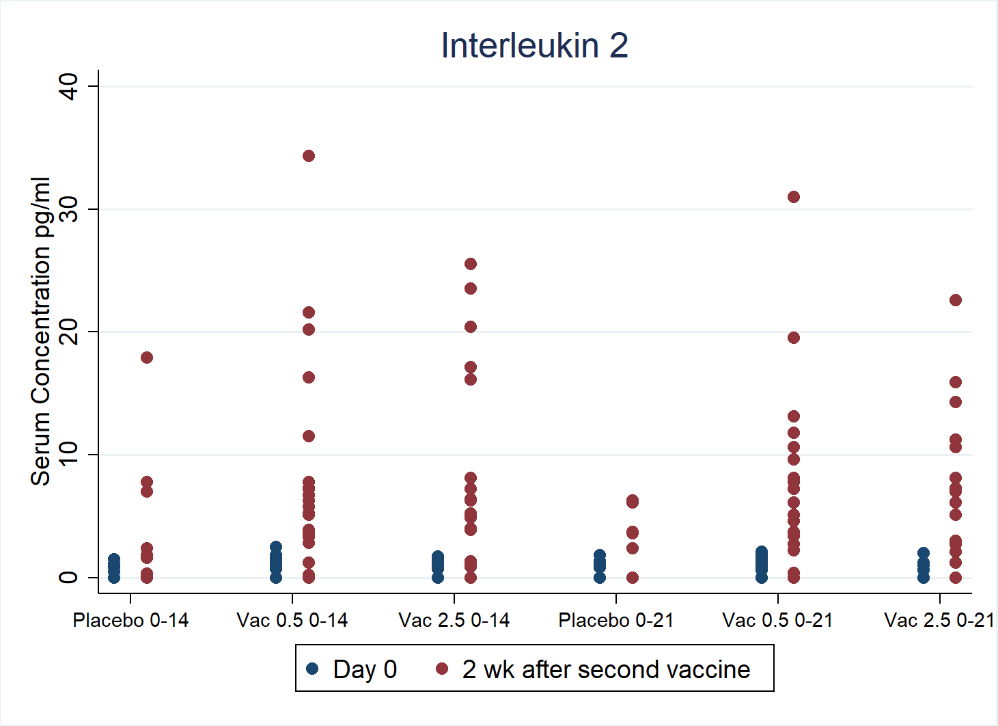


Figure 18. Serum concentrations pg/ml of IL-4 in study participants at day 0 and 14 days after 2^nd^ injection in two administration schedules and different vaccine strengths of 0.5 × 10^6^ TCID_50_ (5 µg/dose), 2.5 × 10^6^ TCID_50_ (10 µg/dose) and placebo


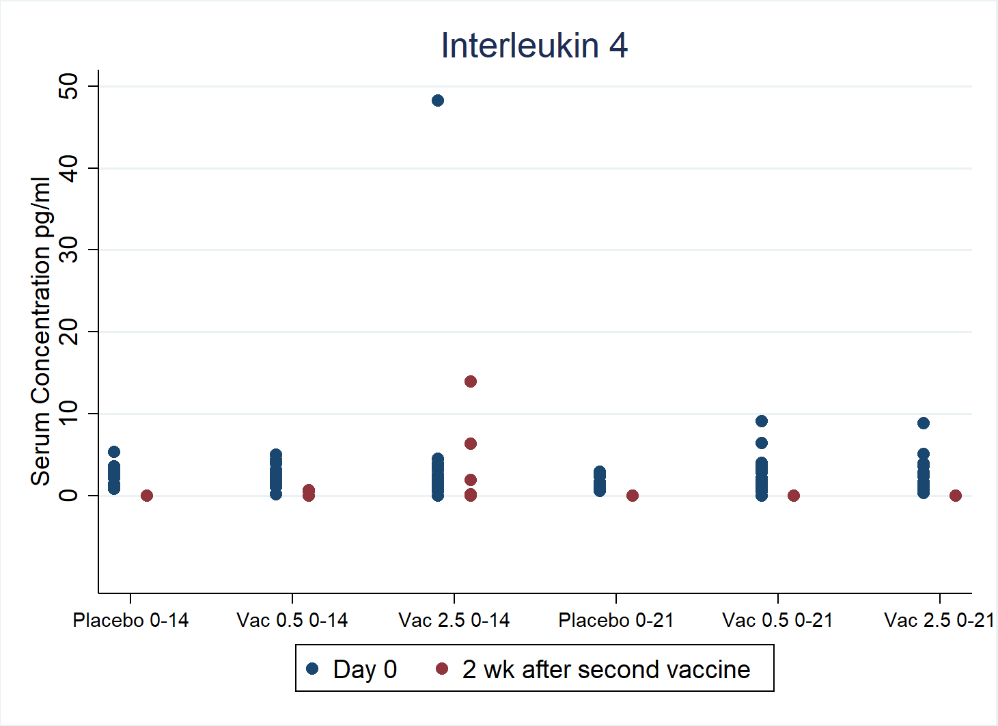


Figure 19. Serum concentrations pg/ml of IL-5 in study participants at day 0 and 14 days after 2^nd^ injection in two administration schedules and different vaccine strengths of 0.5 × 10^6^ TCID_50_ (5 µg/dose), 2.5 × 10^6^ TCID_50_ (10 µg/dose) and placebo


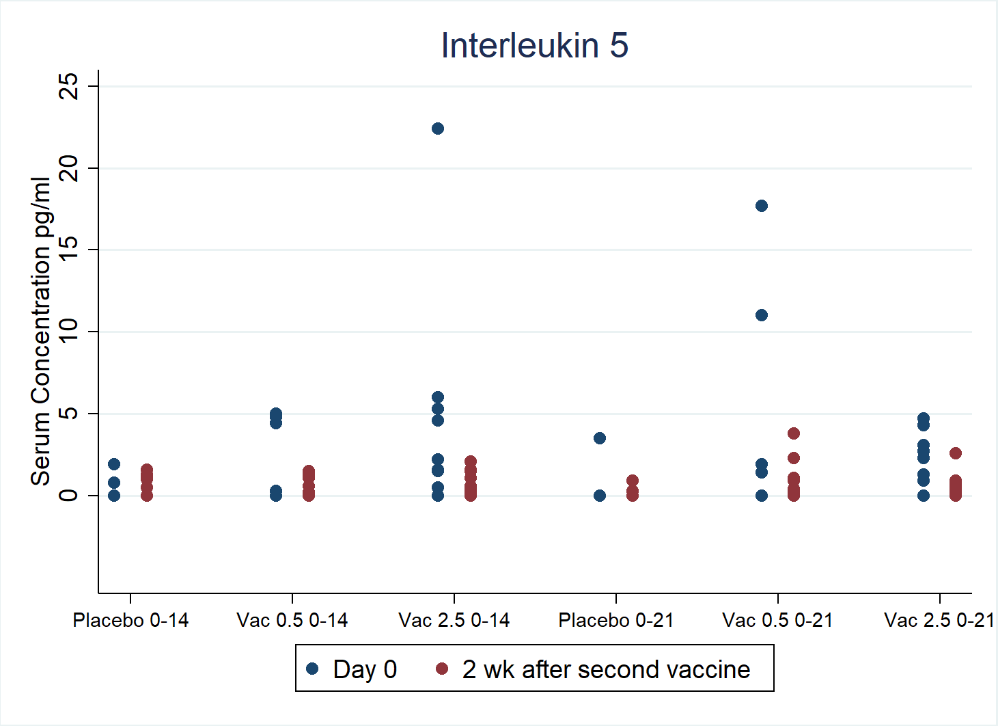


Figure 20. Serum concentrations pg/ml of TNF-α in study participants at day 0 and 14 days after 2^nd^ injection in two administration schedules and different vaccine strengths of 0.5 × 10^6^ TCID_50_ (5 µg/dose), 2.5 × 10^6^ TCID_50_ (10 µg/dose) and placebo


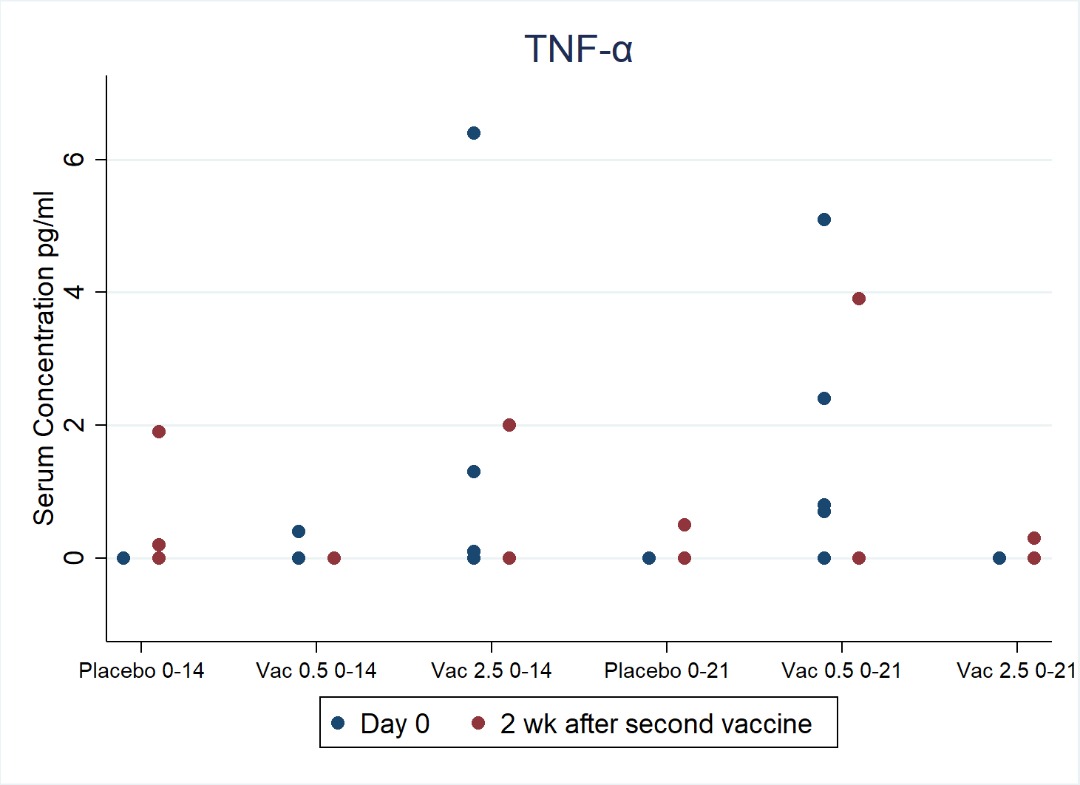


Figure 21. Serum concentrations pg/ml of IL-10 in study participants at day 0 and 14 days after 2^nd^ injection in two administration schedules and different vaccine strengths of 0.5 × 10^6^ TCID_50_ (5 µg/dose), 2.5 × 10^6^ TCID_50_ (10 µg/dose) and placebo


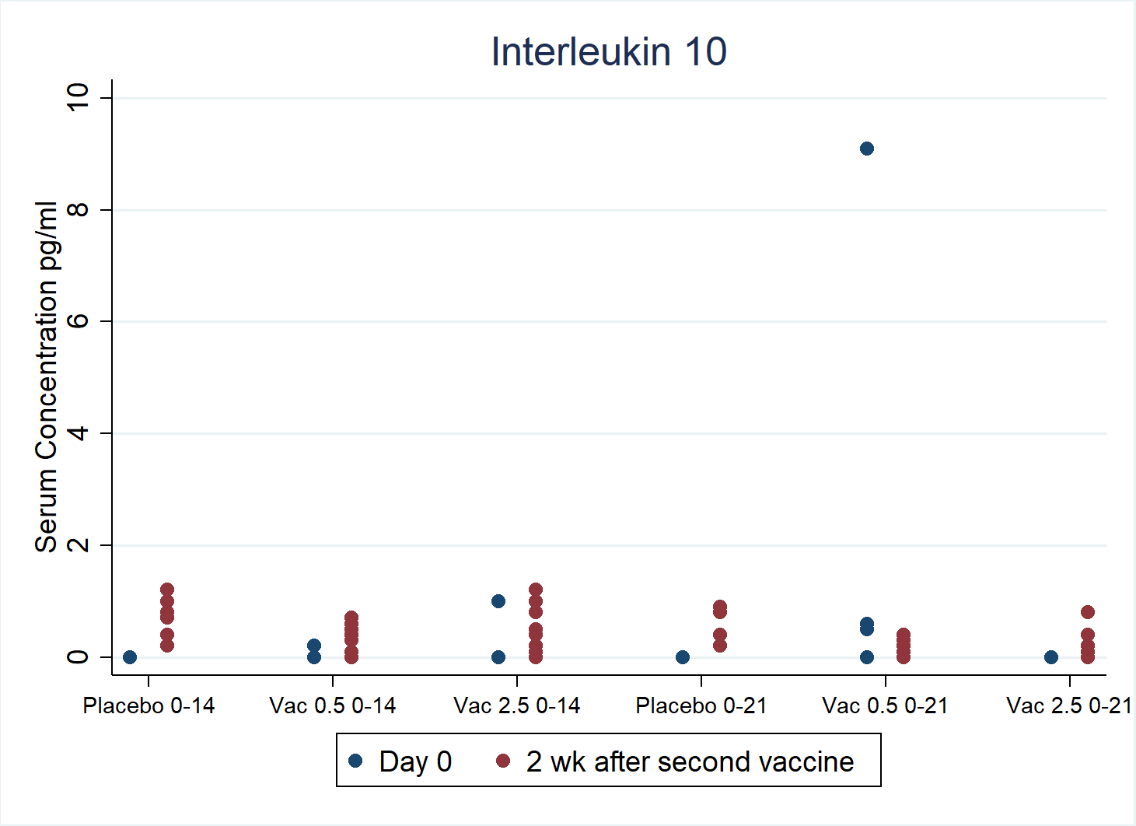


Figure 22. Serum concentrations pg/ml of IL-12 in study participants at day 0 and 14 days after 2^nd^ injection in two administration schedules and different vaccine strengths of 0.5 × 10^6^ TCID_50_ (5 µg/dose), 2.5 × 10^6^ TCID_50_ (10 µg/dose) and placebo


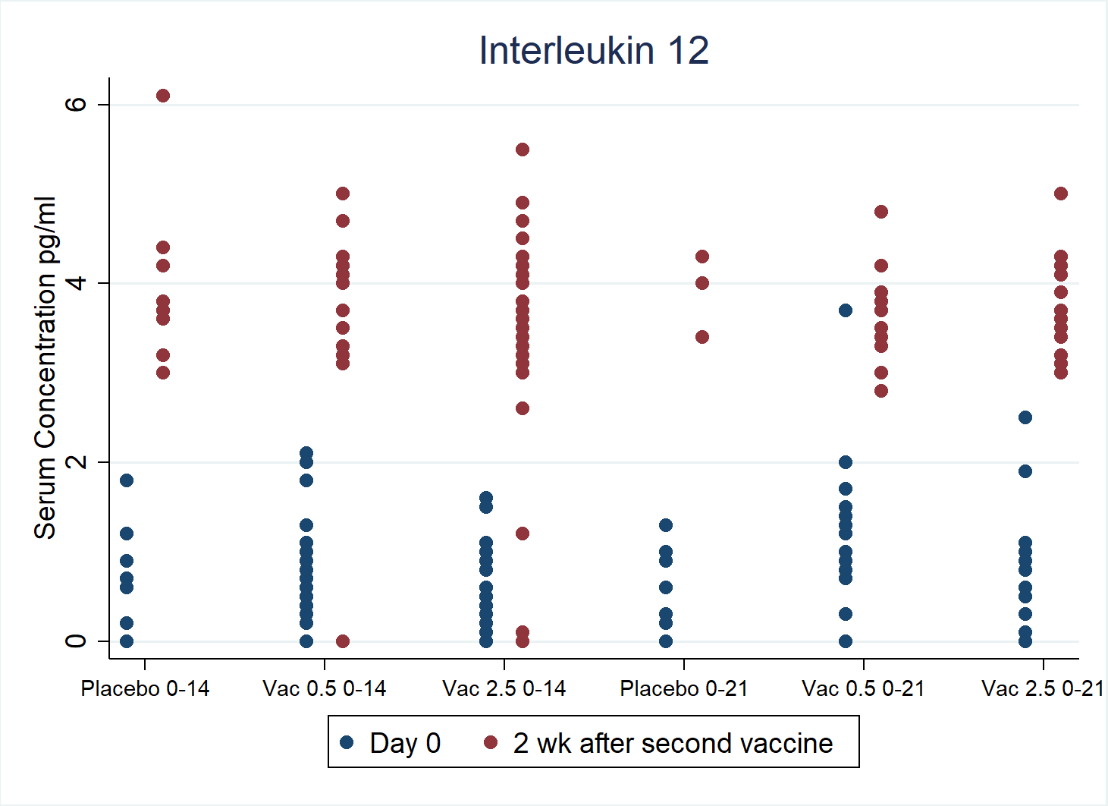


Figure 23. Serum concentrations pg/ml of IL-17 in study participants at day 0 and 14 days after 2^nd^ injection in two administration schedules and different vaccine strengths of 0.5 × 10^6^ TCID_50_ (5 µg/dose), 2.5 × 10^6^ TCID_50_ (10 µg/dose) and placebo


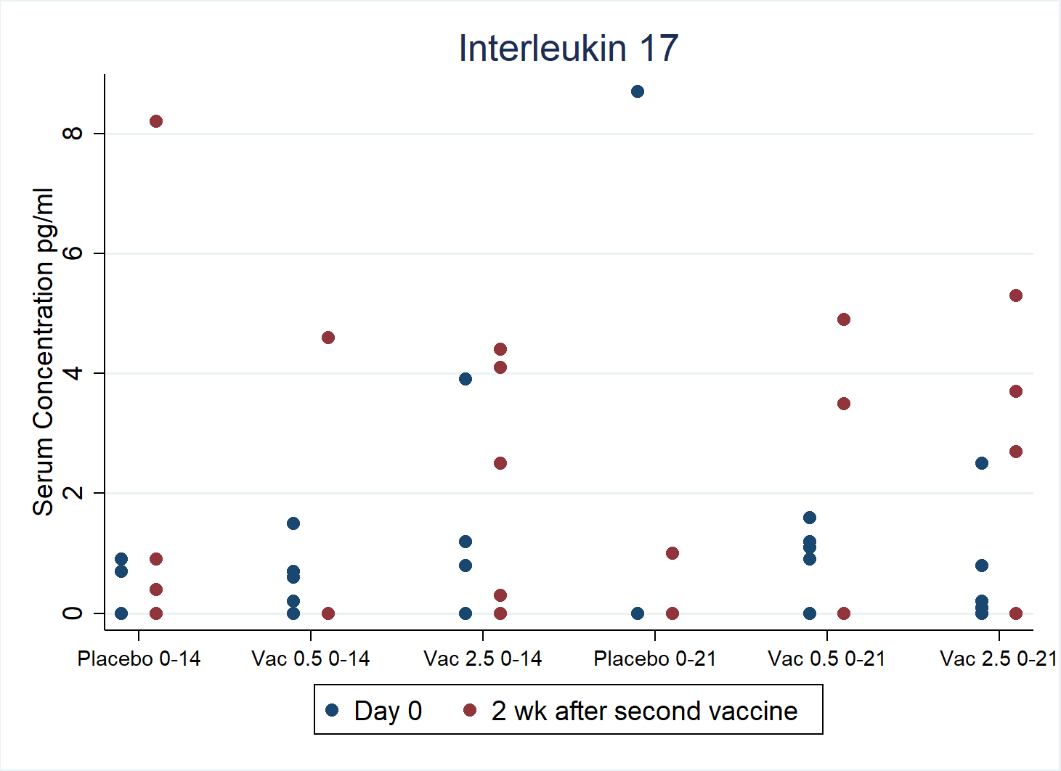


Figure 24. Serum concentrations pg/ml of γ-INF in study participants at day 0 and 14 days after 2^nd^ injection in two administration schedules and different vaccine strengths of 0.5 × 10^6^ TCID_50_ (5 µg/dose), 2.5 × 10^6^ TCID_50_ (10 µg/dose) and placebo


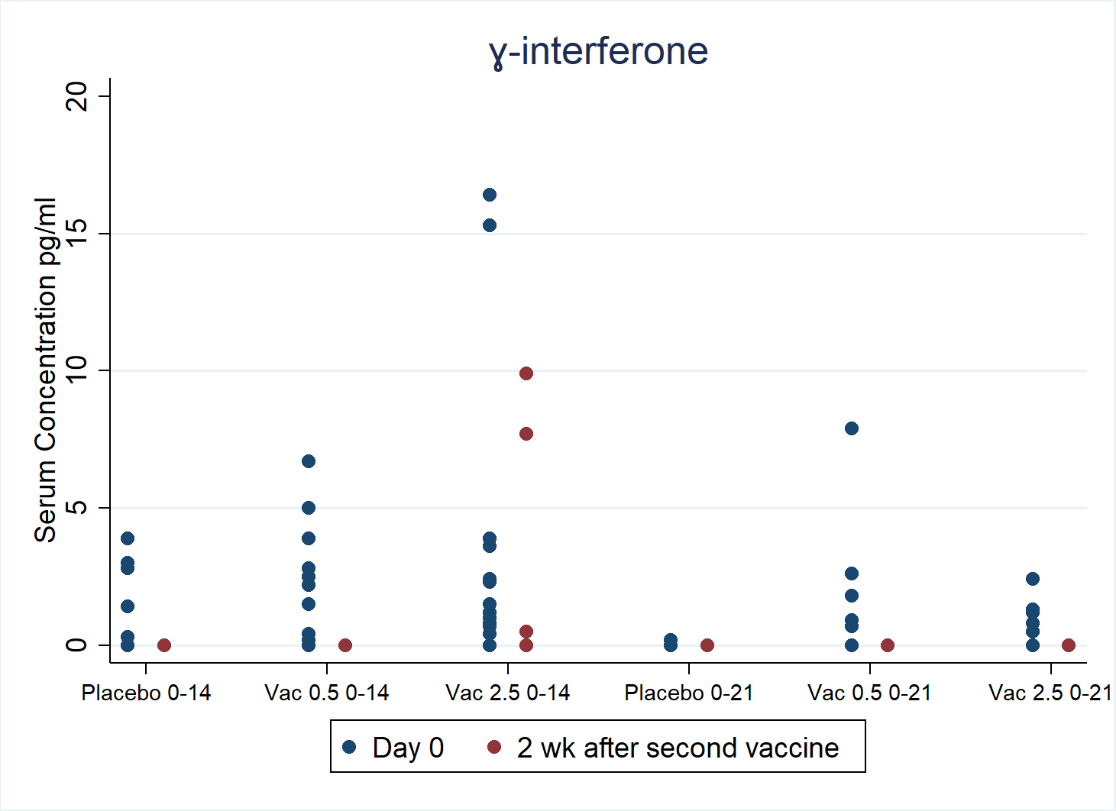


## Peripheral blood flowcytometry for lymphocyte subtypes composition

Figure 25. Proportion of lymphocytes in peripheral blood measured by flowcytometry at day 0 and two weeks after 2^nd^ injection in two administration schedules and different vaccine strengths of 0.5 × 10^6^ TCID_50_ (5 µg/dose), 2.5 × 10^6^ TCID_50_ (10 µg/dose) and placebo


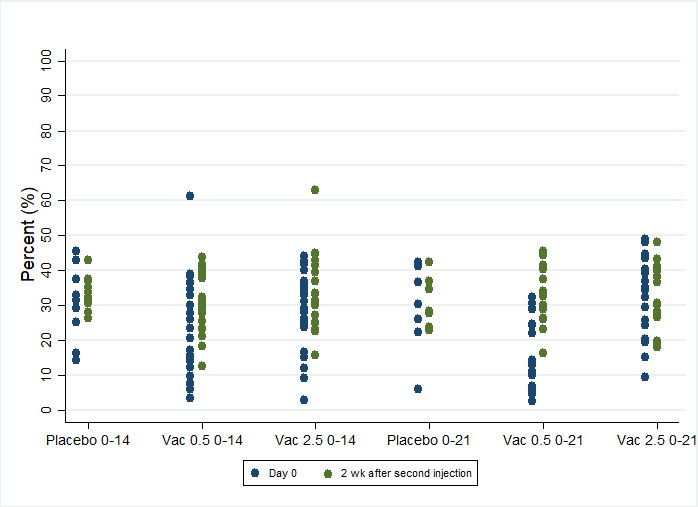


Figure 26. Proportion of lymphocytic population expressing CD3 marker in peripheral blood measured by flowcytometry at day 0 and two weeks after 2^nd^ injection in two administration schedules and different vaccine strengths of 0.5 × 10^6^ TCID_50_ (5 µg/dose), 2.5 × 10^6^ TCID_50_ (10 µg/dose) and placebo


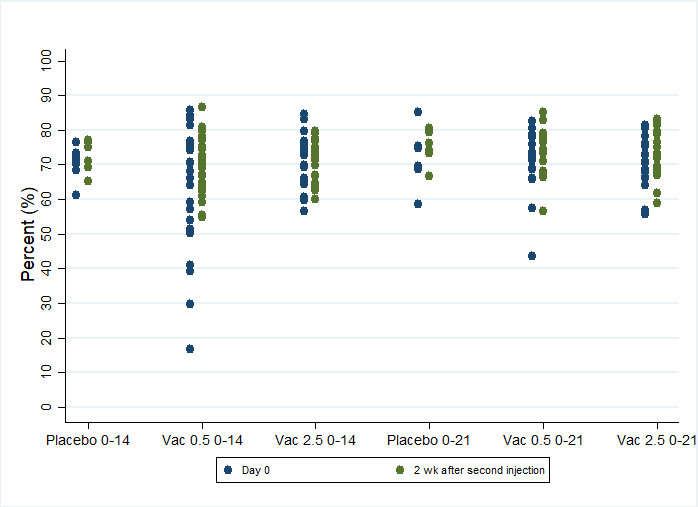


Figure 27. Proportion of lymphocytic population expressing CD4 marker within CD3 positive subset in peripheral blood measured by flowcytometry at day 0 and two weeks after 2^nd^ injection in two administration schedules and different vaccine strengths of 0.5 × 10^6^ TCID_50_ (5 µg/dose), 2.5 × 10^6^ TCID_50_ (10 µg/dose) and placebo


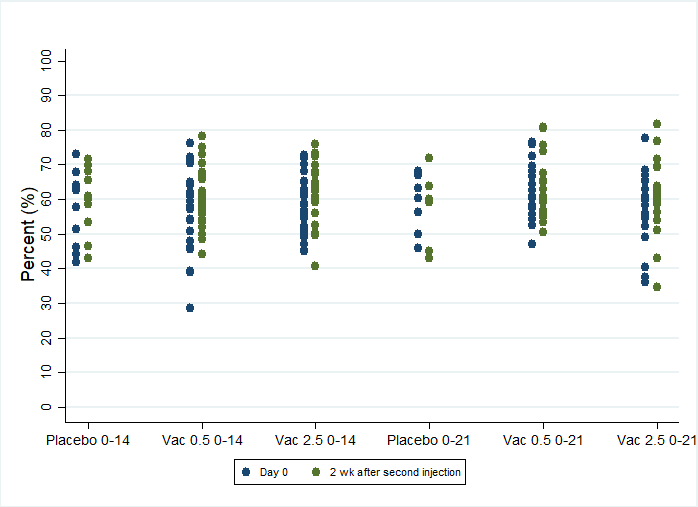


Figure 28. Proportion of lymphocytic population expressing CD8 marker within CD3 positive subset in peripheral blood measured by flowcytometry at day 0 and two weeks after 2^nd^ injection in two administration schedules and different vaccine strengths of 0.5 × 10^6^ TCID_50_ (5 µg/dose), 2.5 × 10^6^ TCID_50_ (10 µg/dose) and placebo


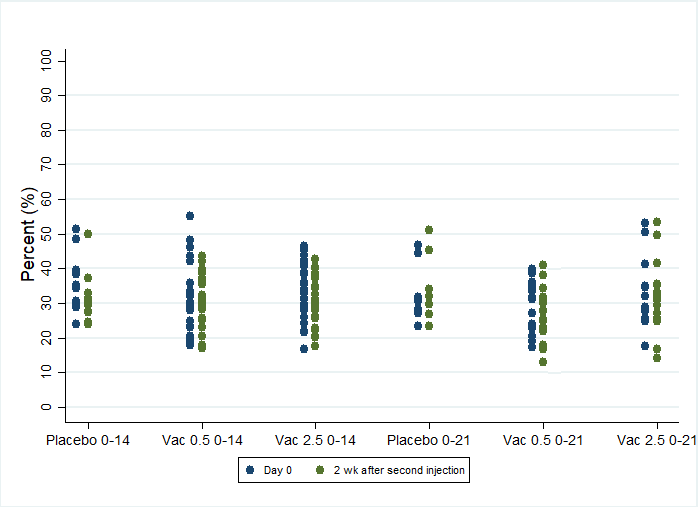


Figure 29. CD4/CD8 ratio within CD3 positive lymphocytic subset in peripheral blood measured by flowcytometry at day 0 and two weeks after 2^nd^ injection in two administration schedules and different vaccine strengths of 0.5 × 10^6^ TCID_50_ (5 µg/dose), 2.5 × 10^6^ TCID_50_ (10 µg/dose) and placebo


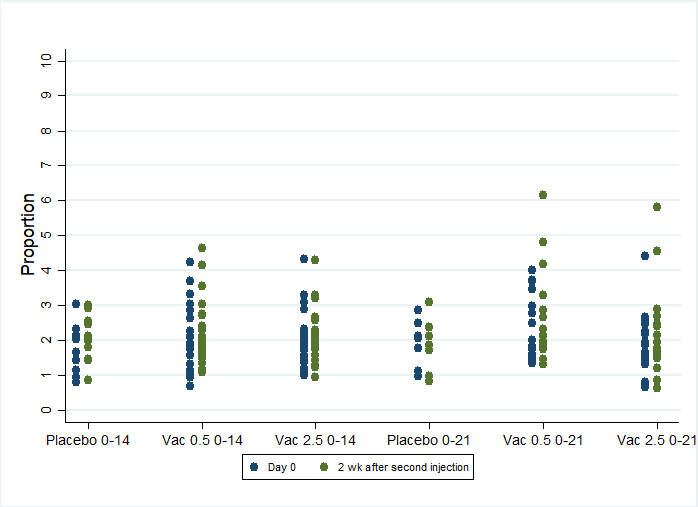


Figure 30. CD4/CD8 ratio within lymphocytic cell population in peripheral blood measured by flowcytometry at day 0 and two weeks after 2^nd^ injection in two administration schedules and different vaccine strengths of 0.5 × 10^6^ TCID_50_ (5 µg/dose), 2.5 × 10^6^ TCID_50_ (10 µg/dose) and placebo


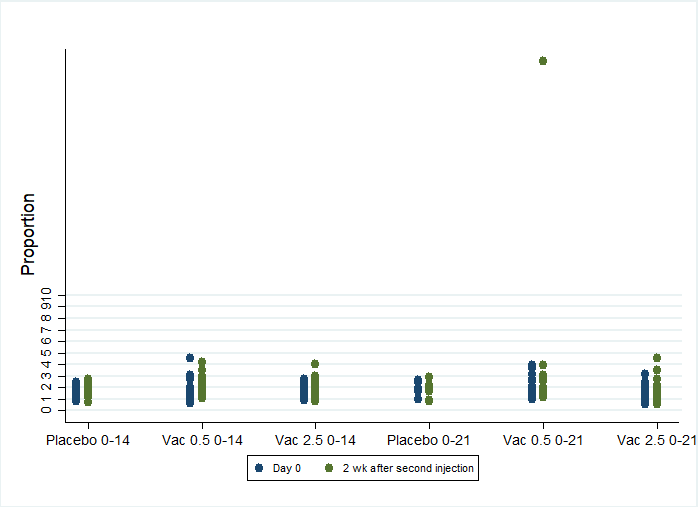


Figure 31. Proportion of lymphocytic population expressing CD56 marker within CD3 negative subset in peripheral blood measured by flowcytometry at day 0 and two weeks after 2^nd^ injection in two administration schedules and different vaccine strengths of 0.5 × 10^6^ TCID_50_ (5 µg/dose), 2.5 × 10^6^ TCID_50_ (10 µg/dose) and placebo


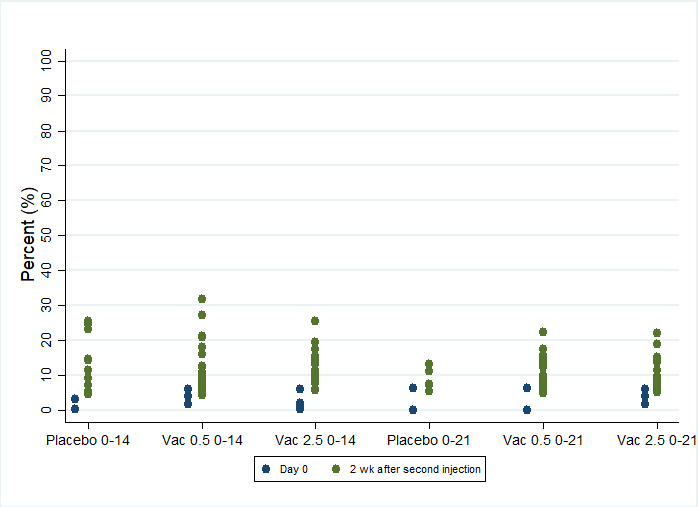


Figure 32. Proportion of CD19 or CD20 in peripheral blood measured by flowcytometry at day 0 and two weeks after 2^nd^ injection in two administration schedules and different vaccine strengths of 0.5 × 10^6^ TCID_50_ (5 µg/dose), 2.5 × 10^6^ TCID_50_ (10 µg/dose) and placebo


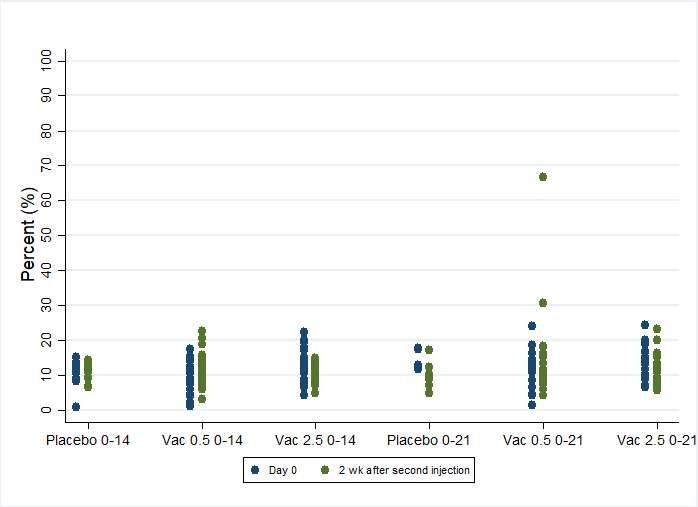

Supplement: Supplementary data 3 [file mmc3.docx]
