# Supplementary material for: Safety and immunogenicity of an inactivated SARS-CoV-2 vaccine (FAKHRAVAC®) in healthy adults aged 18–55 years: Randomized, double-blind, placebo-controlled, phase I clinical trial
Source: Vaccine X. 2023 Oct 27;15:100401. doi: 10.1016/j.jvacx.2023.100401 (PMC10628354; doi:10.1016/j.jvacx.2023.100401)
Supplement: Supplementary data 4 [file mmc4.docx]

**Conventional virus neutralizing test method**

This study used the Vero E6 cell line for virus propagation. Vero E6 cells were cultured in a DMEM medium containing 10% FBS, and then infection and replication of the SARS-CoV-2 virus were performed. The VNT test based on Spearman and Kairber’s method and the 50% Tissue Culture Infectious Dose (TCID50) was performed as follows. At first, a volume of 200 μl of serial-diluted serum (1, 1/2, 1/4, 1.8, 1/16, 1/32, 1/64, 1/128, and 1/256) was prepared in the DMEM medium without FBS. Four wells were considered in a sterile flat-bottom 96-well plate for each serum. Then 200 μl of 400 TCID50 per ml of SARS-CoV-2 (the same virus used for vaccine production) was added to each serum and was incubated at 37 °C for 2 hours. The inoculum was removed, infected cells were washed once with 5% FBS in DMEM, and 100 μl of serum-virus suspension was transferred to each. The final dilutions included 1.2, 1.4, 1.8, 1.16, 1.32, 1.64, 1.128, 1.256, and 1.512 of the serum sample. In this study, positive and negative controls, including cell-free culture medium and virus-containing culture medium, were considered. Then, the microplates were incubated at 36°C and 5% CO2 for three days. The plates were checked daily by microscopic observation. Finally, the ability to neutralize sera antibodies was evaluated against the virus.
